# Associations Between Motor Competence and Physical Activity, Physical Fitness and Psychosocial Characteristics in Adolescents: A Systematic Review and Meta-analysis

**DOI:** 10.1007/s40279-023-01886-1

**Published:** 2023-08-05

**Authors:** Alan M. Burton, Ian Cowburn, Ffion Thompson, Joey C. Eisenmann, Ben Nicholson, Kevin Till

**Affiliations:** 1 Research Centre for Sports Coaching, Carnegie School of Sport, Leeds Beckett University, Headingley Campus, Churchwood Avenue, Leeds, LS26 3QT UK; 2 Queen Ethelburga’s Collegiate, York, UK; 3 Lakeland University, Plymouth, WI USA

## Abstract

**Background:**

Motor competence is an integral component of the health and performance of youth. Numerous studies support the hypothesis that motor competence interacts with perceived motor competence and physical fitness during childhood to induce positive (e.g. healthy weight status) or negative (e.g. reduced physical activity engagement) trajectories. Yet, while adolescence is a key period of rapid growth and maturation, no systematic reviews and meta-analyses have examined the association between motor competence and physical activity, physical fitness and psychosocial characteristics solely within adolescents.

**Objectives:**

This study aimed to (1) analyse the scientific literature evaluating associations between motor competence and physical activity, physical fitness and/or psychosocial characteristics amongst adolescents; (2) evaluate the associations between motor competence and physical activity, physical fitness characteristics and/or psychosocial characteristics amongst adolescents; and (3) investigate the impact of moderator variables (i.e., age, sex, type of motor competence assessment) on the associations.

**Methods:**

A systematic search of electronic databases was conducted, followed by a qualitative synthesis of study methods. Random-effects meta-analyses were performed to establish the magnitude and orientation of pooled correlation coefficients between motor competence and physical activity, physical fitness and psychosocial characteristics of adolescents, whilst considering potential moderators (i.e., age, sex, type of motor competence assessment).

**Results:**

Sixty-one studies were included, totalling 22,256 adolescents. Twenty-seven different assessments of motor competence were used, with 31 studies utilising product-orientated (i.e. outcome) motor competence assessments. Meta-analyses of 43 studies showed that motor competence was positively associated with physical activity (*r* = 0.20 to 0.26), some physical fitness characteristics (e.g. muscular strength, cardiovascular endurance; *r* = 0.03 to 0.60) and psychosocial characteristics (*r* = 0.07 to 0.34), and inversely associated with weight status (*r* =  − 0.36 to − 0.10), speed (*r* =  − 0.31) and agility (*r* =  − 0.37 to 0.41). Associations with flexibility were unclear.

**Conclusions:**

The results of this systematic review and meta-analysis support the hypothesised interactions of motor competence with physical activity (positive), physical fitness (positive except for weight status, speed and agility) and psychosocial characteristics (positive) in adolescence. However, methodological approaches vary considerably (e.g. variety of motor competence assessments utilised), with limitations of the current literature including an inadequate assessment of motor competence, a lack of longitudinal observations and a failure to account for biological maturation. Future research assessing associations between motor competence and physical activity, physical fitness and psychosocial characteristics of adolescents should include longitudinal observations of a combined motor competence assessment (i.e. process and product) and account for biological maturation. Improved evaluation using these recommendations could provide more accurate data, leading to more targeted interventions to improve adolescents’ physical and psychosocial outcomes.

**Clinical Trial Registration:**

CRD42021233441 (PROSPERO ID).

**Supplementary Information:**

The online version contains supplementary material available at 10.1007/s40279-023-01886-1.

## Key Points

**Table:** 

A systematic review of 61 studies indicated several methodological limitations (i.e. an inadequate assessment of motor competence, a lack of longitudinal observations and a failure to account for biological maturation) within the current literature that evaluates associations between motor competence, physical activity, physical fitness and psychosocial characteristics amongst adolescents.
Across several meta-analyses of 43 studies, motor competence was positively associated with physical activity, muscular endurance, muscular power, muscular strength, cardiovascular fitness, perceived motor competence and motivation, and inversely associated with weight status, speed and agility in adolescents.
Teachers, sports coaches, strength and conditioning coaches, and other stakeholders involved in health and performance interventions during adolescence should seek to synergistically develop motor competence, physical fitness and psychosocial characteristics for positive physical activity and health outcomes.

## Introduction

The synergistic development of physical, psychosocial and motor skill domains throughout childhood and adolescence, across various environments, is important for the health and performance of all youth [1]. Such holistic development of “athleticism” (i.e. the composition of health-related fitness and psychosocial traits [1]) is crucial given the worldwide decline in youth health and fitness and therefore athleticism over past decades [2–4], confounded by reduced sports participation rates (e.g. [5, 6]), and fewer youth meeting the World Health Organisation’s ([7]) physical activity guidelines [8]. In turn, these trends may contribute to the increasing obesity pandemic amongst youth (e.g. UK [9], USA [10]).

Authors have postulated that motor competence underpins daily tasks, and engagement in health-enhancing activities (e.g. running, resistance training, recreational games, sport) across the lifespan [11]. Motor competence refers to an individual’s ability to perform a variety of motor skills, where outcomes are influenced by movement quality, control and coordination [12–14]. Furthermore, motor competence consists of simple, combined and complex movement capacities, which are inter-related. Motor competencies are often categorised into locomotor (e.g. running), object control (e.g. striking) and stability (e.g. balance) skills [15–17]; however, other domains (e.g. foundational movement skills, athletic motor skill competencies) have also been proposed [13, 18]. Research highlights that motor competence is crucial for physical and psychosocial development [19], as it enhances children’s and adolescents’ ability to meaningfully participate in games, sports and other physical activities [20]. Therefore, developing motor competence amongst youth should be a key focus of any physical activity, physical education or youth sport intervention, as it appears central to reversing the currently negative physical activity and obesity trends worldwide.

**Fig. 1:**
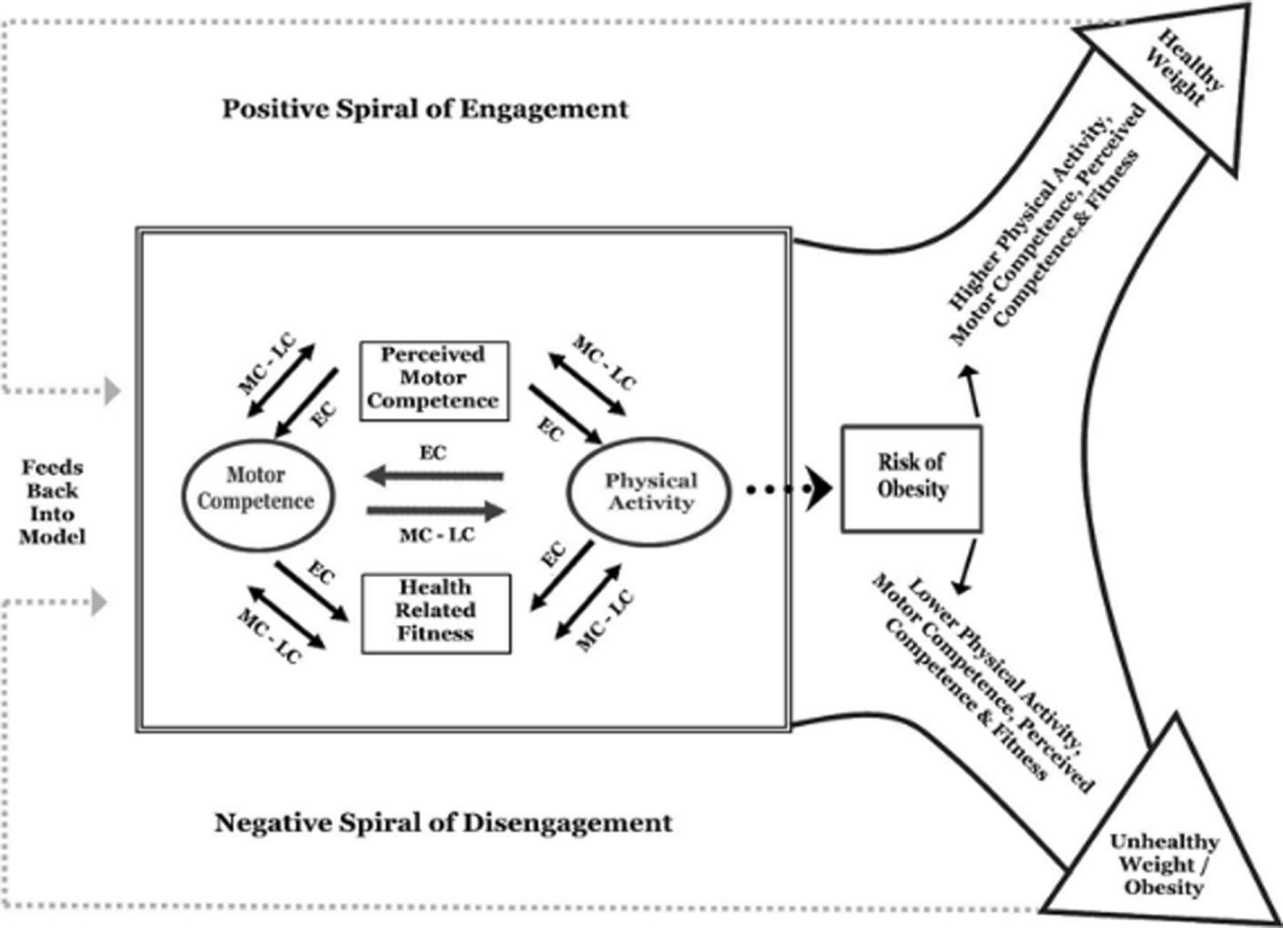
Development model as proposed by Stodden et al. [21]. *EC* early childhood, *LC* late childhood, *MC* middle childhood. Copyright © [2023] National Association for Kinesiology in Higher Education (NAKHE), reprinted by permission of Taylor & Francis Ltd, http://www.tandfonline.com on behalf of © [2023] National Association for Kinesiology in Higher Education (NAKHE)

Previously, Stodden et al. [21] hypothesised that motor competence interacts with perceived motor competence (an individual’s identification and interpretation of their actual motor competence [14, 22]) and physical fitness during childhood to induce positive (e.g. increased physical activity engagement, healthy weight status) or negative (e.g. reduced physical activity engagement, unhealthy weight status) trajectories (Fig. 1). Accordingly, those expressing poor actual and perceived motor competence during childhood may present with reduced actual/perceived motor competence, physical fitness and physical activity engagement across the lifespan [23, 24]. Numerous studies have evaluated Stodden and colleagues [21] model, identifying positive associations between motor competence and physical activity engagement [25–27], musculoskeletal strength/endurance [12], cardiorespiratory fitness [12, 25] and inverse associations with weight status [12, 25]. Similarly, previous reviews (e.g. [14, 28, 29]) have shown that evidence levels differ for associations between different motor skills domains (e.g. locomotor, object control, stability/balance) and physical activity, physical fitness and/or psychosocial characteristics. However, most of the existing evidence involves children (e.g. [30–33]), or children and adolescents together (e.g. [34–36]).

Childhood and adolescence are stages of youth development that require a divergent physical and psychosocial focus [37]. Adolescence represents a dynamic period of physical, psychosocial and highly individual development whereby the timing (i.e. the onset of change), magnitude (i.e. level of change) and tempo (i.e. rate) of biological maturation are asynchronous with chronological age [38, 39]. During biological maturation, growth rate increases rapidly, with peak height velocity (PHV; [38, 39]) typically occurring around 12 years for female individuals and 14 years for male individuals [40, 41]. This growth spurt can lead to temporary reductions in motor competence (i.e. adolescent awkwardness [42]). Furthermore, during adolescence, brain maturation is significant and ongoing. Psychosocial changes include an increased ability to process information [43], and improved executive function of the pre-frontal cortex [44], which underpins many self-regulatory mechanisms (e.g. behavioural/emotional/attentional regulation [45]). Thus, along with physiological changes, adolescents are developing their ability to self-evaluate and problem solve their own physical development. Factors such as age and maturity have been posed to contribute to the globally high percentage of adolescents who do not reach the World Health Organisation’s recommended physical activity guidelines, if appropriate interventions are not implemented [46]. Consequently, investigating motor competence within adolescent populations is an important consideration to enhance the health and athletic development of youths.

To the authors’ knowledge, no systematic review has examined the associations between motor competence and physical activity, physical fitness and psychosocial characteristics within adolescents alone. Further, because of the potential ramifications of the dynamic nature of growth and maturation, adolescence is a key period of the lifespan to focus upon such characteristics. While other reviews have investigated child and adolescent populations simultaneously (e.g. [12, 14, 47]), reporting findings simultaneously in studies may result in misinterpretation owing to a failure to distinguish between children and adolescent findings, leading to an unclear picture of adolescent research (e.g. [12]). Therefore, solely focusing on relevant research in adolescents is warranted to comprehensively review the types of research conducted, methods employed, measures used and the confounding effects these factors may have within this population. Additionally, it remains unclear which characteristics to target across adolescence to optimise health and performance outcomes [1]. Consequently, a systematic review and meta-analysis are required to highlight associations between motor competence and physical activity, physical fitness and psychosocial characteristics in adolescence. Such research will highlight potential focus points (e.g. population, characteristics of interest, methods of assessment) for future implementation and assessment of interventions, which is critical for understanding and potentially reversing the current negative physical activity and fitness trends among adolescents. Therefore, this study aimed to (1) analyse the scientific literature evaluating associations between motor competence and physical activity, physical fitness and/or psychosocial characteristics amongst adolescents; (2) evaluate the associations between motor competence and physical activity, physical fitness characteristics and/or psychosocial characteristics amongst adolescents; and (3) investigate the impact of moderator variables (i.e. age, sex, type of motor competence assessment) on the associations.

## Methods

### Study Design and Search Strategy

A systematic review and meta-analysis were conducted in accordance with the updated Preferred Reporting Items for Systematic reviews and Meta-Analyses (PRISMA) statement [48]. Before commencing the review, the protocol was registered on the International Prospective Register of Systematic Reviews (PROSPERO) database (ref: CRD42021233441). A systematic search of eight databases (Academic Search Complete, CINAHL Complete, MEDLINE, SPORTDiscus and PsycINFO via EBSCOhost, PubMed, SCOPUS and SAGE Journals Online) was conducted to identify original research articles from the earliest record available up to and including 05/08/2022. Boolean search phrases were used to combine search terms relevant to adolescents (population), motor competence, physical activity and/or physical fitness, and/or psychosocial characteristics. Relevant keywords were identified for each search term through pilot searching (screening titles/abstracts, keywords, full texts and similar reviews previously published, e.g. [12, 14, 47].). Keywords were combined for each term using the “OR” operator, and the final search phrase was constructed using the “AND” and “NOT” operators as follows: (“Youth*” OR “Adolescen*” OR “Teen*” OR “Student*” OR “High school” OR “Secondary school” OR “Pube*”) AND (“Motor competenc*” OR “Movement competenc*” OR “Physical competenc*” OR “Motor development” OR “Motor skill*” OR “Motor abilit*” OR “Movement skill*” OR “Motor coordination” OR “Actual competenc*” OR “Object control” OR “Manipulative skill*” OR “Locomotor skill*” OR “Stability skill*” OR “Athletic competenc*” OR “Athletic skill*” OR “Motor proficiency” OR “Fundamental movement skill”) AND (“Physical activit*” OR “Activit*” OR “Sports” OR “Sports participation” OR “Body weight status” OR “Body composition” OR “Body fat” OR “BMI” OR “Physical fitness” OR “Fitness” OR “Cardiorespiratory fitness” OR “Cardiovascular endurance” OR “Muscular strength” OR “Muscular power” OR “Flexibility” OR “Mobility” OR “Endurance” OR “Muscular endurance” OR “psychological” OR “psycho-social” OR “Motivation” OR “Perceived motor competenc*” OR “Physical self-perceptions” OR “Self-confidence” OR “Self-efficacy” OR “Self-Competenc*” OR “physical self-concept”) AND (“correlate*” OR “determinant*” OR “predictor*” OR “relationship*” OR “association*”) NOT (“Adult*” OR “Child*” OR “Prepube*” OR “primary school” OR “Kid” OR “Kids” OR “Preschool” OR “Kindergart*” OR “preadolescen*” OR “Disease*” OR “Disab*” OR “Impair*” OR “Disorder*” OR “ill*”). Bibliographic screening and citation searching are powerful complementary tools to database searching alone [49, 50]. Therefore, bibliographic screening and forward citation searching (via Google Scholar) of previous reviews and included studies were conducted to identify articles that may have been missed by the search criteria.

### Study Selection

Duplicate records were identified and removed before screening the remaining studies against the following pre-defined exclusion criteria: (1) studies not published in English; (2) previous reviews, conference abstracts, book (chapters), dissertations; (3) studies where the sample consists of only children (< 11 years old) or adults (> 18 years old) OR studies that included a combined sample of children/adults with adolescents; (4) participants with a physical or cognitive impairment; (5) studies that did not assess motor competence using a process (i.e. technique; e.g. Test of Gross Motor Development), product (i.e. outcome; e.g. Movement Assessment Battery for Children) or combined method (i.e. process and product; e.g. supine to stand test); (6) studies that did not report the association between motor competence and at least one measure of physical activity (e.g. pedometer, self-report questionnaire), physical fitness (e.g. assessments of body weight status, cardiorespiratory fitness, musculoskeletal strength) or psychosocial characteristics (e.g. perceived motor competence, motivation); and (7) full text not available. The screening process was conducted independently by two researchers (AB and FT) over two phases. Studies were initially excluded based on their title and abstract content, followed by a full-text review. There were no formal disagreements between reviewers regarding study selection; however, reviewers met virtually to discuss and clarify studies where there was more than one reason for exclusion. As there were no formal disagreements between reviewers, a third reviewer was not required.

### Data Extraction

The lead author (AB) extracted the data using a specifically designed and standardised Microsoft Excel spreadsheet. Publication details (e.g. author, year), study type (e.g. cross-sectional, longitudinal, intervention), participant characteristics (i.e. sample size, age, sex, anthropometrics), motor competence assessment details and scores (i.e. measure used, type of measure), physical activity measure details and scores (i.e. measure used, type of measure), physical fitness measure details and scores (i.e. area of physical fitness assessed, measure used), psychosocial measure details and scores (i.e. measure used, psychosocial domain assessed), and the strength and orientation of associations between motor competence and physical activity, physical fitness and psychosocial characteristics were extracted. If any relevant data were missing, the paper’s corresponding author was contacted to provide the required information. Similarly, if the authors had performed a regression analysis on study variables, the authors were contacted to provide a correlation coefficient between the variables in question. Unlike similar reviews (e.g. [14].), reported regression coefficients were not converted to correlation coefficients using the Peterson and Brown [51] equation, as potentially large biases are associated with estimating mean population correlations in meta-analytic conditions [52]. Authors were contacted once in the first instance (followed by one further occasion if there was no response to the original query) for any missing details needed for the meta-analysis. Studies were excluded from the meta-analysis, but still utilised in the qualitative synthesis of the review, if authors did not respond or could not provide the requested information.

### Risk of Bias Assessment

Consistent with previous research (e.g. [12, 14, 25, 47]), the criteria for assessing bias within included studies were adapted from the Strengthening the Reporting of Observation studies in Epidemiology (STROBE) [53] and Consolidated Standards of Reporting Trials (CONSORT) [54] statements. For this review, six criteria were determined to assess the risk of bias within included studies (Table 1). For each criterion, studies were scored with a tick (“✔”, low risk of bias), cross (“✖”, high risk of bias) or question mark (“**?**”, inadequate or unclear description). To create clear criteria and ensure high agreement between reviewers, the first (AB) and second reviewer (FT) individually screened the same five papers and subsequently discussed the scoring criteria via an online meeting. After refining the criteria, the first and second reviewer independently screened all the included studies and reconvened via an online meeting to compare final scores.

**Table 1:** Summary of risk of bias assessment criteria

Statement	Responses
1. Does the study adequately describe participant sampling procedures and inclusion criteria?	✓ Random sampling of target population is used. Participant inclusion criteria are clearly described AND/OR authors clearly outline demographic information of participants (at a minimum, age, sex data reported) ✕ Convenience sampling used. Participant inclusion criteria AND participant demographic information are not presented ? Participant inclusion criteria/sampling method/demographic information is not clearly described
2. Does the study clearly outline the motor competence assessment(s) used (specific measures/procedures/valid)?	✓ Motor competence assessment is clearly outlined, source is referenced, AND validity of the assessment for the target population is clearly stated within the text, OR previous validation study is referenced. If single measure(s) used, full details and validation provided for each measure ✕ Motor competence assessment not outlined or referenced, OR validity of the assessment for the target population is not clearly stated within the text AND previous validation study is not referenced. Single measure(s) are not outlined, and validation data not provided ? Unclear if valid measure used because of inadequate description
3. Does the study provide acceptable reliability information for the motor competence assessment(s) used?	✓ One or more acceptable reliability statistic clearly highlighted (e.g. Cronbach alpha ≥ 0.70 or test–retest reliability an ICC ≥ 0.60, Brown et al. [211]) OR previous reliability of the instrument is clearly stated and referenced ✕ Reliability data not reported OR at least one reliability statistic was not acceptable (e.g. Cronbach alpha < 0.70 or test–retest reliability ICC < 0.60) OR a single item of a motor competence assessment was used to measure reliability AND previous reliability of the instrument is not clearly stated or referenced ? Inadequate description so unclear if reliable measure was used
4. Does the study clearly outline the PA/physical fitness/psychosocial assessment(s) used (specific measures/procedures/valid)?	✓ PA/physical fitness/psychosocial assessment(s) is clearly outlined, source(s) referenced, AND validity of the assessment(s) for the target population is clearly stated within the text, OR a previous validation study is referenced. If single measure(s) used, full details and validation provided for each measure ✕ PA/physical fitness/psychosocial assessment(s) not outlined or referenced, OR validity of the assessment for the target population is not clearly stated within the text AND previous validation study is not referenced. Single measure(s) are not outlined, and validation data not provided ? Unclear if valid measure used because of an inadequate description
5. Does the study provide acceptable reliability information for the PA/physical fitness/psychosocial assessment(s) used?	✓ One or more acceptable reliability statistic clearly highlighted (e.g. Cronbach alpha ≥ 0.70 or test–retest reliability ICC ≥ 0.60, Brown et al. [211]) OR previous reliability of the instrument is clearly stated and referenced ✕ Reliability data not reported OR at least one reliability statistic was not acceptable (e.g. Cronbach alpha < 0.70 or test–retest reliability ICC < 0.60) OR a single-item of a PA, physical fitness or psychosocial assessment was used to measure reliability AND previous reliability of the instrument is not clearly stated or referenced ? Inadequate description so unclear if reliable measure was used
6. Of those who consented to the study, did an adequate proportion have complete data for the motor competence and the PA/physical fitness/psychosocial assessments?	✓ Clearly identifiable from the text or tables that no fewer than 80% (cross-sectional studies) or 70% (longitudinal studies) of participants completed all measures ✕ < 80% (cross-sectional studies) or < 70% (longitudinal studies) of participants completed all measures ? Inadequate description so unclear what percentage of total number of participants completed each assessment

*ICC* intraclass correlation coefficient, *PA* physical activity, ✓ indicates a low risk of bias, ✕ indicates a high risk of bias, ? indicates an inadequate or unclear description

### Data Analysis and Meta-analysis

This review’s qualitative synthesis and interpretation used descriptive data extracted from the articles. Where studies used a reverse scale measure (e.g. [55]), or where time (e.g. [56]) represented an outcome measure of motor competence, the effect size direction was reversed prior to analysis so that the association between variables represented the same orientation as other studies. This step accounted for studies where lower scores represented a greater outcome (e.g. faster time = greater motor competence). Within the meta-analysis, correlations of individual sexes were used where available. Additionally, associations of separate motor competence domains (i.e. overall, locomotor, object control, stability/balance, sports-specific competence) were analysed independently to avoid double counting. The fundamental movement skills concept was selected to define sub-group categories for this meta-analysis during a video call between co-authors (AB, IC, JCE, KT). This concept was clearly used by most studies to determine separate categories for correlations, thus allowing the maximum possible studies to be evaluated in the meta-analyses. Furthermore, the fundamental movement skill domains are widely acknowledged in the practical setting for prescribing and assessing motor skills (e.g. [17]). Studies were included more than once in the same meta-analysis where authors had correlated more than one measure of motor competence to the same variable (e.g. [57]), or had used the same measures on separate samples at different timepoints (e.g. [58]).

Random-effects meta-analyses were conducted using Comprehensive Meta-Analysis software (version 3.0; Biostat, Englewood, NJ, USA) to determine the magnitude, orientation and significance of the association between motor competence and physical activity, motor competence and physical fitness characteristics (e.g. strength, cardiovascular endurance), and motor competence and psychosocial characteristics (e.g. perceived motor competence, motivation). Several meta-analyses were conducted based on the relevant primary studies to explore the effect of hypothesised moderator variables (i.e. sex, age and type of motor competence measure [process, product or combined]) on the variation among study outcomes [59, 60].

The inputted data from each study included the sample size and the corresponding outcome measure (i.e. correlation coefficient). Each correlation coefficient (r) was converted to a Fisher’s *z*-score and standard error to obtain approximately normally distributed values. The Fisher’s z-score was then back transformed to a correlation coefficient and 95% confidence interval (CI) for interpreting the included studies’ summary statistic (i.e. pooled correlation coefficient). Pooled correlation coefficients were estimated for each comparison and moderator variable where possible. Pooled correlation coefficients were interpreted as: 0.00–0.10 (trivial), 0.10–0.30 (small), 0.30–0.50 (moderate), 0.50–0.70 (high), 0.70–0.90 (very high) and > 0.90 (nearly perfect) [61–63]. Statistical significance was interpreted for *p* < 0.05. Cochrane’s Q statistic and *I*^2^ statistic were used to determine heterogeneity, with *I*^2^ values of > 50%, and > 75% used to indicate moderate heterogeneity and high heterogeneity, respectively [64, 65]. The *I*^2^ statistic was supported by reporting the tau-squared statistic. A sensitivity analysis (one study removed function) was used for each comparison, which omitted study samples in turn to examine their influence on the magnitude, orientation or significance of pooled correlation coefficients.

### Evaluation of Small Study Effects

Funnel plots were visually interpreted, along with Egger’s linear regression intercepts for each comparison, to evaluate potential small study effects and publication bias. An Egger statistic *p*-value < 0.05 indicated the presence of a small study effect.

## Results

### Overview of Studies

**Fig. 2:**
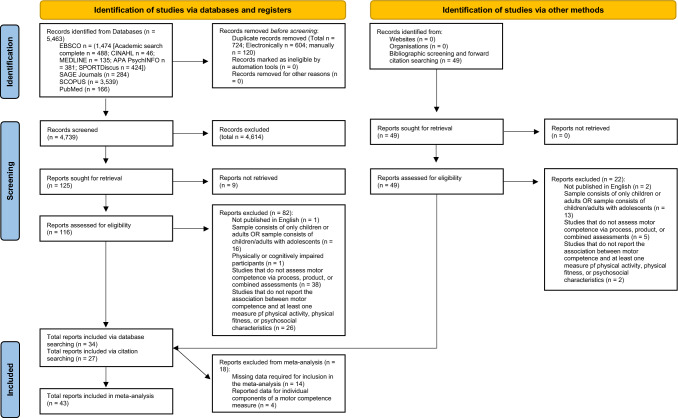
Flow diagram of the study selection process

Following the removal of duplicates, a total of 4739 records were identified via the databases searched. Forty-nine additional records were identified from bibliographical screening and forward citation searching. From the title, abstract and full-text screening, 61 records were identified for the systematic review [36, 55–58, 66–121]. Of the studies identified for the systematic review, 14 [69, 71, 75, 77, 80, 83, 84, 87, 102–104, 115–117] were excluded from the meta-analysis because of missing data (e.g. unreported correlations, lack of sample size information for a reported correlation) required for conducting the meta-analyses (Fig. 2). A further four studies [78, 94, 109, 112] were also ineligible, as they had provided correlation coefficients for individual elements of a motor competence measure (e.g. overhead squat, frisbee competence), which did not correspond to the motor competence domains utilised for the meta-analysis (e.g. locomotor competence, sports-specific competence). Authors of the studies included in the review that were ineligible for the meta-analysis were contacted for the information required to be included in the meta-analysis. These authors either did not respond to our enquiries or could not be reached via their author contact details.

Extracted data from the included studies are presented in Table 2. Forty-five studies consisted of cross-sectional evaluations, ten studies [80, 103–105, 111–116] collected longitudinal evaluations, three studies [102, 117, 119] conducted a randomised controlled trial intervention, and three studies [66, 68, 110] involved validity and reliability methods. The included studies represented a total sample of 22,256 adolescents (mean = 371 ± 614 participants; range = 22–3638). Studies were conducted across 16 countries including Australia (*n* = 10 [66, 67, 71, 73, 74, 78, 83, 89, 110, 119]), Brazil (*n* = 7 [56, 79, 95, 96, 103, 106, 107]), Czech Republic (*n* = 1 [97]), England (*n* = 1 [82]), Finland (*n* = 9 [55, 58, 85, 87, 99, 111–114]), Germany (*n* = 1 [115]), Iceland (*n* = 2 [57, 108]), Ireland (*n* = 6 [70, 75, 76, 91, 102, 117]), New Zealand (*n* = 1 [72]), Norway (*n* = 1 [88]), Portugal (*n* = 1 [116]), Spain (*n* = 1 [98]), Switzerland (*n* = 1 [109]), the UK (*n* = 1 [118]), the USA (*n* = 4 [86, 93, 94, 121]) and Wales (*n* = 1 [100]). The remaining studies (*n* = 13 [36, 68, 69, 77, 80, 81, 84, 90, 92, 101, 104, 105, 120]) provided insufficient detail to determine where the data were collected.

**Table 2:** Overview of included studies

Reference	Study type	Population characteristics	Motor competence assessment(s)	Assessment type	Physical activity assessment(s)	Physical fitness assessment(s)	Psychosocial assessment(s)	Associations
Barnett et al. [67]	Cross-sectional	**Sample** 215 adolescent students (female *n* = 111; male *n* = 104) **Mean age** 16.4 ± 0.6 years	Six out of 12 skills from the Get Skilled Get Active battery [133] **Object control competence** Kick, catch, overarm throw **Locomotor competence** Hop, side gallop, vertical jump	Process	**Self-reported physical activity** The adolescent physical activity recall questionnaire [148]		**Perceived motor competence** The physical self-perception profile [158, 159]	**Locomotor competence** Perceived sports competence: *r* = 0.30, *p* < 0.01 Moderate-vigorous physical activity (MVPA): *r* = 0.14, *p* < 0.05 **Object control competence** Perceived sports competence: *r* = 0.46, *p* < 0.01 MVPA: *r* = 0.35,* p* < 0.01
Britton et al. [104]^a^	Longitudinal	**Sample** 224 adolescents (51% girls) **Mean age** 12.26 ± 0.37 years	Combination of the Test of Gross Motor Development (TGMD)-3 [141] and the Victorian Fundamental movement skills (FMS) manual [125] **Object control competence** Kick, catch, overhand throw, one-hand strike, and two-handed strike **Locomotor competence** Run, skip, horizontal jump, vertical jump **Stability/balance competence** Two-board balance, zigzag hop, and walking heel-to-toe backwards	Process	**Minutes of MVPA per day** Actigraph (models: GT1M, GT3X, GT3X + , wGT3X-BT) accelerometers	**Cardiovascular endurance** FITNESSGRAM [212], EUROFIT manual [213]: 20-m shuttle run **Muscular strength** EUROFIT [213] and HELENA study [214, 215]: horizontal jump HELENA study [214, 215]: vertical jump **Muscular endurance** FITNESSGRAM [212]: push-ups and curl-ups	**Perceived motor competence** The self-perception profile for adolescents [169]	**Object control (first year secondary school)** Physical activity (sixth class primary school): *β* = 0.35,* p* < 0.01 No other direct associations reported
Chagas and Batista [106]	Cross-sectional	**Sample** 68 girls **Age range** 12–14 years **Mean height** 12 years = 1.59 ± 0.03 m 13 years = 1.57 ± 0.03 m 14 years = 1.51 ± 0.01 m **Mean body mass** 12 years = 55.6 ± 4.7 kg 13 years = 50.8 ± 2.9 kg 14 years = 56.7 ± 2.8 kg **Mean BMI** 12 years = 21.9 ± 1.6 kg∙m^2^ 13 years = 20.5 ± 0.7 kg∙m^2^ 14 years = 21.8 ± 1.0 kg∙m^2^ **Mean body fat %** 12 years = 28.6 ± 2.8% 13 years = 25.5 ± 2.1% 14 years = 30.2 ± 2.0%	**Overall competence** Körperkoordinationstest Für Kinder [KTK]) [124]	Product	**Self-reported physical activity** Physical Activity Questionnaire for Older Children (PAQ-C) [147]	**Weight status** Body fat %		**Overall competence** Body fat %: *r* = –0.64, *p* < 0.01
Chagas and Batista [107]	Cross-sectional	**Sample** 56 adolescents (21 boys and 35 girls) **Mean age** 13.7 ± 0.6 years **Mean height** 1.61 ± 0.1 m **Mean body mass** 58.4 ± 16.0 kg **Mean BMI** 22.4 ± 5.0 kg∙m^2^	**Overall competence** KTK [124]	Product	**Self-reported physical activity** Physical Activity Questionnaire for Older Children (PAQ-C) [147]	**Weight status** BMI		**Overall competence** BMI: *r* = − 0.58, *p* < 0.01
Chagas and Batista [79]	Cross-sectional	**Sample** 69 male adolescents **Mean age** 13.7 ± 0.6 years **Age range** 12–14 years	**Overall competence** KTK [124]	Product	**Self-reported physical activity** PAQ-C [147]	**Weight status** Body fat %		**Overall competence** Body fat %: *r* = − 0.37,* p* < 0.01 Physical activity: *r* = 0.24,* p* = 0.05
Chagas and Batista [95]	Cross-sectional	**Sample** 39 adolescents (male *n* = 17; female *n* = 22) **Mean age** 13.7 ± 0.6 years **Mean height** 1.61 ± 0.8 m **Mean body mass** 63.2 ± 17.5 kg	**Overall competence** KTK [124]	Product	**Self-reported physical activity** PAQ-C [147]	**Weight status** BMI		**Overall competence (bivariate correlation)** Weight status:*** r*** = − 0.64,* p* < 0.01 **Overall competence (partial correlations accounting for PA level)** Weight status:*** r*** = − 0.62,* p* < 0.01
Chagas and Marinho [103]	Longitudinal	**Sample** 166 Brazilian middle school pupils (female *n* = 97, male *n* = 69) **Mean age at baseline** Male 13.7 ± 0.6 years, female = 13.7 ± 0.6 years **Mean height at baseline** Male = 1.60 ± 0.1 m, female = 1.59 ± 0.1 m **Body mass at baseline** Male = 49.3 ± 11.9 kg, female = 54.5 ± 14.6 kg	**Overall competence** KTK [124]	Product	**Self-reported physical activity** PAQ-C [147]	**Weight status** Body fat %		**Overall competence** PA level (independent variable): *β* = 3.82 ± 1.57, CI = 0.71, 6.94,* p* < 0.05 Body fat % (dependent variable): *β* = − 0.30 ± 0.03, CI − 0.36, − 0.23,* p* < 0.01
Chagas et al. [96]	Cross-sectional	**Sample** 136 adolescents (67 boys, 69 girls; healthy weight *n* = 100; overweight/obese *n* = 36) **Mean age** Healthy weight group = 13.3 ± 0.6 years; overweight/obese group = 13.3 ± 0.7 years **Mean height** Healthy weight group = 1.58 ± 0.1 m; overweight/obese height = 1.58 ± 0.1 m **Mean body mass** Healthy weight group = 45.7 ± 7.1 kg; overweight/obese group = 68.4 ± 14.9 kg	**Overall competence** KTK [124]	Product		**Weight status** BMI		**Overall competence** Weight status:* r* = − 0.57,* p* < 0.01
Chagas et al. [105]	Longitudinal	**Sample** 122 adolescents (59.8% girls) **Mean age** Male = 13.3 ± 0.5 years; female = 13.2 ± 0.5 years **Mean height** Male = 1.57 ± 0.1 m; female = 1.58 ± 0.1 m **Mean body mass** Male = 47.7 ± 11.2 kg; female = 54.2 ± 15.3 kg	**Overall competence** KTK [124]	Product	**Self-reported physical activity** PAQ-C [147]	**Weight status** BMI **Composite fitness** Sit-ups [216]		**Overall competence** Weight status: *r* = − 0.69, *p* < 0.01 (only reported the association between motor competence and weight status)
Chang et al. [92]	Cross-sectional	**Sample** 32 junior school sport athletes (volleyball *n* = 11; basketball *n* = 12; handball *n* = 9; sex characteristics not reported) **Mean age** 16.06 ± 0.21 years **Mean height** 167.28 ± 6.32 cm **Mean body mass** 68.45 ± 9.67 kg	**Individual motor competence elements** Functional Movement Screen™ [129, 130] **Stability/balance competence** Y-balance test [131]	Combined with scores separated for process (Functional Movement Screen™) and product (Y-balance test) measurements		**Muscular power** Vertical jump **Agility** Agility T-test		**Deep squat** Muscular power:* r* = 0.12,* p* > 0.05 Agility:* r* = − 0.17,* p* > 0.05 **Hurdle step** Muscular power:* r* = 0.06,* p* > 0.05 Agility:* r* = − 0.14,* p* > 0.05 **In line lunge** Muscular power:* r* = 0.06,* p* > 0.05 Agility:* r* = − 0.10,* p* > 0.05 **Shoulder mobility** Muscular power:* r* = − 0.33,* p* > 0.05 Agility:* r* = 0.25,* p* > 0.05 **Straight-leg raise** Muscular power:* r* = − 0.03,* p* > 0.05 Agility:* r* = − 0.01;* p* > 0.05 **Trunk stability push-up** Muscular power:* r* = 0.39,* p* > 0.05 Agility:* r* = − 0.57;* p* < 0.05 **Rotary stability** Muscular power:* r* = 0.35,* p* > 0.05 Agility:* r* = – 0.19,* p* > 0.05 **Stability/balance** Muscular power:* r* = − 0.14,* p* > 0.05 Agility:* r* = − 0.08,* p* > 0.05
Chen and Housner [77]^a^	Cross-sectional from longitudinal follow-up	**Sample** 255 middle school students (male *n* = 136; female *n* = 119) **Mean age** 13.2 ± 1.7 years **Mean height** 63.7 ± 3.6 inches **Mean body mass** 130.4 ± 36.6 lbs	Participants assessed (from process and product perspectives) on dribble, throw, kick, and jump skills from the Test of Gross Motor Development [126]	Combined with scores separated for process and product measurements	**Self-reported physical activity** Unreferenced question asking participants for the number of days per week they engage in sport, fitness or recreational activity	**Weight status** BMI **Muscular endurance** Flexed arm hang, curl-ups **Agility** Shuttle run **Cardiovascular endurance** 1-mile run **Flexibility** Sit and reach		**Dribble (process)** Weight status (whole sample):* r* = − 0.15,* p* < 0.05 (male* r* = − 0.16,* p* > 0.05; female* r* = − 0.14;* p* > 0.05) Muscular endurance: curl-up (whole sample):* r* = 0.06,* p* > 0.05 (male* r* = 0.04,* p* > 0.05; female* r* = 0.06,* p* > 0.05) Muscular endurance: flexed arm hang (whole sample):* r* = 0.13,* p* < 0.05 (male* r* = 0.11,* p* > 0.05; female* r* = 0.13,* p* > 0.05) Agility (whole sample):* r* = − 0.12,* p* > 0.05 (male* r* = − 0.10,* p* > 0.05; female* r* = − 0.12,* p* > 0.05) Cardiovascular endurance (whole sample):* r* = − 0.23;* p* < 0.01 (male:* r* = − 0.24,* p* < 0.01); female:* r* = − 0.20,* p* < 0.05) Flexibility (whole sample):* r* = − 0.06;* p* > 0.05 (male* r* = − 0.10,* p* > 0.05; female* r* = 0.01,* p* > 0.05) **Throw (process)** Weight status (whole sample):* r* = − 0.07,* p* > 0.05 (male* r* = − 0.15,* p* > 0.05; female* r* = − 0.03,* p* > 0.05) Muscular endurance: curl-ups (whole sample):* r* = 0.31,* p* < 0.01 (male* r* = 0.34,* p* < 0.01; female* r* = 0.23,* p* < 0.05) Muscular endurance: flexed arm hang (whole sample):* r* = 0.26,* p* < 0.01 (male* r* = 0.09,* p* > 0.05; female* r* = 0.33,* p* < 0.01) Agility (whole sample):* r* = − 0.29,* p* < 0.01 (male* r* = − 0.36,* p* < 0.01; female* r* = − 0.08,* p* > 0.05) Cardiovascular endurance (whole sample):* r* = − 0.25,* p* < 0.01 (male* r* = − 0.22,* p* < 0.05; female* r* = − 0.05,* p* > 0.05) Flexibility (whole sample):* r* = − 0.03,* p* > 0.05 (male* r* = 0.02,* p* > 0.05; female* r* = 0.13,* p* > 0.05) **Kick (process)** Weight status (whole sample):* r* = − 0.27,* p* < 0.01 (male* r* = − 0.38,* p* < 0.01; female* r* = − 0.19,* p* > 0.05) Muscular endurance: curl-ups (whole sample):* r* = 0.44,* p* < 0.01 (male* r* = 0.39,* p* < 0.01; female* r* = 0.43,* p* < 0.01) Muscular endurance: flexed arm hang (whole sample):* r* = 0.34,* p* < 0.01 (male* r* = 0.29,* p* < 0.01; female* r* = 0.31,* p* < 0.01) Agility (whole sample):* r* = − 0.48,* p* < 0.01 (male* r* = − 0.40,* p* < 0.01; female* r* = − 0.45,* p* < 0.01) Cardiovascular endurance (whole sample):* r* = − 0.46,* p* < 0.01 (male* r* = − 0.43,* p* < 0.01; female* r* − 0.33,* p* < 0.01) Flexibility (whole sample):* r* = 0.01,* p* > 0.05 (male* r* = − 0.03,* p* > 0.05; female* r* = 0.23,* p* < 0.05) **Jump (process)** Weight status (whole sample):* r* = − 0.14,* p* > 0.05 (male* r* = − 0.24,* p* < 0.01; female* r* = − 0.04,* p* > 0.05) Muscular endurance: curl-ups (whole sample):* r* = 0.31,* p* < 0.01 (male* r* = 0.36,* p* < 0.01; female* r* = 0.17,* p* > 0.05) Muscular endurance: flexed arm hang (whole sample):* r* = 0.26,* p* < 0.01 (male* r* = 0.27,* p* < 0.01; female* r* = 0.16,* p* > 0.05) Agility (whole sample):* r* = − 0.38,* p* < 0.01 (male* r* = − 0.38,* p* < 0.01; female* r* = − 0.32,* p* < 0.01) Cardiovascular endurance (whole sample):* r* = − 0.29,* p* < 0.01 (male* r* = − 0.24,* p* < 0.01; female* r* = − 0.26,* p* < 0.01) Flexibility (whole sample):* r* = 0.12,* p* > 0.05 (male* r* = 0.12,* p* > 0.05; female* r* = 0.24,* p* < 0.05) **Dribble (product)** Weight status (whole sample):* r* = 0.35,* p* < 0.01 (male* r* = 0.37,* p* < 0.01; female* r* = 0.36;* p* < 0.01) Muscular endurance: curl-ups (whole sample):* r* = − 0.42,* p* < 0.01 (male* r* = − 0.39,* p* < 0.01; female* r* = − 0.39,* p* < 0.01) Muscular endurance: flexed arm hang (whole sample):* r* = − 0.35,* p* < 0.01 (male* r* = − 0.36,* p* < 0.01; female* r* = − 0.21,* p* < 0.05) Agility (whole sample):* r* = 0.57,* p* < 0.01 (male* r* = 0.55,* p* < 0.01; female* r* = 0.52,* p* < 0.01) Cardiovascular endurance (whole sample):* r* = 0.55;* p* < 0.01 (male* r* = 0.56,* p* < 0.01; female* r* = 0.41,* p* < 0.01) Flexibility (whole sample):* r* = − 0.06;* p* > 0.05 (male* r* = − 0.13,* p* > 0.05; female* r* = 0.20,* p* > 0.05) **Throw (product)** Weight status (whole sample):* r* = − 0.12,* p* > 0.05 (male* r* = − 0.29,* p* < 0.01; female* r* = 0.00,* p* > 0.05) Muscular endurance—curl-ups (whole sample):* r* = 0.46,* p* < 0.01 (male* r* = 0.51,* p* < 0.01; female* r* = 0.35,* p* < 0.01) Muscular endurance: flexed arm hang (whole sample):* r* = 0.35,* p* < 0.01 (male* r* = 0.36,* p* < 0.01; female* r* = 0.16,* p* > 0.05) Agility (whole sample):* r* = − 0.56,* p* < 0.01 (male* r* = − 0.59,* p* < 0.01; female* r* = − 0.38,* p* < 0.01) Cardiovascular endurance (whole sample):* r* = − 0.51,* p* < 0.01 (male* r* = − 0.47,* p* < 0.01; female* r* = − 0.29,* p* < 0.01) Flexibility (whole sample):* r* = − 0.05,* p* > 0.05 (male* r* = 0.20,* p* < 0.05; female* r* = 0.20,* p* < 0.05) **Kick (product)** Weight status (whole sample):* r* = − 0.06,* p* > 0.05 (male* r* = − 0.11,* p* > 0.05; female* r* = − 0.03,* p* > 0.05) Muscular endurance—curl-ups (whole sample):* r* = 0.35,* p* < 0.01 (male* r* = 0.25,* p* < 0.01; female* r* = 0.41,* p* < 0.01) Muscular endurance: flexed arm hang (whole sample):* r* = 0.26,* p* < 0.01 (male* r* = 0.20,* p* < 0.01; female* r* = 0.20,* p* < 0.01) Agility (whole sample):* r* = − 0.42,* p* < 0.01 (male* r* = − 0.33,* p* < 0.01; female* r* = − 0.38,* p* < 0.01) Cardiovascular endurance (whole sample):* r* = − 0.39,* p* < 0.01 (male* r* = − 0.31,* p* < 0.01; female* r* = − 0.28* p* < 0.01) Flexibility (whole sample):* r* = 0.06,* p* > 0.05 (male* r* = − 0.11,* p* > 0.05; female* r* = 0.25,* p* < 0.01) **Jump (product)** Weight status (whole sample):* r* = − 0.44,* p* < 0.01 (male* r* = − 0.50*, p* < 0.01; female* r* = − 0.41,* p* < 0.01) Muscular endurance: curl-ups (whole sample):* r* = 0.48,* p* < 0.01 (male* r* = 0.47,* p* < 0.01; female* r* = 0.41,* p* > 0.05) Muscular endurance: flexed arm hang (whole sample):* r* = 0.60,* p* < 0.01 (male* r* = 0.61,* p* < 0.01; female* r* = 0.50,* p* < 0.01) Agility (whole sample):* r* = − 0.69,* p* < 0.01 (male* r* = − 0.71,* p* < 0.01; female* r* = − 0.60,* p* < 0.01) Cardiovascular endurance (whole sample):* r* = − 0.60,* p* < 0.01 (male* r* = − 0.55,* p* < 0.01; female* r* = − 0.52,* p* < 0.01) Flexibility (whole sample):* r* = 0.16,* p* < 0.05 (male* r* = 0.21,* p* < 0.05; female* r* = 0.41,* p* < 0.01)
De Meester et al. [36]^b^	Cross-sectional	**Sample** 215 adolescents (male *n* = 142; female *n* = 73) **Mean age** 13.64 ± 0.58 years **Age range** 12.42–14.92 years	**Overall competence** KTK [124]	Product	**Self-reported physical activity** Flemish Physical Activity Questionnaire [149]		**Motivation** Dutch version of Behavioural Regulation in Physical Education Questionnaire [156] **Perceived motor competence** Sport/athletic competence subscale [161] of the of the Children and Youth Physical Self-Perception Profile (Dutch version) [162]	**Overall motor competence** Perceived motor competence:* r* = 0.30,* p* < 0.01 Autonomous motivation:* r* = 0.20,* p* < 0.01 Minutes per week engaging in MVPA:* r* = 0.09,* p* > 0.05
Deprez et al. [80]^a^	Longitudinal	**Sample** 162 Flemish male youth soccer players **Mean age at baseline** 12.2 ± 1.3 years	3 subsets of the KTK [124]: Moving sideways, backward balancing, and jumping sideways	Product		**Cardiovascular endurance** Yo-yo intermittent recovery test level 1		**Stability/balance competence** Cardiovascular endurance:* r* = 0.21,* p-*value not reported
Estevan et al. [98]	Cross-sectional	**Sample** 236 students **Mean age** 13.01 ± 0.72 years **Age range** 11–14 years	**Overall competence** KTK [124]	Product			**Perceived motor competence** Pictorial Scale of Perceived Motor Skill Competence [160]; separate scale for male and female individuals **Self-determined motivation for physical education** Spanish version of the Perceived Locus of Causality Scale [157]	**Overall competence** Perceived motor competence:* r* = 0.37 Motivation:* r* = 0.15 *p-*Values not reported
Estevan et al. [68]	Validity and reliability	**Sample** 904 adolescent students (47.6% female) *n* = 91 completed the actual motor competence assessment **Age range** 11–14 years	**Overall competence** KTK [124]	Product			**Perceived motor competence** Pictorial Scale of Perceived Motor Skill Competence in Stability Skills [68] and Pictorial Scale of Perceived Motor Skill Competence [160]	**Overall motor competence** Perceived stability competence:* r* = 0.51,* p* < 0.01 Perceived locomotor competence* r* = 0.47,* p* < 0.01 Perceived object control competence* r* = 0.32,* p* < 0.01
Fu and Burns [121]	Cross-sectional	**Sample** 66 sixth grade students (36 girls, 30 boys) **Mean age** 11.6 ± 0.5 years	**Overall competence** TGMD-3 [141]	Process	**School daily step count** Yamax DigiWalker CW600 (Tokyo, Japan) pedometers		**Perceived motor competence** Perceived Competence Scale for Children [170] **Physical activity enjoyment** Sport Enjoyment Scale [174] **Self-efficacy** 6-item scale [176]	**Overall competence** School daily step count: *r* = 0.33, *p* < 0.05 Perceived motor competence: *r* = 0.37, *p* < 0.05 Physical activity enjoyment: *r* = − 0.08, *p* > 0.05 Self-efficacy: *r* = − 0.12, *p* > 0.05
Gísladóttir et al. [108]	Cross-sectional	**Sample** 94 adolescent secondary school students (46 girls, 48 boys) **Mean age** Whole group = 15.9 ± 0.30 years Male = 15.8 ± 0.30 years Female = 15.9 ± 0.30 years	**Overall competence** Movement Assessment Battery for Children-2 (MABC-2) [136]	Product		**Composite fitness score** Test of physical fitness [217, 218]: standing broad jump, 20-m sprint, reduced Cooper run EUROFIT: sit-and-reach [213]		**Overall competence (whole sample)** Composite fitness score: *r* = 0.25, *p* < 0.05 **Overall competence (male)** Composite fitness score: *r* = 0.28, *p* > 0.05 **Overall competence (female)** Composite fitness score: *r* = 0.35, *p* < 0.05
Gísladóttir et al. [57]	Cross-sectional	**Sample** 101 adolescents (94 adolescents completed all measures; male *n* = 48; female *n* = 46) **Mean age** 15.9 ± 3.63 years (male = 15.9 ± 3.63 years; female = 15.8 ± 3.63 years) **Age range** 15.4–16.3 years	**Overall competence** MABC-2 [136]; Test of Motor Competence (TMC) [138]	Product		**Composite fitness score** Three assessments from the test of physical fitness [218]: standing broad jump, 20-m sprint, reduced Cooper run; one test from the EUROFIT test battery [213]: sit and reach		**Overall competence (MABC-2; whole sample)** Composite fitness score:* r* = 0.28,* p* < 0.01 **Overall competence (MABC-2; female)** Composite fitness score:* r* = 0.35,* p* < 0.01 **Overall competence (MABC-2; male)** Composite fitness score:* r* = 0.33,* p* < 0.05 **Overall competence (TMC; whole sample)** Composite fitness score:* r* = − 0.36,* p* < 0.01) **Overall competence (TMC; female)** Composite fitness score:* r* = − 0.34,* p* < 0.05 **Overall competence (TMC; male)** Composite fitness score:* r* = − 0.40,* p* < 0.01
Gu et al. [93]	Prospective study design across one academic year	**Sample** 279 adolescents (female *n* = 148; male* n* = 131) **Mean age** 12.49 ± 0.89 years	**Sports-specific competence** Physical education Metrics battery [132]	Process		**Weight status** BMI **Muscular endurance** Abdominal curl-ups, 90-degree push-ups **Cardiovascular endurance** PACER test **Flexibility** Sit and reach		**Sports-specific competence** Weight status:* r* = − 0.15,* p* < 0.01 Muscular endurance:* r* = 0.36,* p* < 0.01 Cardiovascular endurance:* r* = 0.38,* p* < 0.01 Flexibility:* r* = − 0.07,* p* > 0.05
Gu et al. [94]^c^	Prospective study design across one academic year	**Sample** 330 middle school students (male *n* = 154; female *n* = 176) **Mean age** 12.52 ± 0.86 years	**Individual elements of sports-specific competence** PE Metrics battery [132]: volleyball, soccer, frisbee competence	Process	**School-based MVPA** Accelerometery (actical activity monitors)	**Weight status** BMI **Muscular endurance** Abdominal curl-ups, 90-degree push-ups **Cardiovascular endurance** PACER test **Flexibility** Sit and reach		**Volleyball competence** School-based MVPA:* r* = 0.26,* p* < 0.01 Weight status:* r* = 0.01,* p* > 0.05 Muscular endurance:* r* = 0.25,* p* < 0.01 Cardiovascular endurance:* r* = 0.23,* p* < 0.01 **Soccer competence** School based MVPA:* r* = 0.33,* p* < 0.01 Weight status:* r* = − 0.14,* p* < 0.05 Muscular endurance:* r* = 0.20,* p* < 0.01 Cardiovascular endurance:* r* = 0.27,* p* < 0.01 **Frisbee competence** School based MVPA:* r* = 0.25,* p* < 0.01 Weight status:* r* = − 0.05,* p* > 0.05 Muscular endurance:* r* = 0.16,* p* < 0.01 Cardiovascular endurance:* r* = 0.31,* p* < 0.01
Hands et al. [74]	Cross-sectional study of wider longitudinal study from birth	**Sample** 1585 adolescents (female *n* = 771; male *n* = 814) **Mean age** 14.1 ± 0.2 years	**Overall competence** McCarron Assessment of Neuromuscular Development (MAND) [134]	Combined	**Mean daily step count** Yamax digiwalker SW200 pedometers	Six measures from the Australian fitness education award [219] **Weight status** BMI **Muscular endurance** Curl-ups **Muscular strength** Chest pass **Cardiovascular endurance** Physical work capacity 170 test **Flexibility** Sit and reach		**Overall competence** Physical activity (female):* r* = − 0.01,* p* > 0.05 Physical activity (male):* r* = − 0.01,* p* > 0.05 Weight status (female):* r* = − 0.13,* p* < 0.01 Weight status (male):* r* = − 0.08,* p* < 0.05 Muscular endurance (female):* r* = 0.27,* p* < 0.01 Muscular endurance (male):* r* = 0.37,* p* < 0.01 Muscular strength (female):* r* = 0.33,* p* < 0.01 Muscular strength (male):* r* = 0.43,* p* < 0.01 Cardiovascular endurance (female):* r* = 0.15,* p* < 0.01 Cardiovascular endurance (male):* r* = 0.22,* p* < 0.01 Flexibility (sit and reach left leg; female):* r* = 0.23,* p* < 0.01 Flexibility (sit and reach left leg; male):* r* = 0.25,* p* < 0.01 Flexibility (sit and reach right leg; female):* r* = 0.22,* p* < 0.01 Flexibility (sit and reach right leg; male):* r* = 0.28,* p* < 0.01 Flexibility (sit and reach both legs; female):* r* = 0.22,* p* < 0.01 Flexibility (sit and reach both legs; male):* r* = 0.26,* p* < 0.01
Haugen et al. [88]	Cross-sectional study of 9th graders in 2005 and 2008	**Sample** 1839 students (female *n* = 889; male *n* = 950) **Age** 15 years	**Stability/balance competence** One subset (side-to-side jump) of the KTK [124]	Product	**Self-reported physical activity** Unreferenced self-reported physical activity questionnaire	**Weight status** BMI **Muscular power** Standing broad jump **Muscular strength** Push-up test **Cardiovascular endurance** Multi-stage fitness test **Flexibility** Sit and reach	**Perceived motor competence** Norwegian version of Harter’s Self-Perception Profile for Adolescents [165, 166]	**Stability/balance competence (female)** Self-reported physical activity:* r* = 0.26,* p* < 0.01 Weight status:* r* = − 0.25,* p* < 0.01 Muscular strength:* r* = 0.40,* p* < 0.01 Muscular power:* r* = 0.50,* p* < 0.01 Cardiovascular endurance:* r* = 0.48,* p* < 0.01 Flexibility:* r* = 0.23,* p* < 0.01 Perceived motor competence:* r* = 0.34,* p* < 0.01 **Stability/balance competence (male)** Self-reported physical activity:* r* = 0.09,* p* < 0.01 Weight status:* r* = − 0.25,* p* < 0.01 Muscular strength:* r* = 0.37,* p* < 0.01 Muscular power:* r* = 0.38,* p* < 0.01 Cardiovascular endurance:* r* = 0.37,* p* < 0.01 Flexibility:* r* = 0.10,* p* < 0.05 Perceived motor competence:* r* = 0.28,* p* < 0.01
Herrmann and Seelig [109]^c^	Cross-sectional	**Sample** 310 students (147 boys, 163 girls) **Mean age** 11.26 ± 0.49 years	**Individual elements of motor competence** Motorische Basiskompetenzen (MOBAK) [142–144]: Throwing, catching, bouncing, dribbling, balancing, rolling, skipping, running	Product			**Perceived motor competence** Selbstwahrnehmung der motorischen Kompetenz (SEMOK) [109]	**Throwing competence** Perceived throwing competence: *r* = 0.30, *p* < 0.01 **Catching competence** Perceived catching competence: *r* = 0.42, *p* < 0.01 **Bouncing competence** Perceived bouncing competence: *r* = 0.29, *p* < 0.01 **Dribbling competence** Perceived dribbling competence: *r* = 0.37, *p* < 0.01 **Balancing competence** Perceived balancing competence: *r* = 0.33, *p* < 0.01 **Rolling competence** Perceived rolling competence: *r* = 0.52, *p* < 0.01 **Skipping competence** Perceived skipping competence: *r* = 0.44, *p* < 0.01 **Running competence** Perceived running competence: *r* = 0.10, *p* > 0.05
Huhtiniemi et al. [99]	Cross-sectional	**Sample** 645 participants Grade 5 *n* = 328 (50% male); grade 8 *n* = 317 (47.3% male) **Mean age** Grade 5 = 11.2 ± 0.36; grade 8 = 14.2 ± 0.35	**Locomotor competence** 5-leaps test [220] **Object control** Throwing and catching combination test [220]	Product		**Cardiovascular endurance** PACER test **Muscular endurance** Curl-ups and press-ups	**Enjoyment** The Finnish version of the Enjoyment subscale of the Sport Commitment Questionnaire-2 (SCQ-2) [221] **Anxiety** The Finnish version of the Physical Education State Anxiety Scale (PESAS) [222] **Perceived motor competence** Finnish version of the sport competence dimension in the Physical Self-Perception Profile [159]	Motor competence (i.e. locomotor/object control) only correlated with psychosocial and muscular endurance measures during analysis **Locomotor competence** Enjoyment:* r* = 0.07,* p* > 0.05 Cognitive processes:* r* = − 0.08,* p* > 0.05 Somatic anxiety:* r* = − 0.14,* p* < 0.01 Worry:* r* = − 0.13,* p* < 0.01 Perceived motor competence:* r* = 0.27,* p* < 0.01 Curl-ups:* r* = 0.28,* p* < 0.01 Push-ups:* r* = 0.44,* p* < 0.01 **Object control competence** Enjoyment:* r* = 0.21,* p* < 0.01 Cognitive processes:* r* = − 0.20,* p* < 0.01 Somatic anxiety:* r* = − 0.12,* p* < 0.01 Worry:* r* = − 0.11,* p* < 0.01 Perceived motor competence:* r* = 0.36,* p* < 0.01 Curl-ups:* r* = 0.28,* p* < 0.001 Push-ups:* r* = 0.32,* p* < 0.001
Hulteen et al. [110]	Validity and reliability	**Sample** 109 students; 55 boys, 54 girls **Mean age** All = 15.82 ± 0.37 years Male = 15.85 ± 0.31 years Female = 15.79 ± 0.43 years **Mean height** All = 170.82 ± 8.92 cm Male = 176.60 ± 7.30 cm Female = 164.94 ± 6.17 cm **Mean body mass** All = 67.68 ± 13.27 kg Male = 70.05 ± 13.67 kg Female = 65.17 ± 12.48 kg **Mean BMI** All = 23.11 ± 3.99 kg∙m^2^ Male = 22.43 ± 4.13 kg∙m^2^ Female = 23.84 ± 3.73 kg∙m^2^	**Overall competence** Life-Long Physical Activity Skills Battery [145]	Process	**Moderate-vigorous physical activity (min/day)** GENEActiv wrist-worn accelerometers (Model GAT04, Activinsights Ltd, Cambridgeshire, England)	**Weight status** BMI **Muscular power** Standing long jump [223] **Muscular endurance** 90° push-up test [175] **Cardiovascular endurance** 3-min step test [224] **Flexibility** Back-saver sit and reach [225]		**Overall competence** MVPA (min/day): *r* = 0.21, *p* > 0.05 BMI Z-score: *r* = − 0.12, *p* > 0.05 Muscular power: *r* = 0.43, *p* < 0.01 Muscular endurance: *r* = 0.30, *p* < 0.01 Cardiovascular endurance: *r* = 0.32, *p* < 0.01 Flexibility: *r* = 0.14, *p* > 0.05
Huotari et al. [58]	Cross-sectional (two separate samples at 2 different timepoints)	**2003 sample** 2348 (male *n* = 1167; female *n* = 1181) **2010 sample** 1290 (male *n* = 656; female *n* = 634) **Mean age (male)** 2003 sample = 15.2 ± 0.4 years; 2010 sample = 15.3 ± 0.4 years **Mean age (female)** 2003 sample = 15.1 ± 0.3 years; 2010 sample = 15.2 ± 0.4 years **Mean height (male)** 2003 sample = 174.3 ± 7.0 cm; 2010 sample = 175.7 ± 7.7 cm **Mean height (female)** 2003 sample = 164.4 ± 5.9 cm; 2010 sample = 165.3 ± 6.3 cm **Mean body mass (male)** 2003 sample = 64.2 ± 10.8 kg; 2010 sample = 66.9 ± 12.3 kg **Mean body mass (female)** 2003 sample = 56.2 ± 8.8 kg; 2010 sample = 57.2 ± 9.9 kg	**Object control competence** Figure 8 dribble test [226] **Stability/balance competence** Lateral jumping test from the KTK [124] **Locomotor and stability competence** Motor coordination track test [227] **Overall competence** All measures combined to form an FMS index	Product	**Self-reported physical activity** Leisure Time Physical Activity Questionnaire [150]	**Weight status** BMI		**Overall competence** Organised physical activity: 2003 sample* r* = 0.42,* p* < 0.01; 2010 sample* r* = 0.45,* p* < 0.001 Unorganised physical activity: 2003 sample* r* = 0.27,* p* < 0.01; 2010 sample* r* = 0.19,* p* < 0.01 Physical activity index: 2003 sample* r* = 0.29,* p* < 0.01; 2010 sample* r* = 0.30,* p* < 0.01 Weight status: 2003 sample* r* = − 0.13,* p* < 0.01; 2010 sample* r* = − 0.19,* p* < 0.01 **Object control competence** Organised physical activity: 2003 sample;* r* = 0.44,* p* < 0.01; 2010 sample* r* = 0.44,* p* < 0.01 Unorganised physical activity: 2003 sample* r* = 0.21,* p* < 0.01; 2010 sample* r* = 0.19,* p* < 0.01 Physical activity index: 2003 sample* r* = 0.22,* p* < 0.01; 2010 sample* r* = 0.19,* p* < 0.01 Weight status: 2003 sample* r* = − 0.07,* p* < 0.05; 2010 sample* r* = − 0.13,* p* < 0.01 **Stability/balance competence** Organised physical activity: 2003 sample* r* = 0.29,* p* < 0.01; 2010 sample* r* = 0.37,* p* < 0.01 Unorganised physical activity: 2003 sample* r* = 0.21,* p* < 0.01; 2010 sample* r* = 0.14,* p* < 0.01 Physical activity index: 2003 sample* r* = 0.20,* p* < 0.01; 2010 sample* r* = 0.26,* p* < 0.01 Weight status: 2003 sample* r* = − 0.14,* p* < 0.01. 2010 sample* r* = − 0.14,* p* < 0.01 **Locomotor and stability/balance competence** Organised physical activity: 2003 sample* r* = 0.32,* p* < 0.01; 2010 sample* r* = 0.29,* p* < 0.01 Unorganised physical activity: 2003 sample* r* = 0.22,* p* < 0.01; 2010 sample* r* = 0.18,* p* < 0.01 Physical activity index: 2003 sample* r* = 0.27,* p* < 0.01; 2010 sample* r* = 0.29,* p* < 0.01 Weight status: 2003 sample* r* = − 0.21,* p* < 0.01; 2010 sample* r* = − 0.20,* p* < 0.01
Jaakkola et al. [87]^a^	Cross-sectional	**Sample** 152 grade 7 Finnish students (female* n* = 76; male *n* = 76) **Age** 13 years	**Locomotor competence** Leaping test [228] **Object control competence** Accuracy of throwing [229] **Stability/balance competence** Flamingo standing test [213]	Product	**Self-reported physical activity** Unreferenced self-reported physical activity questionnaire			**Locomotor competence (male)** Self-reported physical activity (leisure):* r* = 0.18,* p* > 0.05 Self-reported physical activity (sports club):* r* = 0.37*, p* < 0.01) Self-reported physical activity (TV/computer time):* r* = − 0.28,* p* < 0.05) **Locomotor competence (female)** Self-reported physical activity (leisure):* r* = 0.26,* p* < 0.05 Self-reported physical activity (sports club):* r* = 0.55,* p* < 0.01 Self-reported physical activity (tv/computer time):* r* = − 0.14,* p* > 0.05 **Object control competence (male)** Self-reported physical activity (leisure):* r* = 0.08,* p* > 0.05 Self-reported physical activity (sports club): *r* = 0.19,* p* > 0.05 Self-reported physical activity (TV/computer time):* r* = 0.09,* p* > 0.05) **Object control competence (female)** Self-reported physical activity (leisure):* r* = − 0.03,* p* > 0.05 Self-reported physical activity (sports club):* r* = 0.10,* p* > 0.05 Self-reported physical activity (tv/computer time):* r* = 0.05,* p* > 0.05 **Stability/balance competence (male)** Self-reported physical activity (leisure):* r* = 0.10,* p* > 0.05 Self-reported physical activity (sports club):* r* = 0.43,* p* < 0.01 Self-reported physical activity (tv/computer time):* r* = 0.22,* p* > 0.05 **Stability/balance competence (female)** Self-reported physical activity (leisure):* r* = 0.18,* p* > 0.05 Self-reported physical activity (sports club):* r* = 0.48,* p* < 0.01 Self-reported physical activity (tv/computer time):* r* = − 0.03;* p* > 0.05
Jaakkola and Washington [112]^c^	Longitudinal	**Sample** 152 grade 7 Finnish students (girls *n* = 66, boys* n* = 86) **Age** 13 years	A combination of individual measures [213, 228, 230] **Stability/balance competence** Flamingo standing test, rolling test **Locomotor competence** Leaping test, shuttle running test, rope jumping test **Object control competence** Figure 8 dribbling test, accuracy throwing test	Product	**Self-reported physical activity** The Health Behaviour in School-Aged Children Survey [151]			**Flamingo standing test** Female days/week physically active: *r* = 0.04, *p* > 0.05 Male days/week physically active: *r* = 0.04, *p* > 0.05 **Rolling** Female days/week physically active: *r* = 0.08, *p* > 0.05 Male days/week physically active: *r* = 0.11, *p* > 0.05 **Shuttle running** Female days/week physically active: *r* = 0.10, *p* > 0.05 Male days/week physically active: *r* = 0.15, *p* > 0.05 **Rope jumping** Female days/week physically active: *r* = 0.08, *p* > 0.05 Male days/week physically active: *r* = 0.07, *p* > 0.05 **Leaping** Female days/week physically active: *r* = 0.03, *p* > 0.05 Male days/week physically active: *r* = 0.05, *p* > 0.05 **Accuracy throwing** Female days/week physically active: *r* = 0.14, *p* > 0.05 Male days/week physically active: *r* = 0.10, *p* > 0.05 Figure 8** dribbling** Female days/week physically active: *r* = 0.06, *p* > 0.05 Male days/week physically active: *r* = 0.01, *p* > 0.05
Jaakkola et al. [114]	Longitudinal	**Sample** 333 grade 7 students (female *n* = 200, male *n* = 133) **Mean age** Total = 12.41 ± 0.27 years Female = 12.38 ± 0.25 years Male = 12.60 ± 0.31 years **Mean height** Total = 159.01 ± 7.36 cm Female = 159.17 ± 6.45 cm Male = 158.85 ± 8.20 cm **Mean body mass** Total = 48.49 ± 10.08 kg Female = 48.04 ± 9.59 kg Male = 48.91 ± 10.53 kg **Mean BMI** Total = 19.15 ± 3.27 kg∙m^2^ Female = 18.96 ± 3.20 kg∙m^2^ Male = 19.33 ± 3.33 kg∙m^2^	**Locomotor competence** Leaping test [231] **Object control competence** Figure 8 dribble test [226] **Stability/balance** Flamingo standing test [213]	Product	**Self-reported physical activity** International Physical Activity Questionnaire Short Form (IPAQ) [152]	**Composite fitness score** 12-min Cooper run (male) or 1500 m run (female), and curl-up test [228, 232]		**Overall competence** Light physical activity: *r* = 0.27, *p* < 0.01 Moderate physical activity: *r* = 0.27, *p* < 0.01 Vigorous physical activity: *r* = 0.43, *p* < 0.01 Composite fitness: *r* = 0.23, *p* < 0.01 BMI: *r* = − 0.05, *p* > 0.05
Jaakkola et al. [111]	Longitudinal	**Sample** 336 students (163 girls and 173 boys) **Mean age** All = 12.03 ± 0.38 years	A combination of individual measures **Locomotor competence** 5-leap test [228] **Object control competence** Throwing and catching combination test [220]	Product	**Moderate-physical physical activity (min/day)** Accelerometers (Actigraph GT3X + or wGT3X +)			**Locomotor competence** Female MVPA: r = 0.24, *p* < 0.01 Male MVPA: r = 0.22, *p* < 0.05 **Object control competence** Female MVPA: r = 0.18, *p* > 0.05 Male MVPA: r = 0.25, *p* < 0.05
Jaakkola et al. [113]	Longitudinal	**Sample** 491 Finnish physical education students (girls = 275, boys = 216) **Mean age** Total = 11.26 ± 0.33 years Female = 11.26 ± 0.33 years Male = 11.27 ± 0.33 years **Mean height** Total = 148.35 ± 6.83 cm Female = 148.07 ± 7.18 cm Male = 148.49 ± 6.36 cm **Mean body mass** Total = 41.64 ± 8.94 kg Female = 41.34 ± 8.57 kg Male = 42.03 ± 9.40 kg **Mean BMI** Total BMI = 18.82 ± 3.06 kg∙m^2^ Female = 18.74 ± 2.96 kg∙m^2^ Male = 18.92 ± 3.19 kg∙m^2^	A combination of individual measures **Locomotor competence** 5-leaps test [220] **Object control competence** Throwing-catching combination test [220] **Stability/balance competence** Two-legged jumping from side-to-side test [124]	Product	**Moderate-physical physical activity (min/day)** Accelerometers (Actigraph GT3X +)	**Muscular endurance** Curl-ups and press-ups [220] **Cardiovascular endurance** PACER test [233]		**Locomotor competence** Female MVPA: *r* = 0.28, *p* < 0.01 Female muscular endurance: *r* = 0.56, *p* < 0.01 Female cardiovascular endurance: *r* = 0.60, *p* < 0.01 Male MVPA: *r* = 0.39, *p* < 0.01 Male muscular endurance: *r* = 0.47, *p* < 0.01 Male cardiovascular endurance: *r* = 0.61, *p* < 0.01 **Object control competence** Female MVPA: *r* = 0.24, *p* < 0.01 Female muscular endurance: *r* = 0.33, *p* < 0.01 Female cardiovascular endurance: *r* = 0.45, *p* < 0.01 Male MVPA: *r* = 0.41, *p* < 0.01 Male muscular endurance: *r* = 0.36, *p* < 0.01 Male cardiovascular endurance: *r* = 0.55, *p* < 0.01 **Stability/balance competence** Female MVPA: *r* = 0.26, *p* < 0.01 Female muscular endurance: *r* = 0.55, *p* < 0.01 Female cardiovascular endurance: *r* = 0.49, *p* < 0.01 Male MVPA: *r* = 0.33, *p* < 0.01 Male muscular endurance: *r* = 0.48, *p* < 0.01 Male cardiovascular endurance: *r* = 0.58, *p* < 0.01
Jekauc et al. [115]^a^	Longitudinal	**Sample** 698 German adolescents (335 boys and 363 girls) **Mean age** 14.2 ± 2.0 years	A combination of individual measures **Stability/balance competence** Jumping side to side, single leg stance, and backward balancing (unreferenced)	Product	**Self-reported physical activity** MoMo Physical Activity Questionnaire (MoMo-PAQ) for adolescents [153]	**Muscular power** Standing long jump [234] **Muscular strength** Push -p test [235] **Cardiovascular endurance** Physical Working Capacity 170 cycle ergometry test [236, 237] **Flexibility** Singular forward bend (unreferenced)	**Perceived motor competence (measured at follow-up only)** Physical Self-Description Questionnaire [171, 172]	No correlation or multiple regression data at baseline
Kalaja et al. [55]	Cross-sectional	**Sample** 316 Finnish grade 7 students (female *n* = 162; male *n* = 154) **Age** 13 years	**Locomotor competence** Leaping test [228] **Object control competence** Figure 8 dribble test [226] **Stability/balance competence** Flamingo standing test [213]	Product	**Self-reported physical activity** Unreferenced self-reported physical activity questionnaire. Final score was expressed as the number of minutes per week engaging in physical activity		**Motivation** Sports Motivation Scale (Finnish version) [155] **Perceived motor competence** Sports Competence Subscale of the Physical Self-Perception Profile (Finnish version) [158, 159]	**Locomotor competence** Self-reported physical activity:* r* = 0.08,* p* > 0.05 Motivation:* r* = 0.15,* p* < 0.01 Perceived competence:* r* = − 0.22,* p* < 0.01 **Object control competence** Self-reported physical activity:* r* = 0.10,* p* > 0.05 Motivation:* r* = 0.07,* p* > 0.05 Perceived competence:* r* = − 0.22,* p* < 0.01 **Stability/balance competence** Self-reported physical activity:* r* = − 0.04,* p* > 0.05 Motivation:* r* = − 0.20,* p* < 0.01 Perceived competence:* r* = 0.15,* p* < 0.01
Kalaja et al. [85]	Cross-sectional	**Sample** 370 grade 7 students (female *n* = 189; male *n* = 181) **Mean age** 13.08 ± 0.25 years	**Locomotor competence** Leaping test [231] **Object control competence** Figure 8 dribble test [226] **Stability/balance** Flamingo standing test [213]	Product			**Motivation** Sports Motivation Scale (Finnish version) [155] **Perceived motor competence** Sports Competence Subscale of the Physical Self-Perception Profile (Finnish version) [158, 159]	**Locomotor competence** Motivation:* r* = 0.15,* p* < 0.01 Perceived motor competence:* r* = 0.22,* p* < 0.001 **Object control competence** Motivation:* r* = 0.10,* p* > 0.05 Perceived motor competence:* r* = 0.22,* p* < 0.001 **Stability/balance competence** Motivation:* r* = 0.20,* p* < 0.001 Perceived motor competence:* r* = 0.15,* p* < 0.01
Kokstejn et al. [97]	Cross-sectional	**Sample** 40 U12 male soccer players **Mean age** 11.5 ± 0.3 years **Mean height** 145 ± 7 cm **Mean body mass** 37.2 ± 4.1 kg	**Overall competence** Bruininks-Oseretsky Test of Motor Proficiency-2 short form (BOT-2 short) [128]	Product		**Composite fitness score** Three measures from the Unifittest 6–60 [238, 239]		**Overall competence** Composite fitness score:* r* = 0.50,* p* < 0.01
Kovac et al. [101]	Cross-sectional	**Sample** 258 female volleyball players **Mean age** 14.3 ± 1.7 years **Mean height** 168.1 ± 8.4 cm **Mean body mass** 58.3 ± 10.0 kg	**Overall competence** Functional Movement Screen™ [129, 130]	Process		**Weight status** BMI		**Overall competence** BMI: *r* = 0.04,* p* > 0.05 **Deep squat** BMI:* r* = − 0.12,* p* < 0.05 **Hurdle step** BMI:* r* = − 0.11,* p* > 0.05 **In line lunge** BMI:* r* = − 0.04,* p* > 0.05 **Shoulder mobility** BMI:* r* = 0.01,* p* > 0.05 **Active straight leg raise** BMI:* r* = 0.13,* p* < 0.05 **Trunk stability push-up** BMI:* r* = 0.14,* p* < 0.05 **Rotary stability** BMI:* r* = 0.03,* p* > 0.05
Kramer et al. [86]	Cross-sectional	**Sample** 56 high school athletes (male *n* = 28; female *n* = 28) **Mean age** 16.4 ± 0.1 (male = 16.8 ± 0.9 years; female = 16.0 ± 0.9 years) **Mean height** Male = 177.4 ± 8.6 cm; female = 165.2 ± 8.1 cm **Mean body mass** Male = 78.2 ± 18.0 kg; female = 58.7 ± 8.0 kg	**Overall competence** Functional Movement Screen™ [129, 130] **Stability/balance competence** Y-balance test [131]	Combined with scores separated for process (Functional Movement Screen™) and product (Y-balance test) measurements		**Muscular power** Standing long jump, vertical jump **Agility** Pro agility test		**Overall competence (process; male)** Muscular power (standing long jump):* r* = 0.25,* p* > 0.05 Muscular power (vertical jump):* r* = 0.26,* p* > 0.05 Agility:* r* = − 0.44,* p* < 0.05) **Overall competence (process; female)** Muscular power (standing long jump):* r* = − 0.23,* p* > 0.05 Muscular power (vertical jump):* r* = − 0.25,* p* > 0.05 Agility:* r* = − 0.08,* p* > 0.05 **Stability/balance competence (product; male)** Muscular power (standing long jump):* r* = 0.00,* p* > 0.05 Muscular power (vertical jump):* r* = 0.14,* p* > 0.05 Agility:* r* = − 0.10,* p* > 0.05 **Stability/balance competence (product; female)** Muscular power (standing long jump):* r* = 0.07,* p* > 0.05 Muscular power (vertical jump):* r* = − 0.19,* p* > 0.05 Agility:* r* = − 0.45,* p* < 0.05
Lloyd et al. [118]	Cross-sectional	**Sample** 33 male academy football players **Mean age** Under 11 = 11.2 years ± 0.5 years Under 13 = 13.2 years ± 0.2 years Under 16 = 15.6 years ± 0.7 years **Mean height** Under 11 = 146.0 ± 4.7 cm Under 13 = 157.6 ± 9.0 cm Under 16 = 177.0 ± 4.1 cm **Mean body mass** Under 11 = 72.0 ± 2.9 kg Under 13 = 75.8 ± 5.5 kg Under 16 = 87.5 ± 2.6 kg	**Overall competence** Functional Movement Screen™ [129, 130]	Process		**Muscular power** Squat jump test (jump height) and maximal rebounding rest (for reactive strength index) [240] **Reactive agility** Reactive agility test [241]		**Overall competence** Squat jump: *r* = 0.66, *p* < 0.01 Reactive strength index: *r* = 0.74, *p* < 0.01 Reactive agility: *r* = − 0.54, *p* < 0.01 **Dowel overhead squat:** Squat jump: *r* = 0.49, *p* < 0.01 Reactive strength index: *r* = 0.57, *p* < 0.01 Reactive agility: *r* = − 0.40, *p* < 0.05 **In line lunge** Squat jump: *r* = 0.43, *p* < 0.01 Reactive strength index: *r* = 0.70, *p* < 0.01 Reactive agility: *r* = − 0.60, *p* < 0.05 **Hurdle step** Squat jump: *r* = 0.43, *p* > 0.05 Reactive strength index: *r* = 0.46, *p* < 0.01 Reactive agility: *r* = − 0.27, *p* > 0.05 **Active straight leg raise** Squat jump: *r* = 0.58, *p* < 0.01 Reactive strength index: *r* = 0.65, *p* < 0.01 Reactive agility: *r* = − 0.59, *p* < 0.01 **Shoulder mobility** Squat jump: *r* = 0.40, *p* < 0.01 Reactive strength index: *r* = 0.50, *p* < 0.01 Reactive agility: *r* = − 0.35, *p* > 0.05
Lopes et al. [116]^a^	Longitudinal	**Sample** 103 adolescents (53 female) **Mean age** 13.49 ± 0.87 years **Mean BMI** 20.16 ± 3.34 kg∙m^2^	**Overall competence** KTK [124]	Product	**Objective physical activity** Accelerometers (ActiGraph GT1M)	**Weight status** BMI		**Overall competence at baseline** Light physical activity at follow-up: *β* = − 0.24, *p* = > 0.05 Moderate physical activity at follow-up: *β* = 0.02, *p* > 0.05 Moderate-vigorous physical activity at follow-up: *β* = 0.05, *p* < 0.05 Vigorous physical activity at follow-up: *β* = 0.02, *p* < 0.05 Total physical activity at follow-up: *β* = 0.01, *p* > 0.05
Lubans et al. [66]	Validity and reliability	**Sample** 63 adolescent school students (44 male and 19 female) **Mean age** 14.5 ± 1.2 years **Mean height** 1.67 ± 0.09 m **Mean body mass** 59.3 ± 11.3 kg	**Overall competence:** Resistance Training Skills Battery (RTSB) [66]	Process		**Composite fitness score** Muscular fitness score (summed standardised scores from a handgrip test, timed push-up test, standing long jump test)		**Overall motor competence** Composite fitness score:* r* = 0.40,* p* < 0.01
McGrane et al. [70]	Cross-sectional	**Sample** 395 adolescents (male *n* = 199; female *n* = 196) *n* = 309 completed FMS and PSCS (male *n* = 157; female *n* = 152) **Mean age** 13.78 ± 1.2 years	**Overall competence** 12 measures from the TGMD-2 [127]: run, hop, gallop, slide, leap, horizontal jump, catch, kick, throw, dribble, strike and roll. Two measures from the TGMD [126]: skip and vertical jump. One measure from the Victorian FMS Manual [125]: balance	Process			**Perceived motor competence** Physical Self-Confidence Scale [177]	**Overall competence** Perceived motor competence (whole sample):* r* = 0.22,* p* < 0.01 Perceived motor competence (female):* r* = 0.31,* p* < 0.01 Perceived motor competence (male):* r* = 0.101,* p* > 0.05
McGrane et al. [75]^a^	Cross-sectional	**Sample** 584 adolescents (male *n* = 278, female *n* = 306) **Mean age** 13.78 ± 0.42 years **Age range** 12.82–15.25 years	**Locomotor competence, object control competence, stability/balance competence** 12 measures from the TGMD-2 [127]: run, hop, gallop, slide, leap, horizontal jump, catch, kick, throw, dribble, strike and roll. Two measures from the TGMD [126]: skip and vertical jump. One measure from the Victorian FMS Manual [125]: balance	Process	**Mean daily minutes spent engaging in moderate-vigorous physical activity** Actigraph GT1M, GT3X, or GT3X + accelerometers		**Perceived motor competence** Physical Self-Confidence Scale [177]	**Locomotor competence** Perceived locomotor competence: *β* = − 0.01, SE = 0.03, 95% CI − 0.07, 0.06,* p* > 0.05 MVPA: *β* = 0.54, SE = 0.26, 95% CI = 0.03, 1.05,* p* < 0.05 **Object control competence** Perceived object control competence: *β* = 0.05, SE = 0.03, 95% CI − 0.02, 0.12,* p* > 0.05 MVPA: *β* = 0.16, SE = 0.28, 95% CI − 0.40, 0.71,* p* > 0.05 **Stability/balance competence** Perceived stability/balance competence: *β* = 0.07, SE = 0.48, 95% CI − 0.03, 0.18,* p* > 0.05 MVPA: *β* = − 0.45, SE = 0.51, 95% CI − 1.46, 0.55,* p* > 0.05
McGrane et al. [117]^a^	1-year randomised controlled trial	**Sample** 482 adolescents (intervention group: female = 120, male = 116; control: female = 116, male = 130) **Mean age** Intervention = 12.77 ± 0.41 years Control = 12.78 ± 0.42 years **Mean BMI** Intervention = 20.43 ± 3.30 kg∙m^2^ Control = 19.79 ± 3.02 kg∙m^2^	**Locomotor competence, object control competence, stability/balance competence** 12 measures from the TGMD-2 [127]: run, hop, gallop, slide, leap, horizontal jump, catch, kick, throw, dribble, strike and roll. Two measures from the TGMD [126]: skip and vertical jump. One measure from the Victorian FMS Manual [125]: balance	Process	**Mean daily minutes spent engaging in moderate-vigorous physical activity** ActiGraph GT1M, GT3X, or GT3X + accelerometers	**Cardiovascular endurance** Queens College 3-min step test [242]		**Overall competence** Intervention × weight status (normal weight): *β* = 4.07, *p* < 0.01 Intervention × weight status (overweight/obese): *β* = 4.04, *p* < 0.01 Intervention × physical activity level (active): *β* = 4.03, *p* < 0.01 Intervention × physical activity level (active): *β* = 4.06, *p* < 0.01 **Locomotor competence** Intervention × weight status (normal weight): *β* = 1.65, *p* < 0.05 Intervention × weight status (overweight/obese): *β* = 2.25, *p* < 0.01 Intervention × physical activity level (active): *β* = 2.18, *p* = 0.01 Intervention × physical activity level (active): *β* = 2.07, *p* < 0.01 **Object control competence** Intervention × weight status (normal weight): *β* = 2.41, *p* < 0.01 Intervention × weight status (overweight/obese): *β* = 1.95, *p* < 0.01 Intervention × physical activity level (active): *β* = 1.95, *p* = 0.01 Intervention × physical activity level (active): *β* = 2.13, *p* < 0.01
Nikolaos [69]^a^	Cross-sectional	**Sample** 22 amateur male basketball players **Mean age** 15.33 ± 0.48 years **Mean height** 174.38 ± 9.03 cm **Mean body mass** 72.56 ± 16.07 kg	**Stability/balance competence** Unreferenced measure: time taken to complete a full clockwise rotation of single leg hops around 9 boxes in a 3 × 3 m grid on each leg	Product		**Weight status** Body fat %, BMI (bioelectrical impedance) **Muscular endurance** Mean of 15 continuous jumps **Muscular power** Vertical jump **Speed** 10-m sprint		**Stability/balance competence (left leg)** BMI:* r* = 0.36,* p* < 0.10 Speed:* r* = 0.71, *p* < 0.01 No data available for body fat %, muscular endurance, muscular power **Stability/balance competence (right leg)** Body fat %:* r* = 0.42,* p* < 0.05 BMI:* r* = 0.45,* p* < 0.05 Muscular endurance:* r* = − 0.42,* p* < 0.05 Muscular power:* r* = − 0.42,* p* < 0.05 Speed:* r* = 0.71,* p* < 0.01
Nunez-Gaunaurd et al. [84]^a^	Cross-sectional	**Sample** 86 middle school children (male *n* = 47; female *n* = 39) **Mean age** 12.22 ± 1.0 years	**Overall competence** BOT-2 short [128]	Product	**Frequency, intensity and duration of physical activity** 7-day accelerometery (StepWatch step activity monitor)	**Weight status** BMI **Muscular endurance** Timed sit-to-stand test [243] **Cardiovascular endurance** 6-min walk test [244] **Functional mobility** Timed up-and-down stairs test [245]		**Overall competence** Weight status (healthy weight group):* r* = 0.02,* p* > 0.05 Weight status (overweight and obese group):* r* = − 0.47,* p* < 0.05
O’Brien et al. [91]	Cross-sectional study of baseline data from longitudinal study	**Sample** 85 adolescents (male *n* = 54; female *n* = 31) **Mean age** 12.86 years (male = 12.94 ± 0.33 years; female = 12.75 ± 0.43 years) **Mean body mass** Male = 51.14 ± 11.75 kg; female = 47.60 ± 9.48 kg	**Overall competence, locomotor competence, object control competence, stability/balance competence** One measure from TGMD [126]: skip; 12 measures from TGMD-2 [127]: run, gallop, hop, leap, horizontal jump, slide, striking a stationary ball, stationary dribble, catch, kick, overhand throw, underhand roll; two measures from Get Skilled Get Active [133]: vertical jump, static balance	Process	**Mean daily minutes spent in moderate-vigorous PA** Actigraph GT1M or GT3X accelerometers	**Weight status** BMI		**Overall competence (male)** Moderate physical activity minutes per day:* r* = − 0.13,* p* > 0.05 Vigorous physical activity minutes per day:* r* = 0.01,* p* > 0.05 MVPA minutes per day:* r* = − 0.06,* p* > 0.05 Weight status:* r* = − 0.45,* p* < 0.01 **Overall competence (female)** Moderate physical activity minutes per day:* r* = 0.08,* p* > 0.05 Vigorous physical activity minutes per day:* r* = 0.36,* p* < 0.05 MVPA minutes per day:* r* = 0.24,* p* > 0.05 Weight status:* r* = − 0.27,* p* > 0.05 **Locomotor competence (male)** Moderate physical activity minutes per day:* r* = − 0.07,* p* > 0.05 Vigorous physical activity minutes per day:* r* = 0.06,* p* > 0.05 MVPA minutes per day:* r* = 0.01,* p* > 0.05 Weight status:* r* = − 0.37,* p* < 0.01 **Locomotor competence (female)** Moderate physical activity minutes per day:* r* = 0.14,* p* > 0.05 Vigorous physical activity minutes per day:* r* = 0.37,* p* < 0.05 MVPA minutes per day:* r* = 0.28,* p* > 0.05 Weight status:* r* = − 0.34,* p* < 0.05 **Object control competence (male)** Moderate physical activity minutes per day:* r* = − 0.23,* p* > 0.05 Vigorous physical activity minutes per day:* r* = − 0.07,* p* > 0.05 MVPA minutes per day:* r* = − 0.15,* p* > 0.05 Weight status:* r* = − 0.17,* p* > 0.05 **Object control competence (female)** Moderate physical activity minutes per day:* r* = 0.06,* p* > 0.05 Vigorous physical activity minutes per day:* r* = 0.25,* p* > 0.05 MVPA minutes per day:* r* = 0.17,* p* > 0.05 Weight status:* r* = 0.06,* p* > 0.05 **Stability/balance competence (male)** Moderate physical activity minutes per day:* r* = − 0.05,* p* > 0.05 Vigorous physical activity minutes per day:* r* = 0.02,* p* > 0.05 MVPA minutes per day:* r* = − 0.01,* p* > 0.05 Weight status:* r* = − 0.49,* p* < 0.01 **Stability/balance competence (female)** Moderate physical activity minutes per day:* r* = − 0.21,* p* > 0.05 Vigorous physical activity minutes per day:* r* = − 0.14,* p* > 0.05 MVPA minutes per day:* r* = − 0.19,* p* > 0.05 Weight status:* r* = 0.14,* p* > 0.05
Okely et al. [73]	Cross-sectional	**Sample** 2026 adolescents (male *n* = 1081 [grade 8 *n* = 557; grade 10 *n* = 524]; female *n* = 945 [grade 8 *n* = 515; grade 10* n* = 430]) **Mean age** Grade 8 = 13.3 years Grade 10 = 15.3 years	**Overall competence** 6 FMS assessed from the Victorian FMS manual: run, vertical jump, catch overhand throw, forehand strike and kick [125]	Process		**Cardiovascular endurance** PACER test [233]		**Overall competence** Cardiovascular endurance: Grade 8 male* r* = 0.33,* p* < 0.01; Grade 8 female* r* = 0.45,* p* < 0.01; Grade 10 male* r* = 0.40,* p* < 0.01; Grade 10 female* r* = 0.50,* p* < 0.01
Okely et al. [71]^a^	Cross-sectional	**Sample** 1847 high school students (Grade 8 n = 985 [male *n* = 517; female *n* = 465]; grade 10 *n* = 862 [male *n* = 470; female *n* = 392]) **Mean age** Grade 8 = 13.3 years Grade 10 = 15.3 years	**Overall competence** 6 measures from the Victorian FMS manual (run, vertical jump, catch overhand throw, forehand strike, and kick) [125]	Process	**Organised and unorganised physical activity (min/week)** Unreferenced self-reported physical activity questionnaire			**Overall competence** Time spent engaging organised physical activity: F(4,1831) = 14.3;* p* < 0.01; R^2^ = 0.03 Time engaging in unorganised physical activity: F(4,1832) = 2.18;* p* > 0.05; R^2^ = 0.005
Philpott et al. [76]^b^	Cross-sectional study from a larger longitudinal study	**Sample** 373 s-level adolescents (female *n* = 178; male *n* = 195 male; first year *n* = 101; second year *n* = 149; third year *n* = 123) **Mean age** 14.38 ± 0.87 years	**Overall competence** 10 skills from TGMD [126], TGMD-2 [127] and the Victorian FMS Manual [125]: horizontal jump, vertical jump, run, skip, catch, stationary dribble, overhead throw, two-handed strike, kick, stability Functional Movement Screen™ [129, 130]	Process			**Perceived motor competence** Physical Self-Confidence Scale [177]	**Overall competence** Perceived motor competence: first year* r* = 0.29,* p* < 0.01; second year* r* = 0.28,* p* < 0.01; third year* r* = 0.41,* p* < 0.01 **Overall competence (Functional Movement Screen™)** Perceived motor competence: first year* r* = 0.25;* p* < 0.01; second year* r* = 0.26,* p* < 0.05; third year* r* = 0.09,* p* > 0.05
Philpott et al. [102]^a^	8-week randomised controlled trial intervention	**Sample** 324 adolescents (female *n* = 149, male *n* = 175; control female *n* = 95, male *n* = 107; intervention female *n* = 95, male *n* = 92) **Mean age** 14.5 ± 0.88 years (control female = 14.28 ± 0.85 years, male = 14.34 ± 0.91 years; intervention female = 14.27 ± 0.89 years, male = 14.63 ± 0.79 years	**Overall competence** 10 skills from TGMD [126], TGMD-2 [127], and the Victorian FMS Manual [125]: horizontal jump, vertical jump, run, skip, catch, stationary dribble, overhead throw, two-handed strike, kick, stability	Process			**Perceived motor competence** Physical Self-Confidence Scale [177]	**Overall competence** Perceived motor competence (post-intervention): *β* = 0.24,* p* < 0.01
Pichardo et al. [72]	Cross-sectional	**Sample** 108 circa-PHV male individuals **Mean age** 13.9 ± 0.5 years **Age range** 13–14 years **Mean seated height** 85.9 ± 5.2 cm **Mean standing height** 166.1 ± 9.4 cm **Mean body mass** 57.6 ± 13.9 kg	**Overall competence** RTSB [66]	Process		**Speed** 10-m, 20-m, and 30-m sprint **Muscular power** Bilateral horizontal jump, countermovement jump and seated medicine ball throw **Muscular strength** Isometric mid-thigh pull (IMTP; absolute and relative peak force) **Cardiovascular endurance** Yo-yo Intermittent Recovery Test-Level 1		**Overall motor competence** 10-m sprint:* r* = − 0.21,* p* < 0.05 20-m sprint:* r* = − 0.37,* p* < 0.01 30-m sprint:* r* = − 0.37,* p* < 0.01 Horizontal jump:* r* = 0.09,* p* > 0.05 Countermovement jump:* r* = 0.11,* p* > 0.05 Seated medicine ball throw:* r* = 0.21,* p* > 0.05 IMTP (absolute):* r* = 0.18,* p* > 0.05 IMTP (relative):* r* = 0.27,* p* < 0.01 Yo-yo intermittent recovery test-level 1:* r* = 0.28,* p* < 0.05
Pullen et al. [100]	Cross-sectional	**Sample** 224 secondary school students (male *n* = 119; female *n* = 105) **Mean age** Male = 11.8 ± 1/6 years; female = 11.8 ± 2.1 years **Mean height** Male = 152.8 ± 9.6 cm; female = 153.8 ± 7.4 cm **Mean body mass** Male = 47.3 ± 13.0 kg; female = 48.9 ± 11.3 kg	**Overall competence** Athlete Introductory Movement Screen (AIMS) [139] and tuck jump assessment [140]	Process		**Weight status** BMI **Muscular power** Standing long jump [246]	**Motivation** Behavioural Regulations in Exercise Questionnaire Version 2 (BREQ-2) [154] **Perceived motor competence** Perceived Physical Ability Scale for Children [168] **Global self-esteem** Rosenberg Self-Esteem Scale [173]	**Overall competence** Muscular power (male):* r* = 0.43,* p* < 0.01 Muscular power (female):* r* = 0.40,* p* < 0.01 BMI (male):* r* = − 0.18,* p* < 0.05 BMI (female):* r* = − 0.18,* p* < 0.05 Motivation (male):* r* = 0.25,* p* < 0.01 Motivation (female):* r* = 0.26,* p* < 0.01 Perceived competence (male):* r* = 0.34,* p* < 0.01 Perceived competence (female):* r* = 0.19,* p* > 0.01 Global self-esteem (male):* r* = 0.13,* p* > 0.05 Global self-esteem (female):* r* = 0.03,* p* > 0.05
Rigoli et al. [81]	Cross-sectional	**Sample** 93 adolescents (male *n* = 55; female *n* = 38) **Mean age** 14.2 ± 1.1 years **Age range** 12–16 years	**Object control, stability/balance competence** MABC-2 [136]	Product			**Perceived motor competence** Self-Description Questionnaire-II [163]	**Object control competence** Perceived physical ability:* r* = 0.46,* p* < 0.01 Physical appearance:* r* = 0.35,* p* < 0.01 Same-gender peer relations:* r* = 0.12,* p* > 0.05 Parent relations:* r* = 0.05,* p* > 0.05 School:* r* = 0.15,* p* > 0.05 Self-concept:* r* = 0.29,* p* < 0.01 **Stability/balance competence** Perceived physical ability:* r* = 0.42,* p* < 0.01 Physical appearance:* r* = 0.27,* p* < 0.05 Same-gender peer relations:* r* = 0.27,* p* < 0.01 Parent relations:* r* = 0.14,* p* > 0.05 School:* r* = 0.21,* p* < 0.05 Self-concept:* r* = 0.35,* p* < 0.01
Rogers et al. [89]	Cross-sectional study of baseline data from randomised controlled trial	**Sample** 173 female students **Mean age** 12.48 ± 0.34 years	**Overall competence, locomotor competence and object control competence** The Victorian FMS Teacher’s Manual [125]	Process			**Perceived motor competence** Physical Self-Perception Profile (PSPP) [159]; Pictorial Scale of Perceived Movement Skill Competence [160]	**Overall competence** Perceived motor competence:* r* = 0.30,* p* < 0.01 Perceived sports competence:* r* = 0.39,* p* < 0.01 Perceived fundamental movement competence:* r* = 0.26,* p* < 0.01 **Locomotor competence** Perceived locomotor competence:* r* = 0.09,* p* > 0.05 **Object control competence** Perceived object control competence:* r* = 0.38,* p* < 0.01
Ryan et al. [82]	Cross-sectional	**Sample** 130 professional male youth soccer players **Mean age** 13.8 ± 2.9 years **Mean height** 167.9 ± 13.3 cm **Mean body mass** 57.3 ± 15.1 kg	**Overall competence** Functional Movement Screen™ [129, 130]	Process		**Muscular power** Countermovement jump **Speed** 0–10 m sprint		**Overall competence** Muscular power:* r* = 0.40, 95% CI = 0.25, 0.54 Speed:* r* = 0.32, 95% CI − 0.47, − 0.16
Smith et al. [119]	8-Month randomised controlled trial	**Sample** 361 Australian boys (intervention = 181; control = 180) **Mean age** Total = 12.7 ± 0.5 years Intervention = 12.7 ± 0.5 years Control: 12.7 ± 0.5 years	**Overall competence** RTSB [66]	Process	**Moderate-vigorous physical activity** Accelerometers (Actigraph GT3X +)	**Composite fitness score** 90-degree push-up test and Handgrip dynamometer [247, 248] **Weight status** Body fat % [175]		**Overall competence** MVPA: *r* = 0.16, *p* = 0.01 Composite fitness: *r* = 0.43, *p* < 0.01 Body fat %: *r* = − 0.28, *p* < 0.01
Smith et al. [83]^a^	Cross-sectional sample from Resistance Training for Teens randomised controlled trial intervention	**Sample** 548 adolescents (male *n* = 276; female *n* = 272) **Mean age** 14.1 ± 0.5 years	**Overall competence** RTSB [66]	Process		**Composite fitness score** Summed standardised scores from 90 degree push-up test and standing long jump **Weight status** BMI	**Self-efficacy for resistance training** Self-reported questionnaire [175] **Perceived strength** International Fitness Scale (IFS) [164] **Autonomous motivation for resistance training** Behavioural Regulations in Exercise Questionnaire Version 2 (BREQ-2) [154]	**Overall competence (male)** Composite fitness score: *β* = 0.33, SE = 0.05, 95% CI = 0.23, 0.42,* p* < 0.01 Weight status: *β* = − 0.06, SE = 0.05, 95% CI − 0.16, 0.03,* p* > 0.05 Self-efficacy: *β* = 0.21, SE = 0.05, 95% CI = 0.12, 0.31,* p* < 0.01 Perceived strength: *β* = 0.09, SE = 0.05, 95% CI − 0.01, 0.19,* p* > 0.05 Autonomous motivation: *β* = 0.24, SE = 0.05, 95% CI = 0.14, 0.34,* p* < 0.01 **Overall competence (female)** Composite fitness score: *β* = 0.46, SE = 0.05, 95% CI = 0.35, 0.56,* p* < 0.01 Weight status: *β* = − 0.29, SE = 0.05, 95% CI − 0.40, − 0.19,* p* < 0.01 Self-efficacy: *β* = 0.22, SE = 0.05, 95% CI = 0.12, 0.32,* p* < 0.01 Perceived strength: *β* = 0.22, SE = 0.05, 95% CI = 0.12, 0.31,* p* < 0.01 Autonomous motivation: *β* = 0.18, SE = 0.05, 95% CI = 0.08, 0.28,* p* < 0.01
Sommerfield et al. [120]	Cross-sectional	**Sample** 104 adolescent girls **Mean age** 14.0 ± 0.6 years **Mean height** 162.6 ± 5.9 cm **Mean body mass** 57.3 ± 9.7 kg	**Overall competence** Back squat assessment [146]	Process		**Strength** IMTP (absolute and relative) **Muscular power** Countermovement jump (unilateral and bilateral), drop vertical jump **Speed** 10-m and 20-m sprint		Only reported at associations between motor competence and strength **Overall competence** IMTP peak force: *r* = − 0.31, *p* < 0.01 IMTP relative peak force: *r* = − 0.42, *p* < 0.01
Tadiotto et al. [56]	Cross-sectional	**Sample** 62 adolescents (male *n* = 32; female *n* = 30) **Mean age (based on sit to stand time)** Fast group = 15.0 ± 1.7 years; intermediate group = 14.5 ± 1.9 years; slow group = 13.0 ± 2.1 years **Mean height (based on sit to stand time)** Fast group = 166.5 ± 8.0 cm; intermediate group = 161.6 ± 9.0 cm; slow group = 157.0 ± 7.6 cm **Mean body mass (based on sit to stand time)** Fast group = 65.0 ± 9.1 kg; intermediate group = 73.1 ± 16.0 kg; slow group = 73.9 ± 14.1 kg	**Overall competence** Supine to stand test [137]	Combined with scores separated for process and product measurements		**Weight status** BMI, fat mass, fat-free mass **Muscular endurance** Abdominal push-ups **Muscular strength** Handgrip strength **Cardiovascular endurance** Maximum incremental treadmill test (absolute and relative to body mass) **Flexibility** Sit and reach		**Overall competence (product)** Weight status (BMI):* r* = 0.59,* p* < 0.01 Weight status (fat mass):* r* = 0.69,* p* < 0.01 Weight status (fat free mass):* r* = − 0.12,* p* > 0.05 Muscular endurance:* r* = − 0.49,* p* < 0.01 Muscular strength (hand-grip right):* r* = − 0.42,* p* < 0.01 Muscular strength (hand-grip left):* r* = − 0.40,* p* < 0.01 Cardiovascular endurance (absolute):* r* = − 0.23,* p* > 0.05 Cardiovascular endurance (relative to body mass):* r* = − 0.66,* p* < 0.01 Flexibility:* r* = − 0.28,* p* < 0.05 **Overall competence (process)** Weight status (BMI):* r* = − 0.60,* p* < 0.01 Weight status (fat mass):* r* = − 0.67,* p* < 0.01 Weight status (fat-free mass):* r* = 0.21,* p* > 0.05 Muscular endurance:* r* = 0.39,* p* < 0.01 Muscular strength (handgrip right):* r* = 0.31,* p* < 0.01 Muscular strength (handgrip left):* r* = 0.32,* p* < 0.05 Cardiovascular endurance (absolute):* r* = 0.15,* p* > 0.05 Cardiovascular endurance (relative):* r* = 0.64,* p* < 0.01 Flexibility:* r* = 0.15,* p* > 0.05
Vedul-Kjelsas et al. [90]	Cross-sectional	**Sample** 67 adolescents (female *n* = 28; male *n* = 39) **Mean age** 11.46 ± 0.27 years **Mean height** 148.33 ± 7.15 cm **Mean body mass** 40.18 ± 7.79 kg	**Overall competence** MABC [249]	Product		**Composite fitness score** Test of physical fitness [217, 250]	**Perceived motor competence** Norwegian version of Harter’s Self-Perception Profile for Children [161, 167]	**Overall competence (whole sample)** Composite fitness score:* r* = − 0.61,* p* < 0.01 Perceived athletic competence:* r* = − 0.35,* p* < 0.01 **Overall competence (female)** Composite fitness score:* r* = − 0.57,* p* < 0.01 Perceived athletic competence:* r* = − 0.41,* p* < 0.05 **Overall competence (male)** Composite fitness score:* r* = − 0.70,* p* < 0.01 Perceived athletic competence:* r* = − 0.31,* p* > 0.05
Woods et al. [78]^c^	Cross-sectional	**Sample** 44 adolescent Australian football players **Age rang**e 17.1–18.1 years **Mean height** 186.7 ± 7.7 cm **Mean body mass** 78.8 ± 9.2 kg	**Individual motor competence elements** Modified Athletic Ability Assessment [135]: overhead squat, double lunge (left and right leg), single-leg Romanian deadlift (left and right leg) and push-up	Process		**Muscular power** Vertical jump; dynamic vertical jump (left and right leg take off) **Speed** 20-m sprint **Agility** AFL agility test **Cardiovascular endurance** Multi-stage fitness test		**Overhead squat** Muscular power: vertical jump:* r* = 0.32,* p* < 0.05 Muscular power: dynamic vertical jump (left leg):* r* = 0.40,* p* < 0.05 Muscular power: dynamic vertical jump (right leg):* r* = 0.30,* p* > 0.05 Speed:* r* = − 0.35,* p* < 0.05 Agility:* r* = − 0.26,* p* > 0.05 Cardiovascular endurance:* r* = 0.06,* p* > 0.05 **Double lunge (left leg)** Muscular power: vertical jump:* r* = 0.44,* p* < 0.05 Muscular power: dynamic vertical jump (left leg):* r* = 0.42,* p* < 0.05 Muscular power: dynamic vertical jump (right leg):* r* = 0.27,* p* > 0.05 Speed:* r* = − 0.41,* p* < 0.05 Agility:* r* = − 0.25,* p* < 0.05 Cardiovascular endurance:* r* = 0.37,* p* < 0.05 **Double lunge (right leg)** Muscular power: vertical jump:* r* = 0.40,* p* < 0.05 Muscular power: dynamic vertical jump (left leg):* r* = 0.38,* p* < 0.05 Muscular power: dynamic vertical jump (right leg):* r* = 0.25,* p* > 0.05 Speed:* r* = − 0.34,* p* < 0.05 Agility:* r* = − 0.20,* p* > 0.05 Cardiovascular endurance:* r* = 0.30,* p* < 0.05 **Push-up** Muscular power: vertical jump:* r* = 0.28,* p* > 0.05 Muscular power: dynamic vertical jump (left leg):* r* = 0.24,* p* > 0.05 Muscular power: dynamic vertical jump (right leg):* r* = − 0.13,* p* > 0.05 Speed:* r* = − 0.06,* p* > 0.05 Agility:* r* = − 0.02,* p* > 0.05 Cardiovascular endurance:* r* = − 0.07,* p* > 0.05 **Single leg Romanian deadlift (left leg)** Muscular power: vertical jump:* r* = 0.32,* p* < 0.05 Muscular power: dynamic vertical jump (left leg):* r* = 0.23,* p* > 0.05 Muscular power: dynamic vertical jump (right leg):* r* = − 0.07,* p* > 0.05 Speed:* r* = − 0.11,* p* > 0.05 Agility:* r* = − 0.17,* p* > 0.05 Cardiovascular endurance:* r* = 0.13,* p* > 0.05 **Single leg Romanian deadlift (right leg)** Muscular power: vertical jump:* r* = 0.26,* p* > 0.05 Muscular power: dynamic vertical jump (left leg):* r* = 0.19,* p* > 0.05 Muscular power: dynamic vertical jump (right leg):* r* = − 0.01,* p* > 0.05 Speed:* r* = − 0.04,* p* > 0.05 Agility:* r* = − 0.16,* p* > 0.05 Cardiovascular endurance:* r* = 0.12,* p* > 0.05

*β* beta coefficient, *BMI* body mass index, *CI* confidence interval, *r* correlation coefficient, *SE* standard error

^a^Not eligible for the meta-analysis because of missing data

^b^Missing data obtained via contacting the author

^c^Eligible for the meta-analysis but not included because of not fitting the groupings of motor competence (i.e. locomotor, object control, stability/balance, overall competence)

Forty-nine studies [36, 55, 57, 58, 66–68, 70–73, 75–77, 79, 81, 83–85, 87–91, 93–96, 98–100, 102–114, 116, 117, 119–121] recruited their participants from schools (e.g. high school, middle school students/athletes), nine studies [69, 78, 80, 82, 86, 92, 97, 101, 118] consisted of sports-based samples (e.g. amateur male basketball, academy male youth soccer), and three studies [56, 74, 115] described their participants as “adolescents”. Forty-seven studies included both male and female participants, eight studies consisted of male individuals only [72, 78–80, 82, 97, 118, 119], while four studies consisted of female individuals only [89, 101, 106, 120]. Two studies [69, 92] failed to report the sex characteristics of their samples. Forty-four studies reported the mean age of their participants (overall mean age = 13.59 ± 1.4 years; range = 11.26–16.40 years), while seven studies [55, 68, 78, 87, 88, 106, 112] reported the age range, and ten studies [56, 58, 71, 73, 96, 99, 100, 105, 117, 118] reported the mean age by various sub-groups (e.g. normal weight, overweight/obese groups).

Eight studies measured the maturity status of their participants. Seven studies [56, 72, 80, 97, 100, 118, 120] used the Mirwald equation [122], whilst one study [82] used the Khamis and Roche method [123]. Authors used maturity status to: (1) compare their participants’ maturity status based on motor competence and/or physical fitness scores [80, 82, 120]; (2) identify associations between maturity status and motor competence, physical and/or psychosocial characteristics (e.g. the correlation between maturity status and motor competence) [72, 97, 100]; (3) highlight that different subgroups were of the same age and maturity status [56]; or (4) identify the influence of motor competence and maturity status on physical fitness outcomes [118]. When assessing correlations between motor competence and physical activity, physical fitness and psychosocial characteristics, 34 studies [56, 57, 66, 68–73, 76, 78, 80–82, 85–87, 89, 91, 92, 96–98, 101, 105–109, 111, 112, 118, 120] assessed motor competence against one characteristic, 24 studies [36, 55, 58, 67, 74, 75, 77, 79, 83, 84, 90, 93–95, 99, 100, 102, 103, 110, 113, 114, 116, 117, 119, 121] against two characteristics, and three studies [88, 104, 115] against all characteristics.

Of the 61 studies within this review, 25 studies [66, 67, 70–73, 75, 76, 78, 82, 83, 89, 91, 93, 94, 100–102, 104, 110, 117–121] used a process-orientated motor competence assessment, and 31 studies [36, 55, 57, 58, 68, 69, 79–81, 84, 85, 87, 88, 90, 95–99, 103, 105–109, 111–116] used a product-orientated assessment. Only one study [74] used a combined process and product assessment of motor competence, while four studies [56, 77, 86, 92] used a combined motor competence assessment but reported process and product scores separately. Across the included studies, the following 27 motor competence measures were used: the Körperkoordinationstest Für Kinder ([124], *n* = 11 [36, 68, 79, 95, 96, 98, 103, 105–107, 116]); a combination of individual measures (e.g. Figure 8 dribble test, the leaping test; *n* = 10 [55, 58, 85, 87, 99, 111–115]); the resistance training skills battery ([66], *n* = 4 [66, 72, 83, 119]); the Victorian FMS manual [125] (*n* = 3 [71, 73, 89]); a combination of measures from the test of gross motor development (TGMD [126]), TGMD-2 [127] and the Victorian FMS manual [125] (*n* = 4 [70, 75, 102, 117]); an adapted version of the Körperkoordinationstest Für Kinder [124] (*n* = 2 [80, 88]); the Bruininks-Oseretsky Test of Motor Proficiency-2 Short Form (BOT-2 Short; [128]; *n* = 2 [84, 97]); a combination of the functional movement screen™ [129, 130] and the Y-balance tests [131] (*n* = 2 [86, 92]); the PE Metrics Battery [132] (*n* = 2 [93, 94]); the Functional Movement screen™ (*n* = 3 [82, 101, 118]); an adapted version of the Get Skilled Get Active Battery [133] (*n* = 1 [67]); a combination of the TGMD, TGMD-2, Victorian FMS manual, and the Functional Movement Screen™ (*n* = 1 [76]); the McCarron Assessment of Neuromuscular Development [134] (*n* = 1 [74]); the TGMD (*n* = 1 [77]); an adapted version of the Athletic Ability Assessment [135] (*n* = 1 [78]); the Movement Assessment Battery for Children-2 (MABC-2; [136]; *n* = 2 [81, 108]); the supine to stand test [137] (*n* = 1 [56]), a combination of the MABC-2 and the test of motor competence [138] (*n* = 1 [57]); a combination of the TGMD, TGMD-2 and Get Skilled Get Active tests (*n* = 1 [91]); the Athletic Introductory Movement screen ([139]) and tuck jump assessment [140] (*n* = 1 [100]); the MABC (*n* = 1 [90]); the TGMD-3 [141] (*n* = 1 [121]); a combination of the TGMD-3 and the Victorian FMS Manual [125] (*n* = 1 [104]); the Motorische Basiskompetenzen (MOBAK) [142–144] (*n* = 1 [109]); the Life-Long Physical Activity Skills Battery [145] (*n* = 1 [110]); the back squat assessment [146] (*n* = 1 [120]); and an unreferenced measure of stability/balance (*n* = 1 [69]).

A total of 30 studies measured the association between motor competence and physical activity among adolescents [36, 55, 58, 67, 70, 71, 74, 77, 79, 84, 87, 88, 91, 94, 95, 103–107, 110–117, 119, 121]. Measures of physical activity engagement included non-referenced self-reporting questionnaires (*n* = 4 [55, 71, 87, 88]), accelerometery (*n* = 9 [75, 91, 94, 104, 110, 113, 116, 117, 119]), the Physical Activity Questionnaire for Older Children (PAQ-C; [147]; *n* = 7 [79, 95, 103, 105–107, 111]), the Adolescent Physical Activity Recall Questionnaire [148] (*n* = 1 [67]), the Flemish Physical Activity Questionnaire [149] (*n* = 1 [36]), pedometers (*n* = 2 [74, 121]), step activity monitors (*n* = 1 [84]), the Leisure Time Physical Activity Questionnaire [150] (*n* = 1 [58]), the Health Behaviour in School-Aged Children Survey [151] (*n* = 1 [112]); the International Physical Activity Questionnaire (Short Form) [152] (*n* = 1 [114]); the MoMo Physical Activity Questionnaire [153] (*n* = 1 [115]); and an unreferenced question about weekly engagement in sport, fitness or recreational activity (*n* = 1 [77]).

The association between motor competence and physical fitness was assessed across ten domains (Table 2). Motor competence was assessed against composite fitness scores (*n* = 9 [57, 66, 83, 90, 97, 104, 108, 114, 119]), weight status (*n* = 21 [56, 58, 69, 74, 77, 79, 83, 84, 91, 93–96, 100, 101, 103, 105–107, 110, 116]), muscular endurance (*n* = 10 [56, 69, 74, 77, 84, 93, 94, 99, 110, 113]), muscular power (*n* = 12 [69, 72, 78, 82, 86, 88, 92, 100, 110, 115, 118, 120]), speed (*n* = 5 [69, 72, 78, 82, 120]), agility (*n* = 5 [72, 74, 86, 92, 118]), muscular strength (*n* = 6 [56, 72, 74, 88, 115, 120]), cardiovascular endurance (*n* = 16; [56, 72–74, 77, 78, 80, 84, 88, 93, 94, 99, 110, 113, 115, 117]), flexibility (*n* = 6 [56, 74, 77, 88, 110, 115]) and functional mobility (i.e., timed up-and-down stairs test; *n* = 1 [84]).

A total of five psychosocial domains were assessed against motor competence among adolescents. The association between motor competence and motivation was evaluated by six studies [36, 55, 83, 85, 98, 100]. Studies measured motivation using the Behavioural Regulation in Exercise Questionnaire-2 (BREQ-2; [154]; *n* = 2 [83, 100]), the Sport Motivation Scale [155] (*n* = 2 [55, 85]), a Dutch version of the Behavioural Regulation in Physical Education Questionnaire [156] (*n* = 1 [36]) and a Spanish version of the Perceived Locus of Causality Scale (PLOC; [157]; *n* = 1 [98]).

Seventeen studies measured the association between motor competence and perceived motor competence [36, 55, 67, 68, 81, 83, 85, 88–90, 98–100, 104, 109, 115, 121]. Measures utilised to assess perceived motor competence included the Physical Self-Perception Profile (PSPP [158, 159]; *n* = 1; [67]), the PSPP Sports Competence Subscale (*n* = 3 [55, 85, 99]), the PSPP and the Pictorial Scale of Perceived Movement Skill Competence (PSPMSC [160]; *n* = 1 [89]), the PSPMSC (*n* = 1 [98]), the PSPMSC and the PSPMSC in Stability Skills [68] (*n* = 1 [68]), the Sport/Athletic Competence Subscale [161] of the Children and Youth Physical Self Perception Profile [162] (*n* = 1 [36]), the Self-Description Questionnaire-2 [163] (*n* = 1 [81]), the International Fitness Scale [164] (*n* = 1 [83]), the Norwegian version [165] of the Perceived Athletic Competence Subscale of the Self-Perception Profile for Adolescents [166] (*n* = 1 [88]), the Norwegian version [167] of the Self-Perception Profile for Children [161] (*n* = 1 [90]), the Perceived Physical Ability Scale for Children [168] (*n* = 1 [100]), the Self-Perception Profile for Adolescents [169] (*n* = 1 [104]), the Perceived Competence Scale for Children [170] (*n* = 1 [121]), the Selbstwahrnehmung der motorischen Kompetenz (SEMOK) [109] (*n* = 1 [109]), and the Physical Self-Description Questionnaire [171, 172] (*n* = 1 [115]). Pullen et al. [100] also analysed the association between motor competence and global self-esteem via the Rosenberg Self-Esteem Scale [173]. Fu and Burns [121] also measured the association between motor competence and physical activity enjoyment via the Sport Enjoyment Scale [174].

Five studies [70, 75, 76, 83, 102] measured the association between motor competence and self-efficacy/confidence. Smith et al. [83] used a self-efficacy scale related to resistance training [175], Fu and Burns [121] used a six-item self-efficacy scale [176] and the remaining studies [70, 75, 76, 102] used the Physical Self-Confidence Scale [177].

### Risk of Bias Overview

The risk of bias overview of included studies is presented in Table 3. No studies met all six criteria, nine studies met five criteria [36, 57, 67, 73, 81, 97, 99, 102, 113], eight studies met four criteria [66, 68, 70, 76, 79, 90, 112, 117], 12 studies met three criteria [71, 74, 75, 82, 89, 92, 95, 100, 106, 108, 120, 121], 13 studies met two criteria [58, 72, 80, 83–85, 93, 94, 96, 98, 105, 114, 115] and 19 studies met one [55, 77, 78, 86, 87, 91, 101, 103, 104, 110, 111, 118, 119] or none [56, 69, 88, 107, 109, 116] of the criteria. Criteria one and four were the least met criteria (*n* = 15/61 and 17/61 respectively), followed by criterion five (*n* = 21/61), criterion six (*n* = 29/61) and criterion two (*n* = 30/61). Most studies met criterion three (40/61).

**Table 3:** Risk of bias assessment overview

Reference	Item 1	Item 2	Item 3	Item 4	Item 5	Item 6
Barnett et al. [67]	✓	✓	✓	✓	✓	✕
Britton et al. [104]	?	?	?	?	?	✓
Chagas and Batista [106]	?	✓	✓	?	?	✓
Chagas and Batista [107]	?	?	?	?	?	✕
Chagas and Batista [79]	✓	✓	✓	?	?	✓
Chagas and Batista [95]	✓	?	✓	?	?	✓
Chagas and Marinho [103]	?	✓	✕	?	?	?
Chagas et al. [96]	?	✓	?	?	?	✓
Chagas et al. [105]	?	✓	✓	?	?	?
Chang et al. [92]	?	?	✓	?	✓	✓
Chen and Housner [77]	?	?	?	?	?	✓
De Meester et al. [36]	✓	✓	✓	✓	✓	?
Deprez et al. [80]	?	✓	✓	?	?	✕
Estevan et al. [98]	✕	✓	✕	?	✓	?
Estevan et al. [68]	✕	✓	✓	✓	✓	✕
Fu and Burns [121]	✕	✓	✓	?	?	✓
Gísladóttir et al. [108]	?	✓	✓	?	?	✓
Gísladóttir et al. [57]	✓	✓	✓	?	✓	✓
Gu et al. [93]	?	✓	✓	?	?	✕
Gu et al. [94]	?	✓	✓	?	?	?
Hands et al. [74]	✓	✓	✓	?	?	✕
Haugen et al. [88]	?	?	?	?	?	?
Herrmann and Seelig [109]	?	?	?	?	?	?
Huhtiniemi et al. [99]	?	✓	✓	✓	✓	✓
Hulteen et al. [110]	?	?	?	?	?	✓
Huotari et al. [58]	✓	?	✓	?	?	?
Jaakkola et al. [87]	✕	?	✕	?	?	✓
Jaakkola and Washington [112]	?	?	✓	✓	✓	✓
Jaakkola et al. [114]	✕	✓	✓	?	?	✕
Jaakkola et al. [111]	?	?	✓	?	?	✕
Jaakkola et al. [113]	?	✓	✓	✓	✓	✓
Jekauc et al. [115]	?	?	✓	?	✕	✓
Kalaja et al. [55]	?	?	✕	✓	✕	?
Kalaja et al. [85]	✕	?	✕	✓	✓	?
Kokstejn et al. [97]	✓	✓	✓	✓	?	✓
Kovac et al. [101]	?	✕	✕	✕	✕	✓
Kramer et al. [86]	?	?	?	?	?	✓
Lloyd et al. [118]	?	?	?	?	?	✓
Lopes et al. [116]	?	?	?	?	?	✕
Lubans et al. [66]	✓	✓	✓	✓	?	?
McGrane et al. [70]	?	✓	✓	✓	✓	✕
McGrane et al. [75]	?	✓	✓	?	?	✓
McGrane et al. [117]	✓	✓	✓	?	?	✓
Nikolaos [69]	?	?	✕	?	✕	?
Nunez-Gaunaurd et al. [84]	✕	?	✕	✓	✓	?
O’Brien et al. [91]	?	?	✓	?	?	✕
Okely et al. [73]	✓	✓	✓	✓	✓	?
Okely et al. [71]	✓	✓	✓	?	?	?
Philpott et al. [76]	?	✓	✓	✓	✓	?
Philpott et al. [102]	✓	✓	?	✓	✓	✓
Pichardo et al. [72]	?	?	✓	?	✓	✕
Pullen et al. [100]	?	✕	✓	✕	✓	✓
Rigoli et al. [81]	✓	✓	✓	✓	✓	?
Rogers et al. [89]	✓	?	✓	?	✕	✓
Ryan et al. [82]	?	?	✓	?	✓	✓
Smith et al. [119]	?	?	✓	?	?	✕
Smith et al. [83]	?	✓	✓	?	?	?
Sommerfield et al. [120]	?	?	✓	?	✓	✓
Tadiotto et al. [56]	?	?	?	?	?	?
Vedul-Kjelsas et al. [90]	?	?	✓	✓	✓	✓
Woods et al. [78]	?	?	✕	?	?	✓

✓ indicates a low risk of bias, ✕ indicates a high risk of bias, ? indicates an inadequate or unclear description

Item 1 = Does the study adequately describe participant sampling procedures and inclusion criteria?

Item 2 = Does the study clearly outline the motor competence assessment(s) used (specific measures/procedures/valid)?

Item 3 = Does the study provide acceptable reliability information for the motor competence assessment(s) used?

Item 4 = Does the study clearly outline the physical activity/physical fitness/psychosocial assessment(s) used (specific measures/procedures/valid)?

Item 5 = Does the study provide acceptable reliability information for the physical activity/physical fitness/psychosocial assessment(s) used?

Item 6 = Of those who consented to the study, did an adequate proportion have complete data for the motor competence and the physical activity/physical fitness/psychosocial assessments?

### Meta-analysis

An overview of the associations between motor competence and physical activity, physical fitness and psychosocial characteristics in adolescence is presented in Fig. 3. Individual meta-analyses are presented in Figs. 4, 5, 6, 7, 8, 9, 10, 11, 12, 13, 14, 15 and 16.

#### Pooled Correlation Coefficients for Motor Competence and Physical Activity

For motor competence and physical activity, correlation coefficients were analysed from 13 studies [36, 55, 58, 67, 74, 79, 88, 91, 110, 111, 113, 119, 121]. Figure 4 shows the pooled correlation coefficients (i.e. overall summary statistics) were significant, positive and small (*r* = 0.20–0.26) for each type of motor competence evaluated against physical activity.

**Fig. 3:**
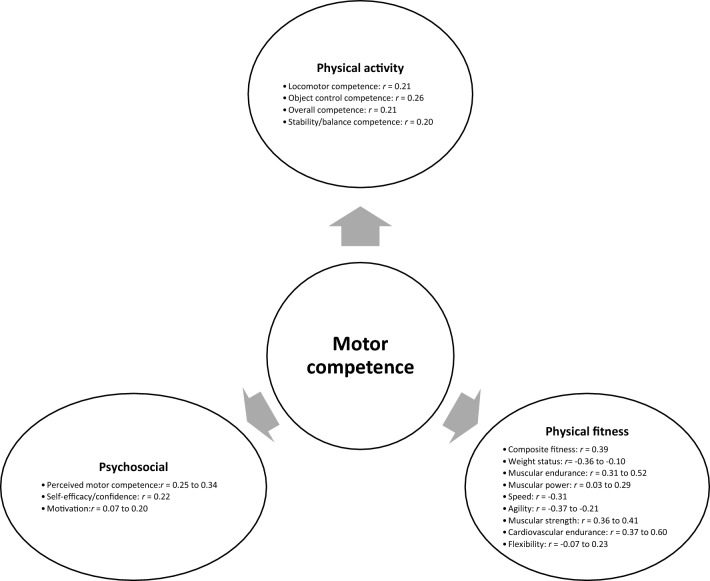
Overview of the range of pooled correlation coefficients between motor competence and physical activity, physical fitness and psychosocial characteristics in adolescents

**Fig. 4:**
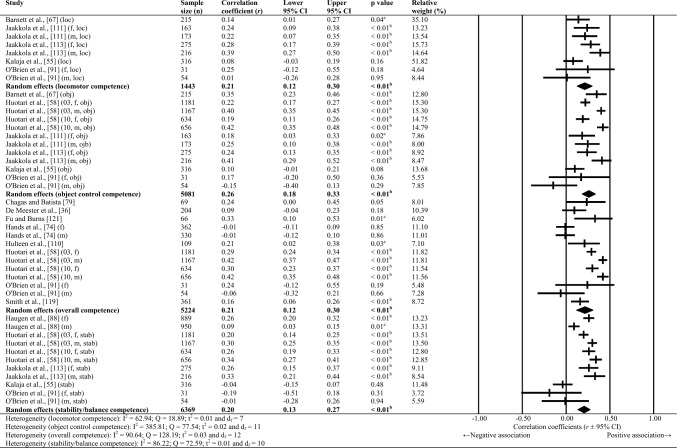
Forest plots showing the pooled correlation coefficients between motor competence and physical activity (*r* ± 95% confidence interval [CI]). Bold font indicates the summary statistics for each type of motor competence represented. *03* 2003 participants, *10* 2010 participants, *f* female, *loc* locomotor competence, *m* male, *obj* object control competence, *r* correlation coefficient, *stab* stability/balance competence, ^a^*p* < 0.05, ^b^*p* < 0.01

##### Pooled Correlation Coefficients for Motor Competence and Physical Fitness Characteristics

**Fig. 5:**
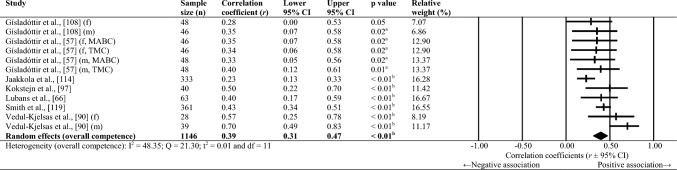
Forest plots showing the pooled correlation coefficients between motor competence and composite fitness scores (r ± 95% confidence interval [CI]). Bold font indicates the summary statistics for each type of motor competence represented. *f* female, *m* male, *MABC* movement assessment battery for children, *r* correlation coefficient, *TMC* test of motor competence, ^a^*p* < 0.05, ^b^*p* < 0.01

*Composite Fitness Scores* Seven studies analysed correlation coefficients for the association between motor competence and composite fitness [57, 66, 90, 97, 108, 114, 119]. Figure 5 shows that studies only reported correlation coefficients for overall competence, with the pooled correlation coefficient being significant, positive and moderate (*r* = 0.39).

**Fig. 6:**
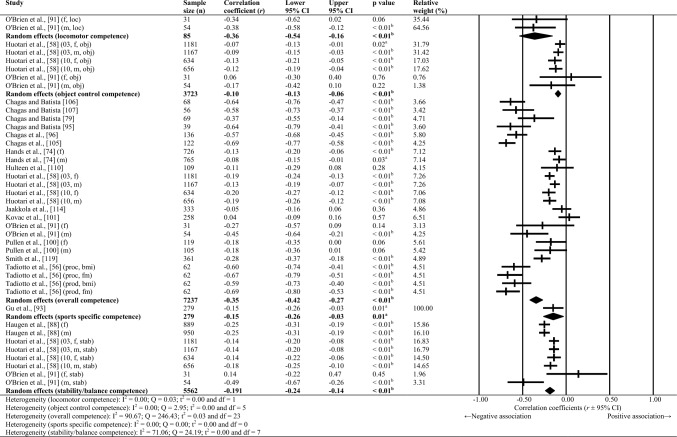
Forest plots showing the pooled correlation coefficients between motor competence and weight status (r ± 95% confidence interval [CI]). Bold font indicates the summary statistics for each type of motor competence represented. *03* 2003 participants, *10* 2010 participants, *bmi* body mass index, *f* female, *fm* fat mass, *loc* locomotor competence, *m* male, *obj* object control competence, *proc* process measure of motor competence, *prod* product measure of motor competence, *r* correlation coefficient, *stab* stability/balance competence, ^a^*p* < 0.05, ^b^*p* < 0.01

*Weight Status* The association between motor competence and weight status was analysed from 17 studies [56, 58, 74, 79, 88, 91, 93, 95, 96, 100, 101, 105–107, 110, 114, 119]. Pooled correlation coefficients ranged from trivial to moderate (*r* =  − 0.35 to − 0.10) and were all significant. The pooled correlation coefficients for locomotor and sports-specific competence consisted of fewer than three study samples (Fig. 6).

**Fig. 7:**
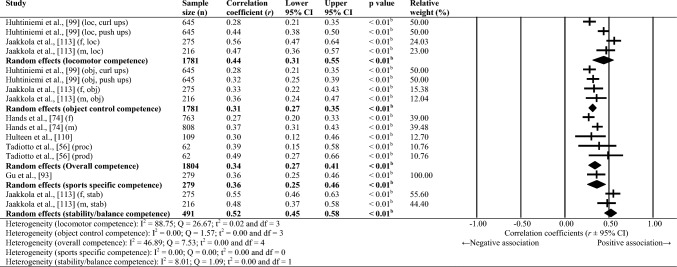
Forest plots showing the pooled correlation coefficients between motor competence and muscular endurance (*r* ± 95% confidence interval [CI]). Bold font indicates the summary statistics for each type of motor competence represented. *loc* locomotor competence, *obj* object control competence, *proc* process measure of motor competence, *prod* product measure of motor competence, *r* correlation coefficient, ^a^*p* < 0.05, ^b^*p* < 0.01

*Muscular Endurance* Six studies [56, 74, 93, 99, 110, 113] examined the association between motor competence and muscular endurance (Fig. 7). The pooled correlation coefficient was significant, positive and moderate for overall competence (*r* = 0.34), locomotor competence (*r* = 0.44), object control competence (*r* = 0.31) and sports-specific competence (*r* = 0.36) to muscular endurance. Stability/balance competence had a significant, positive and high association with muscular endurance (*r* = 0.52). However, the pooled correlation coefficients for stability/balance competence and sports-specific competence to muscular endurance consisted of fewer than three study samples.

**Fig. 8:**
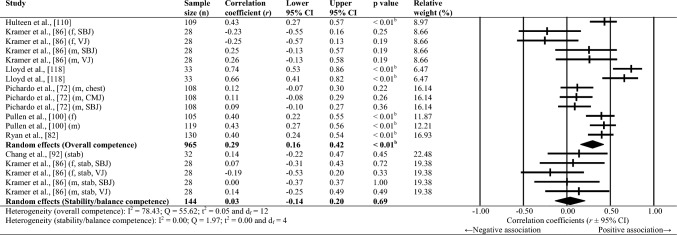
Forest plots showing the pooled correlation coefficients between motor competence and muscular power (*r* ± 95% confidence interval [CI]). Bold font indicates the summary statistics for each type of motor competence represented. *chest* throw, *f* female, *m* male, *r* correlation coefficient, *SBJ* standing broad jump, *stab* stability/balance competence, *VJ* vertical jump, ^a^*p* < 0.05, ^b^*p* < 0.01

*Muscular Power* The meta-analysis of the association between motor competence and muscular power evaluated the correlation coefficients from seven studies [72, 82, 86, 92, 100, 110, 118]. Figure 8 shows significant positive correlation coefficients for overall competence (*r* = 0.29, small) and stability/balance competence (*r* = 0.03, trivial) to muscular power.

**Fig. 9:**
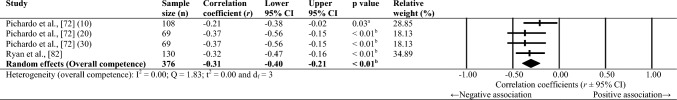
Forest plots showing the pooled correlation coefficients between motor competence and speed (*r* ± 95% confidence interval [CI]). Bold font indicates summary statistics for each type of motor competence represented, *10* 10-m sprint time, *20* 20-m sprint time, *30* 30-m sprint time, *r* = correlation coefficient, ^a^*p* < 0.05, ^b^*p* < 0.01

*Speed* Two studies [72, 82] were analysed for the association between motor competence and speed. A pooled correlation coefficient was produced for overall competence only, which was significant, negative and moderate (*r* =  − 0.31; Fig. 9).

**Fig. 10:**
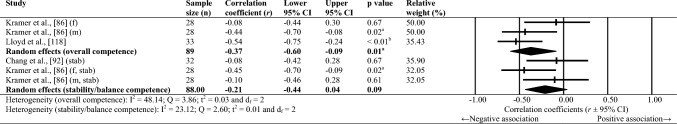
Forest plots showing the pooled correlation coefficients between motor competence and agility (*r* ± 95% confidence interval [CI]). Bold font indicates the summary statistics for each type of motor competence represented. *f* female, *m* male, *r* correlation coefficient, *stab* stability/balance competence. ^a^*p* < 0.05, ^b^*p* < 0.01

*Agility* The association between motor competence and agility was evaluated from three studies [86, 92, 118]. Figure 10 shows that pooled correlation coefficients for overall competence (*r* =  − 0.37, *p* = 0.01) and stability/balance (*r* =  − 0.21, *p* > 0.05) competence were negative, moderate and small, respectively.

**Fig. 11:**
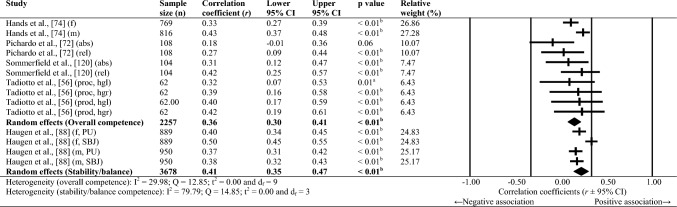
Forest plots showing the pooled correlation coefficients between motor competence and muscular strength (*r* ± 95% confidence interval [CI]). Bold font indicates the summary statistics for each type of motor competence represented. *abs* absolute strength, *f* female, *hgl* hand grip test left hand, *hgr* hand grip test right hand, *m* male, *proc* process measure of motor competence, *prod* product measure of motor competence, *PU* push-up test, *r* = correlation coefficient, *rel* strength relative to body mass, *SBJ* standing broad jump test, ^a^*p* < 0.05, ^b^*p* < 0.01

*Muscular Strength* A total of five studies [56, 72, 74, 88, 120] were evaluated for the association between motor competence and muscular strength. Pooled correlation coefficients were produced for overall competence (*r* = 0.36) and stability/balance competence (*r* = 0.41), which were significant, positive and moderate (Fig. 11).

**Fig. 12:**
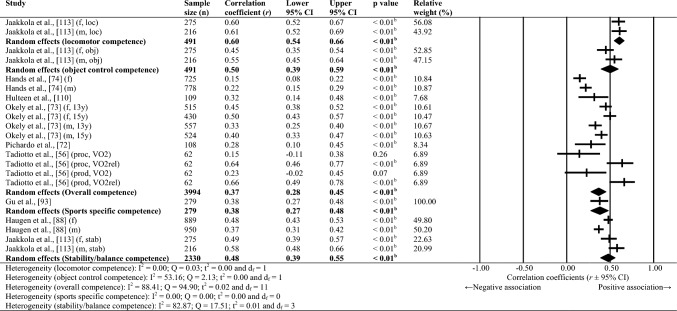
Forest plots showing the pooled correlation coefficients between motor competence and cardiovascular endurance (*r* ± 95% confidence interval [CI]). Bold font indicates the summary statistics for each type of motor competence represented. *13y* 13 years old, *15y* 15 years old, *f* female, *loc* locomotor competence, *m* male, *obj* object control competence, *stab* stability/balance competence, *proc* process measure of motor competence, *prod* product measure of motor competence, *r* correlation coefficient, *VO2* max, *VO2rel* VO” max relative to body mass, ^a^*p* < 0.05, ^b^*p* < 0.01

*Cardiovascular Endurance* The meta-analysis to evaluate the association between motor competence and cardiovascular endurance consisted of eight studies [56, 72–74, 88, 93, 110, 113]. Figure 12 shows the pooled correlation coefficients for each element of motor competence measured. The associations for all components with cardiovascular endurance were significant, positive and moderate (*r* = 0.37 to 0.48), except for locomotor (*r* = 0.60) and object control (*r* = 0.50) competence, which were significant, positive and high. However, the correlation coefficients for locomotor competence, object control competence and sports-specific competence consisted of fewer than three studies.

**Fig. 13:**
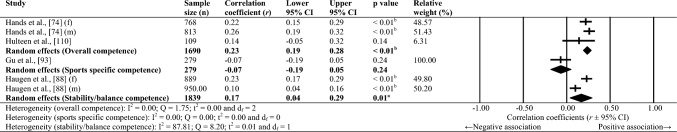
Forest plots showing the pooled correlation coefficients between motor competence and flexibility (*r* ± 95% confidence interval [CI]). Bold font indicates the summary statistics for each type of motor competence represented. *f* female, *m* male, *r* correlation coefficient, ^a^*p* < 0.05, ^b^*p* < 0.01

*Flexibility* A total of four studies [74, 88, 93, 110] were evaluated to identify the pooled correlation coefficients for motor competence and flexibility. Sports-specific competence had a non-significant negative trivial association with flexibility (*r* =  − 0.07), while significant positive small associations were identified for overall competence (*r* = 0.23) and stability/balance competence (*r* = 0.17) with flexibility. However, the meta-analyses for sports-specific competence and stability/balance competence consisted of fewer than three studies (Fig. 13).

##### Pooled Correlation Coefficients for Motor Competence and Psychosocial Characteristics

**Fig. 14:**
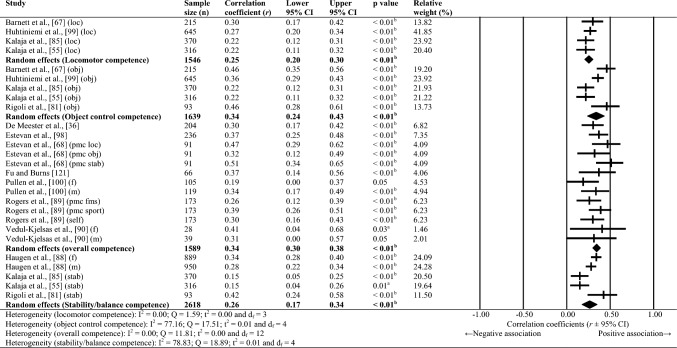
Forest plots showing the pooled correlation coefficients between motor competence and perceived motor competence (*r* ± 95% confidence interval [CI]). Bold font indicates the summary statistics for each type of motor competence represented.* f* female, *loc* locomotor competence, *m* male, *obj* object control competence, *pmc fms* perceived motor competence in fundamental movement skills, *pmc loc* perceived motor competence in locomotor skills, *pmc obj* perceived motor competence in object control skills, *pmc self* self-competence, *pmc sport* perceived motor competence in sports, *pmc stab* perceived motor competence in stability/balance skills, *r* correlation coefficient, *stab* stability/balance competence, ^a^*p* < 0.05, ^b^*p* < 0.01

*Perceived Motor Competence* For the association between motor competence and perceived motor competence, a total of 13 studies [36, 55, 67, 68, 81, 85, 88–90, 98–100, 121] were evaluated (Fig. 14). The associations between locomotor competence and stability/balance competence to perceived motor competence were significant, positive and small (*r* = 0.25 and 0.26, respectively). Further, significant positive moderate associations were identified for object control competence (*r* = 0.34) and overall competence (*r* = 0.34).

**Fig. 15:**
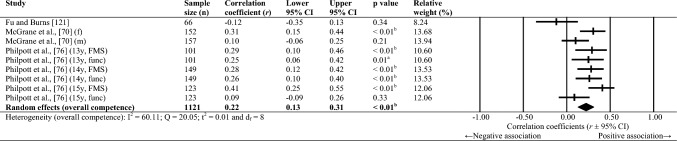
Forest plots showing the pooled correlation coefficients between motor competence and self-efficacy/confidence (*r* ± 95% confidence interval [CI]). Bold font indicates the summary statistics for each type of motor competence represented. *13y* 13-year-olds, *14y* 14-year-olds, *15y* 15-year-olds, *f* female, *FMS* fundamental movement skill assessment, *func* functional movement screen assessment, *m* male, *r* = correlation coefficient, ^a^*p* < 0.05, ^b^*p* < 0.01

*Self-Efficacy/Confidence* Three studies [70, 76, 121] evaluated the association between motor competence and self-efficacy/confidence (Fig. 15). The association between overall competence and self-efficacy/confidence was small (*r* = 0.22); no further elements of motor competence were represented.

**Fig. 16:**
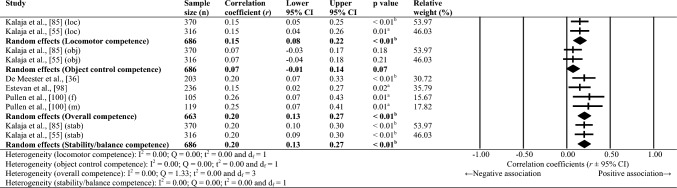
Forest plots showing the pooled correlation coefficients between motor competence and motivation (*r* ± 95% confidence interval [CI]). Bold font indicates the summary statistics for each type of motor competence represented. *f* female, *loc* locomotor competence, *m* male, *obj* object control competence, *r* correlation coefficient, *stab* stability/balance competence, ^a^*p* < 0.05, ^b^*p* < 0.01

*Motivation* A total of five studies [36, 55, 85, 98, 100] were analysed to identify the association between motor competence and motivation. The pooled correlations for all elements of motor competence were significant, except for object control competence, where the association was positive but trivial (*r* = 0.07). Associations for locomotor, overall and stability/balance competence were positive and small (*r* = 0.15 to 0.20). All elements of motor competence (except overall competence) were represented by fewer than three study samples (Fig. 16).

##### Heterogeneity

The degree of heterogeneity was moderate (> 50%) for locomotor competence to physical activity, stability/balance competence to weight status and object control competence to cardiovascular endurance. A high degree of heterogeneity (> 75%) was identified for overall, object control and stability/balance competence to physical activity, overall competence to weight status, locomotor competence to muscular endurance, overall competence to muscular power, stability/balance competence to muscular strength, overall and stability/balance competence to cardiovascular endurance, stability/balance competence to flexibility, and object control and stability/balance competence to perceived motor competence.

##### Sensitivity

The sensitivity analysis mainly showed minor changes. Independently eliminating three subgroup samples (male and female subgroup samples from the Huotari et al. 2010 cohort [58] and the male subgroup sample from O’Brien et al. [91]) altered the association between object control competence and weight status from small to trivial. The overall competence and muscular power association changed from small to moderate when individually removing each muscular power correlation from Pichardo et al. [72]; male and female vertical jump correlations from Kramer et al. [86]; and the female standing broad jump correlation from Kramer et al. [86]. The association between overall motor competence and speed increased from small to moderate when independently removing 20-m or 30-m sprint correlations from one study [72]. Removing Lloyd et al. [118] changed the association between overall competence and agility from moderate to small, while removing the female sample from Kramer et al. [86] changed the association between stability/balance competence and agility from non-significant and small to non-significant and trivial. The removal of the male sample from Haugen et al. [88] altered the association between stability/balance competence and cardiovascular endurance from moderate to high.

##### Evaluation of Small Study Effects

Inspection of the funnel plots and Egger’s regression intercepts revealed statistically significant Egger’s regression statistics for the association between overall competence and weight status (intercept =  − 4.21, 95% CI − 6.17, − 2.26, *p* < 0.01). The association between overall competence and weight status was not considered symmetrical, indicating the presence of a small study effect [178].

##### Moderator Variables

The subgroup analysis of the potential moderator variables (i.e., age, sex, type of motor competence assessment) is presented in supplementary Table 1. Pairwise comparisons showed three significant moderators; (1) the association between object control competence and physical activity was greater for male individuals (*r* = 0.33) compared with female individuals (*r* = 0.21, *p* = 0.04); (2) the association between overall competence and physical activity was greater in studies using product motor competence assessments (*r* = 0.31) versus process assessments (*r* = 0.18; *p* = 0.03); and (3) the association between overall competence and weight status was greater for studies with a mean age between 13 and 15 years (*r* =  − 0.37), compared with studies with a mean age between 11 and 12 years (*r* =  − 0.21; *p* = 0.03). There were no other significant differences in associations for motor competence and physical activity, physical fitness or psychosocial characteristics between any other potential moderators.

## Discussion

### Overview of the Main Findings

A key focus during adolescence is the synergistic development of motor competence, physical fitness and psychosocial characteristics [1]. The interaction between these characteristics is suggested to induce positive or negative physical activity and weight status trends amongst youth [21]; a hypothesis that potentially explains declining physical activity [8] and increasing obesity levels (e.g. UK [9], USA [10]) amongst these individuals. This systematic review with meta-analysis is the first to (1) analyse the scientific literature evaluating associations between motor competence and physical activity, physical fitness and/or psychosocial characteristics amongst adolescents; (2) evaluate the associations between motor competence and physical activity, physical fitness characteristics and/or psychosocial characteristics amongst adolescents; and (3) investigate the impact of moderator variables (i.e. age, sex, type of motor competence assessment) on these associations.

A total of 61 studies were reviewed [36, 55–58, 66–121], totalling 22,256 participants, providing a comprehensive systematic evidence base of the associations between motor competence and physical activity, physical fitness and psychosocial characteristics amongst adolescents. Findings from the qualitative review indicated that when examining the associations of motor competence during adolescence: (1) risk of bias is present across all studies; (2) longitudinal evaluations are limited, (3) few studies account for, or consider, maturity status, (4) few studies associate motor competence across multiple characteristics (i.e. physical activity, physical fitness, psychosocial) and (5) either process (i.e., technique) or product (i.e. outcome) measures are favoured when assessing motor competence compared to combined (process and product) methods.

Within the present study, physical activity, composite fitness, muscular endurance, muscular power, muscular strength, cardiovascular endurance, perceived motor competence, self-efficacy/confidence and motivation were positively associated with motor competence; weight status, speed and agility were inversely associated with motor competence. Flexibility showed positive and negative associations with motor competence depending upon the motor skills assessed. These findings align with previous evidence [12, 14, 21, 25, 28] across youth, suggesting that associations of motor competence continue throughout childhood and adolescence. Moderator comparisons (i.e. age, sex, type of motor competence assessment) presented three significant differences: (1) the association between object control competence and physical activity was greater for male individuals compared with female individuals; (2) the association between overall competence and physical activity was greater in studies using product motor competence assessments versus process assessments; and (3) the association between overall competence and weight status was greater for studies with a mean age between 13 and 15 years, compared with those between 11 and 12 years. These findings suggest that motor competence, physical activity engagement and physical fitness are complex during adolescence, when substantial physiological, biological and body composition changes are ongoing, meaning a greater understanding is required.

### Summary of Study Methods

Risk of bias was present across all included studies (0/61 met all six criteria). Validity (criterion two) and reliability (criterion three) of motor competence assessments had the highest adherence. Thus, while numerous motor competence assessments are available, the most current assessments are valid and reliable for practitioners to utilise within their environments. Sampling characteristics (criterion one) and validity of physical activity/fitness/psychosocial measures (criterion four) presented the lowest adherence. The low adherence to criterion one is attributed to the limited detail regarding sampling methods (e.g. random/convenience) and participant demographics (e.g. age, sex, ethnicity). Criterion four’s low adherence highlights inconsistencies in reporting the validity of measures used. These inconsistencies could confound the results presented and indicate that future research should utilise valid measures of physical activity, physical fitness and psychosocial characteristics during adolescence. Such information is important to fully understand the confounding factors that may influence any associations evaluated. Thus, authors should provide detail regarding participant sampling characteristics (e.g. sampling method, sample size, age, sex, stage of maturity) and the validity and reliability of study measures (e.g. of motor competence, and physical activity/fitness/psychosocial measures) to enhance study quality.

Most studies (45 out of 61) included within the systematic review used cross-sectional study designs, with ten studies [80, 103–105, 111–116] collecting longitudinal evaluations. The remaining studies conducted randomised controlled trial interventions [102, 117, 119], or used validity and reliability methods [66, 68, 110]. This finding aligns with previous motor competence reviews [14, 29, 47], and supports the need for future longitudinal investigations. Whilst cross-sectional study designs allow researchers to highlight current trends at single timepoints, longitudinal designs may be more appropriate to understand the developmental trajectories of the associations between these characteristics, alongside the long-term influence of potential moderators (e.g. sex, age, maturity status). Furthermore, longitudinal research can confirm previous cross-sectional outcomes and highlight the most appropriate opportunities for interventions to enhance health, well-being and performance outcomes in adolescence [179].

When evaluating motor competence, physical activity, physical fitness and psychosocial characteristics across adolescence, maturity status should be considered. Maturity status is asynchronous with chronological age [38, 39] and can lead to temporary reductions in motor competence (i.e. adolescent awkwardness) during the adolescent growth spurt [42]. Eight studies within this review measured the maturity status of adolescents. For example, Ryan et al. [82] showed that Fundamental Movement Screen™ scores stagnated between pre-PHV and circa-PHV (*d* = 0.3; 95% CI − 0.6, 1.2), before increasing during post-PHV (circa- to post-PHV *d* = 1.4; 95% CI 0.5, 2.2), which supports the adolescent awkwardness hypothesis during peak growth. Furthermore, Kokstejn et al. [97] showed that during pre-PHV (estimated years from PHV =  − 2.88 ± 0.3 years), adolescents’ motor competence is negatively associated with maturity status (*r* =  − 0.29, *p* < 0.01), whilst Pichardo et al. [72] identified no association between maturity status and motor competence in circa-PHV male individuals (estimated years from PHV = 0.2 ± 0.9 years; *r* = 0.00, *p* > 0.05). These findings show that stages of maturity may influence health and performance characteristics differently. While measuring maturity status is a strength of these studies, no studies explored the effect of maturity status on associations between motor competence and physical activity, physical fitness and psychosocial characteristics. Future research should longitudinally track maturity status during adolescence and examine its influences on the association between motor competence and physical activity, physical fitness and psychosocial characteristics.

The hypothesised Stodden et al. [21] model has been responsible for most motor competence research, worldwide, over the last decade. However, while multiple motor competence associations were hypothesised, most studies (*n* = 34) within this review only compared motor competence to one characteristic (i.e. physical activity, physical fitness or psychosocial). Only three studies [88, 104, 115] evaluated associations across all characteristics. This finding supports that of Barnett et al. [29] who identified that few studies have investigated the entire model. One explanation for this finding is that multivariate approaches may be required to analyse associations between the variables within the Stodden et al. [21] model (e.g. physical fitness, psychosocial) because a univariate analysis can only determine relationships between two variables in a pairwise manner at any given time [180]. Nevertheless, based on currently available evidence, only inferences can be made on all aspects of the Stodden et al. [21] model in adolescents, and there is a need for more holistic longitudinal research to examine the model in its entirety.

When measuring motor competence, most studies used process (i.e. technique; *n* = 25) or product (i.e. outcome; *n* = 31) assessments. Only one study used a combined motor competence measure (i.e. process and product criterion; [74]), while four studies [56, 77, 86, 92] reported separate correlations for process and product elements. These findings support other reviews (e.g. [181]), which similarly show studies favouring process or product assessments of motor competence. Such methods limit the overview of an individual’s motor competence [182]. For example, evaluating an individual’s technique enables assessors to identify and correct inadequate movement patterns to inform training interventions [183], prevent injury [184] and increase perceived motor competence [28]. Conversely, product-based measures show long-term changes in movement outcomes [185]. Process evaluations are subjective and require experienced assessors [186], while product-based measures cannot identify individual differences in motor competence as they are outcome based [187]. Consequently, authors have developed valid approaches to assess combined motor competence (e.g. the Canadian Agility Movement Skills Assessment [188], and the Dragon’s Challenge [189]), which offer viable alternatives that practitioners should consider for assessing motor competence.

### Summary of Meta-analyses

When assessing associations with physical activity, physical fitness and psychosocial characteristics, meta-analyses were conducted separately for different motor competence domains (i.e. overall competence, locomotor, object control, stability/balance, sports specific). This approach highlighted the scarcity of studies that provided correlations for the separate domains (see Figs. 3, 4, 5, 6, 7, 8, 9, 10, 11, 12, 13, 14, 15 and 16), meaning that for some characteristics, insufficient study samples were available to analyse their associations with motor competence. Therefore, care should be taken when reviewing some associations, owing to their limited evidence base. Where possible, future research should report associations with physical activity, physical fitness, and psychosocial characteristics as an overall score and separate motor competence domains.

#### Heterogeneity

The degree of heterogeneity varied depending on the characteristics measured. Higher heterogeneity occurred within meta-analyses consisting of greater study samples/sample sizes. Heterogeneity arises because of the grouping of studies that are methodologically diverse [64]. Thus, within the different meta-analyses, higher heterogeneity likely represents the diversity of the included studies’ population characteristics (e.g. sex, age, nationality) and the variety of motor competence assessments used across studies (27 different assessments identified). Thus, future research requires more consistent approaches for measuring associations between motor competence, physical activity, physical fitness and psychosocial characteristics among adolescents.

#### Association Between Motor Competence and Physical Activity

In the meta-analyses of 13 studies, a small association between motor competence and physical activity was seen among adolescents. The lowest association with physical activity was stability/balance competence, and the highest association with physical activity was overall competence, suggesting that a variety of motor skills such as throwing, catching, running, jumping and balancing are similarly important for physical activity engagement. A recent review indicated that supportive social environments are key to adolescent physical activity behaviours (e.g. active travel, sports participation) [190]. Perhaps, such environments may favour those with a broad range of motor skills that allow participation at the same levels as their peers (i.e. can engage successfully in a given social environment), particularly as displaying incompetence in front of others and exposure to embarrassment are perceived barriers to physical activity during adolescence [191]. Such experiences may be exaggerated in countries where there are strong links between school and sport (e.g. USA), although further research is required to test this hypothesis.

Current findings support previous reviews that identify positive associations between motor competence and physical activity in children and adolescents [25–28] but contradict the recent findings of Barnett et al., [29] who found no evidence supporting these associations. While Barnett et al. [29] explain their findings via a publication bias and a tendency within sports science research to only report significant associations, the present review shows no evidence of publication bias, with both non-significant and significant correlations extracted from the included studies. However, the present review’s sole focus on adolescent populations and the lack of longitudinal evidence presented may explain this contradiction.

Because of the variance in study methods (e.g. objective vs subjective physical activity assessments, participant characteristics, motor competence measures), comparing studies is challenging. Additionally, the tools used to assess physical activity and motor competence associations need acknowledging. For example, accelerometery does not capture the intensities of specific motor competencies (e.g. object control) [192], and consequently presents a lower association with these motor competencies [29]. This limitation is highlighted by O’Brien et al., [91], who measured physical activity in male individuals via accelerometery and reported a trivial association between physical activity and stability/balance competence. Measurement limitations should be considered during the research design process when assessing the associations between physical activity and motor competence in adolescents.

#### Association Between Motor Competence and Physical Fitness

Within these meta-analyses, various pathways of the Stodden et al. [21] model are represented. However, this study evaluated a broader range of physical fitness characteristics against motor competence compared with others (e.g. [12].), indicating the scarcity of evidence investigating individual physical fitness characteristics compared to other characteristics within the Stodden et al. [21] model. Thus, more research is required to strengthen the understanding of physical fitness and motor competence in adolescents.

*Composite Fitness* This review identified a moderate positive association between motor competence and composite fitness (*r* = 0.39). However, this association may represent similarities between product-based motor competence assessments and physical fitness measures (e.g. distance covered in standing long jump) that consist of similar neuromuscular actions [12]. For example, Vedul-Kjelsas et al. [90] measured physical fitness via a tennis ball throw, which bears similarities to components of the MABC-2 (e.g. ball skills). Within this meta-analysis, ten study samples utilised product-based assessments compared to two samples [66, 119] using process-based measurements. However, both samples identified a moderate positive association between process-orientated assessments and these characteristics, which suggests that associations may not be influenced by the type of motor competence assessment used. Future research should consider the similarities between product-based motor competence assessments and physical fitness measures in their methodologies. Adopting a combined (i.e. process and product) measure of motor competence is recommended when comparing to composite fitness scores, to account for measurement similarities and provide greater clarity on this particular association.

*Weight status* Weight status was negatively associated with motor competence in the meta-analyses (*r* =  − 0.36 to − 0.10). All motor competence domains were represented, although the meta-analysis for locomotor competence and sports-specific competence included insufficient study samples. These findings support similar evidence in youth [12, 25] and may be explained by the detrimental effect of increased body mass on motor competencies involving the projection of an individual’s body mass (e.g. jumping, running [12, 193]). However, body mass index was the most popular measure of weight status (*n* = 36/41 study samples). Measuring weight status via body mass index is a limitation of the current adolescent literature as lean/fat mass cannot be directly measured [84, 91, 194]. Within this review, Tadiotto et al. [56] identified that fat mass was negatively associated with motor competence, while lean mass was positively associated. This finding highlights the importance of differentiating between components of body composition when comparing associations with motor competence during adolescence, where lean mass gains occur, especially in male individuals [195]. Consequently, future research should focus on utilising more appropriate and practical measures of weight status that can differentiate between lean and fat mass (e.g. bioelectrical impedance) [28].

Of the meta-analyses undertaken, only the association for overall competence and weight status presented a small study effect, with the funnel plot indicating the presence of a significant publication bias. Explanations for the publication bias within this particular meta-analysis could include the use of a sedentary sample ([56] inclusion criteria = not physically active except for school time physical education and > 2 h of screen time per day), participants of a low socioeconomic status [96] and small sample sizes [95]. Therefore, care should be taken when interpreting the association between overall competence and weight status presented in this review, and future research should seek to limit publication bias.

*Muscular Endurance, Power and Strength* Compared with previous reviews (e.g. [12, 25].), this meta-analysis conducted a broader evaluation of motor competence associations with musculoskeletal fitness (e.g. muscular endurance, power, strength). The meta-analyses identified moderate positive associations between motor competence and musculoskeletal endurance, and muscular strength, as well as trivial-to-small positive associations with muscular power. Such findings suggest that musculoskeletal fitness and motor competence are mutually important for physical activity engagement [196]. For example, athletic tasks combine different skills that require both learnt levels of coordination and efficient force production/absorption capabilities (e.g. netball pass, jumping to catch a rebound in basketball) [12, 18]. Therefore, interventions should seek to synergistically develop musculoskeletal fitness and motor competence for positive health outcomes. Within this review, authors lacked consensus when classifying musculoskeletal fitness measures. For example, Kramer et al. [86] and Pichardo et al. [72] measured muscular power via a standing broad jump, whilst Haugen et al. [88] used this assessment to measure muscular strength. The limited consensus creates a cross-over in associations of motor competence and musculoskeletal fitness characteristics (i.e. muscular power scores contributing to muscular strength associations and vice versa), which could confound the associations presented. Therefore, future research requires more standardised measures to assess musculoskeletal fitness characteristics and facilitate between-study comparisons.

*Speed and Agility* Motor competence was negatively associated with speed and agility. No previous review has examined these associations because of focusing on health-related fitness (i.e. cardiovascular and musculoskeletal fitness; [12, 25, 29]). A broader focus on physical fitness components of athleticism [1] is a strength of the present study and allows the evaluation of additional characteristics required for physical activities/sports. These negative associations indicate that better speed and agility performance is synonymous with greater motor competence. However, readers should cautiously interpret the associations between motor competence, speed and agility because of the few studies (two for speed, three for agility) and study samples (four for speed, six for agility) evaluated. The need for caution is highlighted by a sensitivity analysis. Independently removing two study samples from the motor competence-speed meta-analysis changed this negative association from moderate to small, while the removal of one study from the agility meta-analysis changed this negative association from small to trivial. Nevertheless, low correlations between motor competence and speed/agility indicate the importance of other physical fitness characteristics for speed/agility. Previous research supports this hypothesis as relative strength is associated with longer step lengths (*r* = 0.79), and faster sprint speed (*r* = 0.42) [197]. Because of insufficient studies investigating the association between motor competence and speed, and agility, further research is required to understand these interactions fully.

*Cardiovascular Endurance* Overall, sports-specific and stability/balance competence were moderately associated with cardiovascular endurance (*r* = 0.38 to 0.60). However, a lack of study samples for locomotor competence (*n* = 2), object control competence (*n* = 2) and sports-specific competence (*n* = 1) means that these associations are inconclusive. Nevertheless, 12 study samples provide strong evidence for a moderate association between overall competence and cardiovascular endurance, which supports other findings across youth [12, 25, 28, 29]. Cattuzzo et al. [12] hypothesised that multiple physical fitness characteristics are both directly (i.e. via neuromuscular development) and indirectly (i.e. increased ability to participate in physical activities that promote cardiovascular fitness) linked with motor competence. For example, activities promoting cardiovascular endurance require repetitive, consecutive, concentric and eccentric contractions, which encompass contralateral limb coordination [12, 72]. These muscular actions may explain the high association between locomotor competence and cardiovascular endurance (*r* = 0.60) presented by two study samples within this meta-analysis. However, future study needs to explore this hypothesis owing to a lack of study samples for different motor competence domains.

*Flexibility* This meta-analysis shows that the association between motor competence and flexibility is inconclusive and concurrent with similar findings in youth [12, 25, 29]. The present results can be attributed to a lack of studies exploring this association. Nevertheless, both hyperflexibility and hypoflexibility can affect children’s movement capabilities [31]. Further, some adolescents experience temporary reductions in motor competence during circa-PHV [42], suggesting that maturation may affect flexibility. With limited consideration for maturity status throughout this review, further research is needed to clarify the association between motor competence and flexibility during adolescence.

#### Association Between Motor Competence and Psychosocial Characteristics

*Perceived Motor Competence and Confidence* The association between motor competence and perceived motor competence ranged from small to moderate, with all domains except sports-specific competence represented. The strongest evidence for this association was for overall competence (13 study samples included). Less evidence was available for locomotor, object control and stability/balance competence (four, five and five, respectively), suggesting that more in-depth evaluations of these associations are required. The present findings support those of De Meester et al. [14], who identified a small association between overall and perceived motor competence (*r* = 0.25). Previous understanding suggests that an individual’s accuracy of estimating motor competence increases with age [137]. However, because of insufficient study samples across different age groups (13 and 15 years [*n* = 7], followed by 11–12 years [*n* = 4] and 16 years and over [*n* = 1]), this meta-analysis was unable to evaluate any advances in self-evaluation ability and complexity of self-description that occur during adolescence. Additionally, maturity status likely influences self-perceptions and may moderate the associations with motor competence [198], although no studies within this review reported their findings in a way to examine this hypothesis. Therefore, future research should compare associations between motor competence and perceived motor competence by age group/stage of maturity.

The results of this meta-analysis may also reflect the alignment between actual and perceived motor competence measurements. For example, skills measured during actual motor competence assessments (e.g. Körperkoordinationstest Für Kinder—FMS) may not represent self-perceptions within existing broader measures (e.g. PSPP). Both Estevan and Barnett [22] and De Meester et al. [14] have recently advocated for better alignment between actual and perceived motor competence measurements. De Meester et al. [14] have called for authors to better articulate alignment and utilise different measures of perceived motor competence to assess the importance of alignment. Similarly, McGrane et al. [177] indicated the need for self-perception measures that capture differentiated perceptions of motor competence to a greater extent (e.g. PSPMSC—FMS). Thus, as our understanding of actual competence continues to develop (e.g. foundational movement skills [13], athleticism [18]), there is a need for commensurate development and alignment of perceived motor competence measurements with a particular research focus on process versus product motor competence measures and the variety of perceived motor competence measures available.

*Self-Efficacy/Confidence* Within this meta-analysis, only three studies reported associations between motor competence and self-efficacy/confidence. However, as per the definition of athleticism, youth engage with confidence as well as actual/perceived competence [1], suggesting that a greater understanding of this interaction is required. Again, as our understanding of actual competence develops, self-efficacy/confidence might be best understood related to specific motor competencies that are assessed. Consequently, more research exploring how perceived motor competence and self-efficacy/confidence are associated with actual motor competence is required.

*Motivation* Motor competence and motivation associations were trivial to small, with only overall competence represented by enough study samples (*n* = 4). Developing approaches to accurately determine motor competence can provide individuals with more realistic expectations of their competence, reduce the incidence of unsuccessful outcomes and reduce the incidence of lower motivation [36, 199]. Previous studies have found that significant amounts of autonomous motivation are explained by an adolescent’s perceived motor competence [200, 201]. However, most studies (3/5 studies) within this meta-analysis reported motivation via relative autonomy index scores. Thus, it is unclear how different components of motivation influence this association, although we hypothesise that greater motor competence is associated with greater autonomy for physical activity. Additional research is required to evaluate this association amongst adolescents and should account for the effect of perceived motor competence. Nevertheless, practitioners should promote success for all adolescents to encourage autonomous motivation and participation in physical activity, regardless of an individual’s actual motor competence [36, 200].

### Moderator Variables

Overall, potential moderators (i.e. sex, age, type of motor competence assessment) produced a limited influence on the strength or orientation of motor competence associations during adolescence. However, a moderator analysis identified three significant findings. First, the association between object control competence and physical activity was greater for male individuals compared with female individuals. During motor competence assessments, male individuals often outperform female individuals in power and strength tasks, while female individuals perform better than male individuals during fine motor tasks, flexibility and balance [202]. Three out of five studies in this meta-analysis compared male and female associations between object control competence and physical activity using product measures (e.g. throwing). Such skills are complex multi-segmental motions that require energy transfer and timing [203, 204]. Inadvertently, this may explain the presented sex difference, as object control tasks require a prerequisite of strength and power to achieve desired outcomes.

Second, the overall competence and physical activity association was greater in studies using product motor competence assessments versus process assessments. This difference contradicts recent evidence in children, which suggests that process and product assessments are poor at explaining the variance in children’s physical activity [205]. Thus, this finding may suggest that as individuals develop into adolescence, successful physical activity engagement is synonymous with an individual’s ability to perform desired outcomes within the activities being explored, regardless of the technique behind it.

Last, the overall competence and weight status association was greater for studies with a mean age between 13 and 15 years, compared with studies with a mean age between 11 and 12 years. Indeed, excess weight can hinder the long-term development of motor competence [206]. This finding may therefore represent the negative trajectories of the developmental model [21] (i.e. poor weight status and motor competence leads to reduced physical activity, fewer opportunities to develop motor competence, and therefore, results in further weight gain as an individual develops). However, caution is needed when interpreting this finding, owing to the large difference in study samples within this moderator comparison (13 study samples for age 13–15 years; six study samples for age 11–12 years). Therefore, future research should explore the effects of age on motor competence and weight status associations.

Overall, the limited moderator findings are attributed to a lack of study samples per moderator, association and motor competence domain to draw meaningful conclusions. Thus, this section highlights the need for research exploring potential moderators (i.e. age, sex and type of motor competence assessment) for each motor competence domain to fully understand any moderator effects.

### Strengths and Limitations

This systematic review with a meta-analysis is novel given the sole focus on adolescents owing to currently poor health-related trends during this stage of physical and psychosocial development. However, this review only included studies published in English, and important information may have been missed from non-English publications. Nevertheless, evidence was evaluated from 16 countries, which represents a broad overview of the associations between motor competence, physical activity, physical fitness and psychosocial characteristics in adolescence. Second, studies of physically/cognitively impaired adolescents were not included because of the already broad nature of this review. A previous review could not determine if the association between motor competence and perceived motor competence was stronger for typically developing or physically/cognitively impaired individuals because of a lack of study samples [14]. Consequently, it was beyond the scope of this review to further explore this comparison amongst adolescents. Nevertheless, this review highlighted the limited evidence regarding the influence of maturity status on the associations between motor competence and physical activity, physical fitness and psychosocial characteristics during adolescence. Furthermore, this review followed the updated PRISMA guidelines [48], included numerous database searches throughout the review process, followed clear and robust inclusion/exclusion criteria and utilised a second reviewer for screening purposes (title/abstract/full-text screening, study bias assessment).

## Practical Applications

This review with meta-analysis provides several practical applications. First, researchers should (where feasible) include longitudinal assessments across adolescence, utilise combined motor competence tools (i.e. process and product), report overall and process/product scores, report scores for different motor competence domains (e.g. object control, stability/balance) and consider how maturity status influences such associations. Second, those seeking to design interventions to improve health-related outcomes in adolescence should focus on the synergistic development of motor competence, physical fitness and psychosocial characteristics (rather than focusing on sports alone) to increase physical activity opportunities. Adolescence is a complex and challenging period of the lifespan consisting of physical [38, 39, 42] and psychosocial changes [43–45], and organisations and practitioners need to recognise such complexities and collaborate to support continual development. For example, reflecting on current motor competence, physical fitness and psychosocial practices, and evaluating the importance of these characteristics can spark awareness of the developmental needs for different stages of maturity (e.g. [207].). Recent research (e.g. [2, 208–210].) also clarifies the goals and realities of an adolescent’s long-term developmental needs and provides suitable recommendations that practitioners and organisations can adopt to promote a fitter, healthier and more physically active adolescent population. However, it is apparent that such research needs translating into useful resources for coaches, teachers and organisations to utilise within their environments.

## Conclusions

This paper aimed to (1) analyse the scientific literature evaluating associations between motor competence and physical activity, physical fitness and/or psychosocial characteristics amongst adolescents; (2) evaluate the associations between motor competence and physical activity, physical fitness characteristics and/or psychosocial characteristics amongst adolescents; and (3) investigate the impact of moderator variables (i.e. age, sex, type of motor competence assessment) on these associations. This study expands on previous reviews (e.g. [12, 25].), by focusing on adolescents, exploring broader physical fitness components of athleticism (e.g. muscular power, speed, agility) and discussing the potential influence of maturity status on associations. The risk of bias assessment highlighted suboptimal reporting of sampling methods, participant characteristics and the validity of physical activity/physical fitness/psychosocial measures. Furthermore, this review supports the need for longitudinal exploration of the Stodden et al. [21] developmental model during adolescence [12, 14, 25, 28, 29]. Present findings highlight several methodological differences when measuring motor competence, physical activity, physical fitness and psychosocial characteristics. Specifically, studies favoured either process or product motor competence evaluations, which when used independently, provide a limited overview of an individual’s motor competence [182]. Finally, the review showed that few studies considered the influence of maturity status on motor competence associations, even though adolescents can experience a transient decline in coordination during peak growth (i.e. circa-PHV; [12]).

The current meta-analyses support previous evidence [12, 14, 25–28, 47] exploring the hypothesised motor competence associations [21] and identified positive associations between motor competence and physical activity, composite fitness scores, muscular endurance, muscular power, muscular strength, cardiovascular endurance, perceived motor competence and motivation, in addition to inverse associations between motor competence and weight status, speed and agility. Interventions to enhance an adolescent’s health and well-being should synergistically target motor competence, physical and psychosocial development. However, improved evaluations of these characteristics are required to better inform such interventions during adolescence.

### Supplementary Information

Below is the link to the electronic supplementary material.

Supplementary file1 (DOCX 37 KB)

## Declarations

### Availability of Data and Material

The datasets generated and/or analysed during this study are available from the corresponding author upon reasonable request.
