# Supplementary material for: Associations Between Motor Competence and Physical Activity, Physical Fitness and Psychosocial Characteristics in Adolescents: A Systematic Review and Meta-analysis
Source: Sports Med. 2023 Aug 5;53(11):2191–256. doi: 10.1007/s40279-023-01886-1 (PMC10587315; doi:10.1007/s40279-023-01886-1)
Supplement: Supplementary file 1 — Supplementary file1 (DOCX 37 KB) [file 40279_2023_1886_MOESM1_ESM.docx]

| **Supplementary table 1**. Summary of moderator analysis for sex, age, and assessment type on the pooled correlation coefficients between motor competence, physical activity, physical fitness characteristics, and psychosocial characteristics. | | | |
| --- | --- | --- | --- |
| Association | Moderator | Between group comparison | Subgroup pooled effect size |
| Motor competence and physical activity | Sex | **Overall competence**  Females vs. Males *p* = 0.96  **Locomotor competence**  Females vs. Males *p* = 0.79  **Object control competence**  Females vs. Males ***p* = 0.04**  **Stability/balance competence**  Females vs. Males *p* = 0.98 | **Overall competence**  Females (Studies n = 4; Sample size n = 2208; *r* = 0.21; 95% CI [0.05, 0.35]; ***p* < 0.05**)  Males (Studies n = 5; Sample size n = 2568; *r* = 0.21; 95% CI [0.02, 0.39]; ***p* = 0.03**)  **Locomotor competence**  Females (Studies n = 3; Sample size n = 469; *r* = 0.26; 95% CI [0.18, 0.35]; ***p* < 0.01**)  Males (Studies n = 3; Sample size n = 443; *r* = 0.24; 95% CI [0.04, 0.42]; ***p* < 0.05**)  **Object control competence**  Females (Studies n = 5; Sample size n = 2284; *r* = 0.21; 95% CI [0.17, 0.25]; ***p* < 0.01**)  Males (Studies n = 5; Sample size n = 2266; *r* = 0.33; 95% CI [0.22, 0.42]; ***p* < 0.01**)  **Stability/balance competence**  Females (Studies n = 5; Sample size n = 3010; *r* = 0.23; 95% CI [0.18, 0.29]; ***p* < 0.01**)  Males (Studies n = 5; Sample size n = 3043; *r* = 0.23; 95% CI [0.11, 0.35]; ***p* < 0.01**) |
|  | Age | **Overall competence**  11-12 years vs. 13-15 years *p* = 0.51  **Locomotor competence**  11-12 years vs. ^a^13-15 years  11-12 years vs. ^a^16+ years  ^a^13-15 years vs. ^a^16+ years  **Object control competence**  11-12 years vs. 13-15 years *p* = 0.51  11-12 years vs. ^a^16+ years  13-15 years vs. ^a^16+ years  **Stability/balance competence**  11-12 years vs. 13-15 years *p* = 0.66 | **Overall competence**  11-12 (Studies n = 4; Sample size n = 512; *r* = 0.17; 95% CI [0.03, 0.30]; ***p* < 0.05**)  13-15 (Studies n = 9; Sample size n = 4712; *r* = 0.23; 95% CI [0.11, 0.33]; ***p* < 0.01**)  16+ (N/A)  **Locomotor competence**  11-12 (Studies n = 6; Sample size n = 912; *r* = 0.26; 95% CI [0.18, 0.34]; ***p* < 0.01**)  ^a^13-15 (Studies n = 1; Sample size n = 316; *r* = 0.08; 95% CI [−0.03, 0.19]; *p* = 0.16)  ^a^16+ (Studies n = 1; Sample size n = 215; *r* = 0.14; 95% CI [0.01, 0.27]; ***p* < 0.05**)  **Object control competence**  11-12 (Studies n = 6; Sample size n = 912; *r* = 0.22; 95% CI [0.09, 0.34]; ***p* < 0.01**)  13-15 (Studies n = 5; Sample size n = 3954; *r* = 0.27; 95% CI [0.16, 0.38]; ***p* < 0.01**)  ^a^16+ (Studies n = 1; Sample size n = 215; *r* = 0.35; 95% CI [0.23, 0.46]; ***p* < 0.01**)  **Stability/balance competence**  11-12 (Studies n = 4; Sample size n = 576; *r* = 0.16; 95% CI [−0.02, 0.33]; *p* = 0.08)  13-15 (Studies n = 7; Sample size n = 5793; *r* = 0.21; 95% CI [0.13, 0.29]; ***p* < 0.01**)  16+ (N/A) |
|  | Assessment type | **Overall competence**  ^a^Combined vs. Process  ^a^Combined vs. Product  Process vs. Product ***p* = 0.03**  **Locomotor competence**  Process vs. Product *p* = 0.15  **Object control competence**  Process vs. Product *p* = 0.44  **Stability/balance competence**  ^a^Process vs. Product | **Overall competence** ^a^Combined (Studies n = 2; Sample size n = 692; *r* = −0.01; 95% CI [−0.08, 0.06]; *p* = 0.79)  Process (Studies n = 5; Sample size n = 621; *r* = 0.18; 95% CI [0.08, 0.27]; ***p* < 0.01**)  Product (Studies n = 6; Sample size n = 3911; *r* = 0.31; 95% CI [0.23, 0.39]; ***p* < 0.01**)  **Locomotor competence**  Combined (N/A)  Process (Studies n = 3; Sample size n = 300; *r* = 0.13; 95% CI [0.01, 0.24]; ***p* < 0.05**)  Product (Studies n = 5; Sample size n = 1143; *r* = 0.24; 95% CI [0.13, 0.35]; ***p* < 0.01**)  **Object control competence**  Combined (N/A)  Process (Studies n = 3; Sample size n = 300; *r* = 0.14; 95% CI [−0.20, 0.45]; *p* = 0.41)  Product (Studies n = 9; Sample size n = 4781; *r* = 0.27; 95% CI [0.19, 0.35]; ***p* < 0.01**)  **Stability/balance competence**  Combined (N/A)  ^a^Process (Studies n = 2; Sample size n = 85; *r* = −0.07; 95% CI [−0.29, 0.14]; *p* = 0.51)  Product (Studies n = 9; Sample size n = 6284; *r* = 0.22; 95% CI [0.15, 0.29]; ***p* < 0.01**) |
| Motor competence and composite fitness score | Sex | **Overall competence**  Females vs. Males *p* = 0.56 | **Overall competence**  Females (Studies n = 4; Sample size n = 166; *r* = 0.39; 95% CI [0.25, 0.51]; ***p*** **< 0.01**)  Males (Studies n = 6; Sample size n = 584; *r* = 0.44; 95% CI [0.33, 0.54]; ***p* < 0.01**) |
|  | Age | **Overall competence**  11-12 years vs. 13-15 years *p* = 0.24 | **Overall competence**  11-12 (Studies n = 5; Sample size n = 801; *r* = 0.47; 95% CI [0.30, 0.61]; ***p* < 0.01**)  13-15 (Studies n = 7; Sample size n = 345; *r* = 0.35; 95% CI [0.26, 0.45]; ***p* < 0.01**)  16+ (N/A) |
|  | Assessment type | **Overall competence**  ^a^Process vs. Product | **Overall competence**  Combined (N/A)  ^a^Process (Studies n = 2; Sample size n = 424; *r* = 0.43; 95% CI [0.34, 0.50]; ***p* < 0.01**)  Product (Studies n = 10; Sample size n = 722; *r* = 0.39; 95% CI [0.28, 0.49]; ***p* < 0.01**) |
| Motor competence and weight status | Sex | **Overall competence**  Females vs. Males *p* = 0.80  **Locomotor competence**  ^a^Females vs. ^a^Males  **Object control competence**  Females vs. Males *p* = 0.69  **Stability/balance competence**  Females vs. Males *p* = 0.37 | **Overall competence**  Females (Studies n = 7; Sample size n = 3017; *r* = −0.20; 95% CI [−0.30, −0.10]; ***p* < 0.01**)  Males (Studies n = 6; Sample size n = 3108; *r* = −0.19; 95% CI [−0.26, −0.11]; ***p* < 0.01**)  **Locomotor competence**  ^a^Females (Studies n = 1; Sample size n = 31; *r* = −0.34; 95% CI [−0.62, 0.02]; *p* = 0.06)  ^a^Males (Studies n = 1; Sample size n = 54; *r* = −0.38; 95% CI [−0.58, −0.12]; ***p* < 0.01**)  **Object control competence**  Females (Studies n = 3; Sample size n = 1846; *r* = −0.09; 95% CI [−0.14, −0.04]; ***p* < 0.01**)  Males (Studies n = 3; Sample size n = 1877; *r* = −0.10; 95% CI −0.15, −0.06]; ***p* < 0.01**)  **Stability/balance competence**  Females (Studies n = 4; Sample size n = 2735; *r* = −0.16; 95% CI [−0.24, −0.08]; ***p* < 0.01**)  Males (Studies n = 4; Sample size n = 2827; *r* = −0.22; 95% CI [−0.30, −0.13]; ***p* < 0.01**) |
|  | Age | **Overall competence**  11-12 years vs. 13-15 years ***p* = 0.03**  **Object control competence**  ^a^11-12 years vs. 13-15 years  **Stability/balance competence**  ^a^11-12 years vs. 13-15 years | **Overall competence**  11-12 (Studies n = 6; Sample size n = 1003; *r* = −0.21; 95% CI [−0.33, −0.10]; ***p* < 0.01**)  13-15 (Studies n = 17; Sample size n = 6166; *r* = −0.37; 95% CI [−0.46, −0.28]; ***p* < 0.01**)  16+ (N/A)  **Locomotor competence**  ^a^11-12 (Studies n = 2; Sample size n = 85; *r* = −0.36; 95% CI [−0.54, −0.16]; ***p* < 0.01**)  13-15 (N/A)  16+ (N/A)  **Object control competence**  ^a^11-12 (Studies n = 2; Sample size n = 85; *r* = −0.09; 95% CI [−0.30, 0.13]; *p* = 0.42)  13-15 (Studies n = 4; Sample size n = 3638; *r* = −0.10; 95% CI [−0.13, -0.06]; ***p* < 0.01**)  16+ (N/A)  **Sports specific competence**  ^a^11-12 (Studies n = 1; Sample size n = 279; *r* = −0.15; 95% CI [−0.26, −0.03]; ***p* < 0.05**)  13-15 (N/A)  16+ (N/A)  **Stability/balance competence**  ^a^11-12 (Studies n = 2; Sample size n = 85; *r* = −0.21; 95% CI [−0.70, 0.43]; *p* = 0.54)  13-15 (Studies n = 6; Sample size n = 5477; *r* = −0.18; 95% CI [−0.23, −0.14]; ***p* < 0.01**)  16+ (N/A) |
|  | Assessment type | **Overall competence**  ^a^Combined vs. Process  ^a^Combined vs. Product  Process vs. Product *p* = 0.20  **Object control competence**  ^a^Process vs. Product  **Stability/balance competence**  ^a^Process vs. Product | **Overall competence**  ^a^Combined (Studies n = 2; Sample size n = 1491; *r* = −0.10; 95% CI [−0.15, −0.05]; ***p* < 0.01**)  Process (Studies n = 9; Sample size n = 1161; *r* = −0.31; 95% CI [−0.45, −0.15]; ***p* < 0.01**)  Product (Studies n = 13; Sample size n = 4585; *r* = −0.42; 95% CI [−0.52, −0.32]; ***p* < 0.01**)  **Locomotor competence**  Combined (N/A)  ^a^Process (Studies n = 2; Sample size n = 85; *r* = −0.36; 95% CI [−0.54, −0.16]; ***p* < 0.01**)  Product (N/A)  **Object control competence**  Combined (N/A)  ^a^Process (Studies n = 2; Sample size n = 85; *r* = −0.09; 95% CI [−0.30, 0.13]; *p* = 0.42)  Product (Studies n = 4; Sample size n = 3638; *r* = −0.10; 95% CI [−0.13, −0.06]; ***p* < 0.01**)  **Sports specific competence**  Combined (N/A)  ^a^Process (Studies n = 1; Sample size n = 279; *r* = −0.15; 95% CI [−0.26, −0.03]; ***p* < 0.05**)  Product (N/A)  **Stability/balance competence**  Combined (N/A)  ^a^Process (Studies n = 2; Sample size n = 85; *r* = −0.21; 95% CI [−0.70, 0.43]; *p* = 0.54)  Product (Studies n = 6; Sample size n = 5477; *r* = −0.18; 95% CI [−0.23, -0.14]; ***p* < 0.01**) |
| Motor competence and muscular endurance | Sex | **Overall competence**  ^a^Females vs. ^a^Males  **Locomotor competence**  ^a^Females vs. ^a^Males  **Object control competence**  ^a^Females vs. ^a^Males  **Stability/balance competence**  ^a^Females vs. ^a^Males | **Overall competence**  ^a^Females (Studies n = 1; Sample size n = 763; *r* = 0.27; 95% CI [0.20, 0.33]; ***p* < 0.01**)  ^a^Males (Studies n = 1; Sample size n = 808; *r* = 0.37; 95% CI [0.31, 0.43]; ***p* < 0.01**)  **Locomotor competence**  ^a^Females (Studies n = 1; Sample size n = 275; *r* = 0.56; 95% CI [0.47, 0.64]; ***p* < 0.01**)  ^a^Males (Studies n = 1; Sample size n = 216; *r* = 0.47; 95% CI [0.36, 0.57]; ***p* < 0.01**)  **Object control competence**  ^a^Females (Studies n = 1; Sample size n = 275; *r* = 0.33; 95% CI [0.22, 0.43]; ***p* < 0.01**)  ^a^Males (Studies n = 1; Sample size n = 216; *r* = 0.36; 95% CI [0.24, 0.47]; ***p* < 0.01**)  **Stability/balance competence**  ^a^Females (Studies n = 1; Sample size n = 275; *r* = 0.55; 95% CI [0.46, 0.63]; ***p* < 0.01**)  ^a^Males (Studies n = 1; Sample size n = 216; *r* = 0.48; 95% CI [0.37, 0.58]; ***p* < 0.01**) |
|  | Age | Not enough data to compare moderator subgroups | **Overall competence**  11-12 (N/A)  13-15 (Studies n = 5; Sample size n = 1804; *r* = 0.34; 95% CI [0.27, 0.41]; ***p* < 0.01**)  16+ (N/A)  **Locomotor competence**  ^a^11-12 (Studies n = 2; Sample size n = 491; *r* = 0.52; 95% CI [0.43, 0.60]; ***p* < 0.01**)  13-15 (N/A)  16+ (N/A)  **Object control competence**  ^a^11-12 (Studies n = 2; Sample size n = 491; *r* = 0.34; 95% CI [0.26, 0.42]; ***p* < 0.01**)  13-15 (N/A)  16+ (N/A)  **Sports specific competence**  ^a^11-12 (Studies n = 1; Sample size n = 279; *r* = 0.36; 95% CI [0.25, 0.46]; ***p* < 0.01**)  13-15 (N/A)  16+ (N/A)  **Stability/balance competence**  ^a^11-12 (Studies n = 2; Sample size n = 491; *r* = 0.52; 95% CI [0.45, 0.58]; ***p* < 0.01**)  13-15 (N/A)  16+ (N/A) |
|  | Assessment type | **Overall competence**  ^a^Combined vs. ^a^Process  ^a^Combined vs. ^a^Product  ^a^Process vs. ^a^Product | **Overall competence**  ^a^Combined (Studies n = 2; Sample size n = 1571; *r* = 0.32; 95% CI [0.22, 0.42]; ***p* < 0.01**)  ^a^Process (Studies n = 2; Sample size n = 171; *r* = 0.33; 95% CI [0.19, 0.46]; ***p* < 0.01**)  ^a^Product (Studies n = 1; Sample size n = 62; *r* = 0.49; 95% CI [0.27, 0.66]; ***p* < 0.01**)  **Locomotor competence**  Combined (N/A)  Process (N/A)  ^a^Product (Studies n = 4; Sample size n = 1781; *r* = 0.44; 95% CI [0.31, 0.55]; ***p* < 0.01**)  **Object control competence**  Combined (N/A)  Process (N/A)  ^a^Product (Studies n = 4; Sample size n = 1781; *r* = 0.31; 95% CI [0.27, 0.35]; ***p* < 0.01**)  **Sports specific competence**  Combined (N/A)  ^a^Process (Studies n = 1; Sample size n = 279; *r* = 0.36; 95% CI [0.25, 0.46]; ***p* < 0.01**)  Product (N/A)  **Stability/balance competence**  ^a^11-12 (Studies n = 2; Sample size n = 491; *r* = 0.52; 95% CI [0.45, 0.58]; ***p* < 0.01**)  13-15 (N/A)  16+ (N/A) |
| Motor competence and muscular power | Sex | **Overall competence**  Females vs. Males *p* = 0.19  **Stability/balance competence**  ^a^Females vs. ^a^Males | **Overall competence** Females (Studies n = 3; Sample size n = 161; *r* = 0.00; 95% CI [−0.47, 0.47]; *p* = 1.00)  Males (Studies n = 9; Sample size n = 695; *r* = 0.35; 95% CI [0.19, 0.49]; ***p* < 0.01**)  **Stability/balance competence**  ^a^Females (Studies n = 2; Sample size n = 56; *r* = −0.06; 95% CI [−0.33, 0.21]; *p* = 0.66)  ^a^Males (Studies n = 2; Sample size n = 56; *r* = 0.07; 95% CI [−0.20, 0.33]; *p* = 0.62) |
|  | Age | **Overall competence**  ^a^11-12 vs. 13-15  ^a^11-12 vs. 16+  13-15 years vs. 16+ years *p* = 0.16 | **Overall competence**  11-12 (Studies n = 2; Sample size n = 224; *r* = 0.41; 95% CI [0.30, 0.52]; ***p* < 0.01**)  13-15 (Studies n = 5; Sample size n = 563; *r* = 0.24; 95% CI [0.08, 0.39]; ***p* < 0.05**)  16+ (Studies n = 4; Sample size n = 112; *r* = 0.01; 95% CI [−0.27, 0.29]; *p* = 0.96)  **Stability/balance competence**  11-12 (N/A)  13-15 (N/A)  16+ (Studies n = 5; Sample size n = 144; *r* = 0.03; 95% CI [−0.14, 0.20]; *p* = 0.69) |
|  | Assessment type | Not enough data to compare moderator subgroups | **Overall competence**  Combined (N/A)  Process (Studies n = 13; Sample size n = 965; *r* = 0.29; 95% CI [0.16, 0.42]; ***p* < 0.01**)  Product (N/A)  **Stability/balance competence**  Combined (N/A)  Process (N/A)  Product (Studies n = 5; Sample size n = 144; *r* = 0.03; 95% CI [−0.14, 0.20]; *p* = 0.69) |
| Motor competence and speed | Sex | Not enough data to compare moderator subgroups | **Overall competence**  Females (N/A)  ^a^Males (Studies n = 4; Sample size n = 376; *r* = −0.31; 95% CI [−0.40, −0.21]; ***p* < 0.01**) |
|  | Age | Not enough data to compare moderator subgroups | **Overall competence**  11-12 (N/A)  13-15 (Studies n = 4; Sample size n = 376; *r* = −0.31; 95% CI [−0.40, −0.21]; ***p* < 0.01**)  16+ (N/A) |
|  | Assessment type | Not enough data to compare moderator subgroups | **Overall competence**  Combined (N/A)  Process (Studies n = 4; Sample size n = 376; *r* = −0.31; 95% CI [−0.40, −0.21]; ***p* < 0.01**)  Product (N/A) |
| Motor competence and agility | Sex | **Overall competence**  ^a^Females vs. ^a^Males  **Stability/balance competence**  ^a^Females vs. ^a^Males | **Overall competence** ^a^Females (Studies n = 1; Sample size n = 28; *r* = −0.08; 95% CI [−0.44, 0.30]; *p* = 0.67)  ^a^Males (Studies n = 2; Sample size n = 61; *r* = −0.49; 95% CI [−0.67, −0.27]; ***p* < 0.01**)  **Stability/balance competence**  ^a^Females (Studies n = 1; Sample size n = 28; *r* = −0.45; 95% CI [−0.70, −0.09]; ***p* < 0.05**)  ^a^Males (Studies n = 1; Sample size n = 28; *r* = −0.10; 95% CI [−0.46, 0.28]; *p* = 0.61) |
|  | Age | Not enough data to compare moderator subgroups | **Overall competence**  11-12 (N/A)  13-15 (N/A)  ^a^16+ (Studies n = 2; Sample size n = 56; *r* = −0.27; 95% CI [−0.57, 0.10]; *p* = 0.15)  **Stability/balance competence**  11-12 (N/A)  13-15 (N/A)  16+ (Studies n = 3; Sample size n = 88; *r* = −0.21; 95% CI [−0.44, 0.04]; *p* = 0.09) |
|  | Assessment type | **Overall competence**  ^a^Process vs. ^a^Product | **Overall competence**  Combined (N/A)  Process (Studies n = 1; Sample size n = 33; *r* = −0.54; 95% CI [−0.75, −0.24]; ***p* < 0.01**)  ^a^Product (Studies n = 2; Sample size n = 56; *r* = −0.27; 95% CI [−0.57, 0.10]; *p* = 0.15)  **Stability/balance competence**  Combined (N/A)  Process (N/A)  Product (Studies n = 3; Sample size n = 88; *r* = −0.21; 95% CI [−0.44, 0.04]; *p* = 0.09) |
| Motor competence and strength | Sex | **Overall competence**  Females vs. Males *p* = 0.78  **Stability/balance competence**  ^a^Females vs. ^a^Males | **Overall competence**  Females (Studies n = 3; Sample size n = 977; *r* = 0.34; 95% CI [0.28, 0.39]; ***p* < 0.01**)  Males (Studies n = 3; Sample size n = 1032; *r* = 0.31; 95% CI [0.14, 0.47]; ***p* < 0.01**)  **Stability/balance competence**  ^a^Females (Studies n = 2; Sample size n = 1778; *r* = 0.45; 95% CI [0.35, 0.54]; ***p* < 0.01**)  ^a^Males (Studies n = 2; Sample size n = 1900; *r* = 0.38; 95% CI [0.34, 0.41]; ***p* < 0.01**) |
|  | Age | Not enough data to compare moderator subgroups | **Overall competence**  11-12 (N/A)  13-15 (Studies n = 10; Sample size n = 2257; *r* = 0.36; 95% CI [0.30, 0.41]; ***p* < 0.01**)  16+ (N/A)  **Stability competence** 11-12 (N/A)  13-15 (Studies n = 4; Sample size n = 3678; *r* = 0.41; 95% CI [0.35, 0.47]; ***p* < 0.01**)  16+ (N/A) |
|  | Assessment type | **Overall competence**  ^a^Combined vs. Process  ^a^Combined vs. ^a^Product  Process vs. ^a^Product | **Overall competence**  ^a^Combined (Studies n = 2; Sample size n = 1585; *r* = 0.38; 95% CI [0.28, 0.47]; ***p* < 0.01**)  Process (Studies n = 6; Sample size n = 548; *r* = 0.31; 95% CI [0.23, 0.38]; ***p* < 0.01**)  ^a^Product (Studies n = 2; Sample size n = 124; *r* = 0.41; 95% CI [0.25, 0.55]; ***p* < 0.01**)  **Stability competence**  Combined (N/A)  Process (N/A)  Product (Studies n = 4; Sample size n = 3678; *r* = 0.41; 95% CI [0.35, 0.47]; ***p* < 0.01**) |
| Motor competence and cardiovascular endurance | Sex | **Overall competence**  Females vs. Males *p* = 0.61  **Locomotor competence**  ^a^Females vs. ^a^Males  **Object control competence**  ^a^Females vs. ^a^Males  **Stability/balance competence**  ^a^Females vs. ^a^Males | **Overall competence**  Females (Studies n = 3; Sample size n = 1670; *r* = 0.37; 95% CI [0.14, 0.57]; ***p* < 0.01**)  Males (Studies n = 4; Sample size n = 1967; *r* = 0.31; 95% CI [0.22, 0.40]; ***p* < 0.01**)  **Locomotor competence**  ^a^Females (Studies n = 1; Sample size n = 275; *r* = 0.60; 95% CI [0.52, 0.67]; ***p* < 0.01**)  ^a^Males (Studies n = 1; Sample size n = 216; *r* = 0.61; 95% CI [0.52, 0.69]; ***p* < 0.01**)  **Object control competence**  ^a^Females (Studies n = 1; Sample size n = 275; *r* = 0.45; 95% CI [0.35, 0.54]; ***p* < 0.01**)  ^a^Males (Studies n = 1; Sample size n = 216; *r* = 0.55; 95% CI [0.45, 0.64]; ***p* < 0.01**)  **Stability/balance competence**  ^a^Females (Studies n = 2; Sample size n = 1164; *r* = 0.48; 95% CI [0.44, 0.53]; ***p* < 0.01**)  ^a^Males (Studies n = 2; Sample size n = 1166; *r* = 0.48; 95% CI [0.25, 0.66]; ***p* < 0.01**) |
|  | Age | **Stability/balance competence**  ^a^11-12 vs. ^a^13-15 | **Overall competence**  11-12 (N/A)  13-15 (Studies n = 12; Sample size n = 3994; *r* = 0.37; 95% CI [0.28, 0.45]; ***p* < 0.01**)  16+ (N/A)  **Locomotor competence**  ^a^11-12 (Studies n = 2; Sample size n = 491; *r* = 0.60; 95% CI [0.54, 0.66]; ***p* < 0.01**)  13-15 (N/A)  16+ (N/A)  **Object control competence**  ^a^11-12 (Studies n = 2; Sample size n = 491; *r* = 0.50; 95% CI [0.39, 0.59]; ***p* < 0.01**)  13-15 (N/A)  16+ (N/A)  **Sports specific competence**  ^a^11-12 (Studies n = 1; Sample size n = 279; *r* = 0.38; 95% CI [0.27, 0.48]; ***p* < 0.01**)  13-15 (N/A)  16+ (N/A)  **Stability/balance competence**  ^a^11-12 (Studies n = 2; Sample size n = 491; *r* = 0.53; 95% CI [0.44, 0.62]; ***p* < 0.01**)  ^a^13-15 (Studies n = 2; Sample size n = 1839; *r* = 0.43; 95% CI [0.31, 0.53]; ***p* < 0.01**)  16+ (N/A) |
|  | Assessment type | **Overall competence**  ^a^Combined vs. Process  ^a^Combined vs. ^a^Product  Process vs. ^a^Product | **Overall competence** ^a^Combined (Studies n = 2; Sample size n = 1503; *r* = 0.19; 95% CI [0.12, 0.25]; ***p* < 0.01**)  Process (Studies n = 8; Sample size n = 2367; *r* = 0.40; 95% CI [0.32, 0.47]; ***p* < 0.01**)  ^a^Product (Studies n = 2; Sample size n = 124; *r* = 0.47; 95% CI [−0.04, 0.79]; *p* = 0.07)  **Locomotor competence**  Combined (N/A)  Process (N/A)  ^a^Product (Studies n = 2; Sample size n = 491; *r* = 0.60; 95% CI [0.54, 0.66]; ***p* < 0.01**)  **Object control competence**  Combined (N/A)  Process (N/A)  ^a^Product (Studies n = 2; Sample size n = 491; *r* = 0.50; 95% CI [0.39, 0.59]; ***p* < 0.01**)  **Sports specific competence**  Combined (N/A)  ^a^Process (Studies n = 1; Sample size n = 279; *r* = 0.38; 95% CI [0.27, 0.48]; ***p* < 0.01**)  Product (N/A)  **Stability/balance competence**  Combined (N/A)  Process (N/A)  ^a^Product (Studies n = 4; Sample size n = 2330; *r* = 0.48; 95% CI [0.39, 0.55]; ***p* < 0.01**) |
| Motor competence and flexibility | Sex | **Overall competence**  **^a^**Females vs. ^a^Males  **Stability/balance competence**  **^a^**Females vs. ^a^Males | **Overall competence**  Females (Studies n = 1; Sample size n = 768; *r* = 0.22; 95% CI [0.15, 0.29]; ***p* < 0.01**)  Males (Studies n = 1; Sample size n = 813; *r* = 0.26; 95% CI [0.19, 0.32]; ***p* < 0.01**)  **Stability/balance competence**  ^a^Females (Studies n = 1; Sample size n = 889; *r* = 0.23; 95% CI [0.17, 0.29]; ***p* < 0.01**)  ^a^Males (Studies n = 1; Sample size n = 950; *r* = 0.10; 95% CI [0.04, 0.16]; ***p* < 0.01**) |
|  | Age | Not enough data to compare moderator subgroups | **Overall competence**  11-12 (N/A)  ^a^13-15 (Studies n = 3; Sample size n = 1690; *r* = 0.23; 95% CI [0.19, 0.28]; ***p* < 0.01**)  16+ (N/A)  **Sports specific competence**  ^a^11-12 (Studies n = 1; Sample size n = 279; *r* = −0.07; 95% CI [−0.19, 0.05]; *p* = 0.24)  13-15 (N/A)  16+ (N/A)  **Stability/balance competence**  11-12 (N/A)  ^a^13-15 (Studies n = 2; Sample size n = 1839; *r* = 0.17; 95% CI [0.04, 0.29]; ***p* < 0.05**)  16+ (N/A) |
|  | Assessment type | **Overall competence**  ^a^Combined vs. ^a^Process | **Overall competence** ^a^Combined (Studies n = 2; Sample size n = 1581; *r* = 0.24; 95% CI [0.19, 0.29]; ***p* < 0.01**)  ^a^Process (Studies n = 1; Sample size n = 109; *r* = 0.14; 95% CI [−0.05, 0.32]; *p* = 0.14)  Product (N/A)  **Sports specific competence**  Combined (N/A)  ^a^Process (Studies n = 1; Sample size n = 279; *r* = −0.07; 95% CI [−0.19, 0.05]; *p* = 0.24)  Product (N/A)  **Stability/balance competence**  Combined (N/A)  Process (N/A)  ^a^Product (Studies n = 2; Sample size n = 1839; *r* = 0.17; 95% CI [0.04, 0.29]; ***p* < 0.05**) |
| Motor competence and perceived motor competence | Sex | **Overall competence**  Females vs. ^a^Males  **Stability/balance competence**  ^a^Females vs. ^a^Males | **Overall competence**  Females (Studies n = 5; Sample size n = 652; *r* = 0.30; 95% CI [0.23, 0.37]; ***p* < 0.01**)  Males (Studies n = 2; Sample size n = 158; *r* = 0.33; 95% CI [0.18, 0.46]; ***p* < 0.01**)  **Locomotor competence**  Females (N/A)  Males (N/A)  **Object control competence**  Females (N/A)  Males (N/A)  **Stability/balance competence**  ^a^Females (Studies n = 1; Sample size n = 889; *r* = 0.34; 95% CI [0.28, 0.40]; ***p* < 0.01**)  ^a^Males (Studies n = 1; Sample size n = 950; *r* = 0.28; 95% CI [0.22, 0.34]; ***p* < 0.01**) |
|  | Age | **Overall competence**  11-12 years vs. ^a^13-15 years  **Locomotor competence**  ^a^13-15 years vs. ^a^16+ years  **Object control competence**  13-15 years vs. ^a^16+ years | **Overall competence**  11-12 (Studies n = 8; Sample size n = 876; *r* = 0.31; 95% CI [0.25, 0.37]; ***p* < 0.01**)  ^a^13-15 (Studies n = 2; Sample size n = 440; *r* = 0.34; 95% CI [0.25, 0.42]; ***p* < 0.01**)  16+ N/A  **Locomotor competence**  11-12 (N/A)  ^a^13-15 (Studies n = 2; Sample size n = 686; *r* = 0.22; 95% CI [0.15, 0.29]; ***p* < 0.01**)  ^a^16+ (Studies n = 1; Sample size n = 215; *r* = 0.30; 95% CI [0.17, 0.42]; ***p* < 0.01**)  **Object control competence**  11-12 (N/A)  13-15 (Studies n = 3; Sample size n = 779; *r* = 0.28; 95% CI [0.15, 0.39]; ***p* < 0.01**)  ^a^16+ (Studies n = 1; Sample size n = 215; *r* = 0.46; 95% CI [0.35, 0.56]; ***p* < 0.01**)  **Stability/balance competence**  11-12 (N/A)  13-15 (Studies n = 5; Sample size n = 2618; *r* = 0.26; 95% CI [0.17, 0.34]; ***p* < 0.01**)  16+ (N/A) |
|  | Assessment type | **Overall competence**  Process vs. Product *p* = 0.94  **Locomotor competence**  ^a^Process vs. Product  **Object control competence**  ^a^Process vs. Product | **Overall competence**  Combined (N/A)  Process (Studies n = 9; Sample size n = 1082; *r* = 0.34; 95% CI [0.28, 0.41]; ***p* < 0.01**)  Product (Studies n = 4; Sample size n = 507; *r* = 0.34; 95% CI [0.26, 0.42]; ***p* < 0.01**)  **Locomotor competence**  Combined (N/A)  ^a^Process (Studies n = 1; Sample size n = 215; *r* = 0.30; 95% CI [0.17, 0.42]; ***p* < 0.01**)  Product (Studies n = 3; Sample size n = 1331; *r* = 0.24; 95% CI [0.19, 0.29]; ***p* < 0.01**)  **Object control competence**  Combined (N/A)  ^a^Process (Studies n = 1; Sample size n = 215; *r* = 0.46; 95% CI [0.35, 0.56]; ***p* < 0.01**)  Product (Studies n = 4; Sample size n = 1424; *r* = 0.30; 95% CI [0.20, 0.40]; ***p* < 0.01**)  **Stability/balance competence**  Combined (N/A)  Process (N/A)  Product (Studies n = 5; Sample size n = 2618; *r* = 0.26; 95% CI [0.17, 0.34]; ***p* < 0.01**) |
| Motor competence and self-efficacy/confidence | Sex | **Overall competence**  ^a^Females vs. ^a^Males | **Overall competence**  ^a^Females (Studies n = 1; Sample size n = 152; *r* = 0.31; 95% CI [0.15, 0.44]; ***p* < 0.01**)  ^a^Males (Studies n = 1; Sample size n = 157; *r* = 0.10; 95% CI [−0.06, 0.25]; *p* = 0.21) |
|  | Age | **Overall competence**  ^a^11-12 vs. ^a^13-15 | **Overall competence**  ^a^11-12 (Studies n = 1; Sample size n = 66; *r* = −0.12; 95% CI [−0.35, 0.13]; *p* = 0.34)  ^a^13-15 (Studies n = 8; Sample size n = 1055; *r* = 0.25; 95% CI [0.17, 0.32]; ***p* < 0.01**)  16+ (N/A) |
|  | Assessment type | Not enough data to compare moderator subgroups | **Overall competence**  Combined (N/A)  ^a^Process (Studies n = 9; Sample size n = 1121; *r* = 0.22; 95% CI [0.13, 0.31]; ***p* < 0.01**)  Product (N/A) |
| Motor competence and motivation | Sex | **Overall competence**  ^a^Females vs. ^a^Males | **Overall competence**  ^a^Females (Studies n = 1; Sample size n = 105; *r* = 0.26; 95% CI [0.07, 0.43]; ***p* < 0.05**)  ^a^Males (Studies n = 1; Sample size n = 119; *r* = 0.25; 95% CI [0.07, 0.41]; ***p* < 0.05**) |
|  | Age | **Overall competence**  ^a^11-12 vs. ^a^13-15 | **Overall competence**  ^a^11-12 (Studies n = 2; Sample size n = 224; *r* = 0.25; 95% CI [0.13, 0.37]; ***p* < 0.01**)  ^a^13-15 (Studies n = 2; Sample size n = 439; *r* = 0.17; 95% CI [0.08, 0.26]; ***p* < 0.01**)  16+ (N/A)  **Locomotor competence**  11-12 (N/A)  ^a^13-15 (Studies n = 2; Sample size n = 686; *r* = 0.15; 95% CI [0.08, 0.22]; ***p* < 0.01**)  16+ (N/A)  **Object control competence**  11-12 (N/A)  ^a^13-15 (Studies n = 2; Sample size n = 686; *r* = 0.07; 95% CI [−0.01, 0.14]; *p* = 0.07)  16+ (N/A)  **Stability/balance competence**  11-12 (N/A)  ^a^13-15 (Studies n = 2; Sample size n = 686; *r* = 0.20; 95% CI [0.13, 0.27]; ***p* < 0.01**)  16+ (N/A) |
|  | Assessment type | **Overall competence**  ^a^Process vs. ^a^Product | **Overall competence**  Combined (N/A)  ^a^Process (Studies n = 2; Sample size n = 224; *r* = 0.25; 95% CI [0.13, 0.37]; ***p* < 0.01**)  ^a^Product (Studies n = 2; Sample size n = 439; *r* = 0.17; 95% CI [0.08, 0.26]; ***p* < 0.01**)  **Locomotor competence**  Combined (N/A)  Process (N/A)  ^a^Product (Studies n = 2; Sample size n = 686; *r* = 0.15; 95% CI [0.08, 0.22]; ***p* < 0.01**)  **Object control competence**  Combined (N/A)  Process (N/A)  ^a^Product (Studies n = 2; Sample size n = 686; *r* = 0.07; 95% CI [−0.01, 0.14]; *p* = 0.07)  **Stability/balance competence**  Combined (N/A)  Process (N/A)  ^a^Product (Studies n = 2; Sample size n = 686; *r* = 0.20; 95% CI [0.13, 0.27]; ***p* < 0.01**) |
| Sex subgroups: Males, and females. Age subgroups: 11-12 years, 13-15 years, and 16+ years. Assessment type subgroups: Combined, process, and product. ^a^ = fewer than three study samples available; N/A = No data available for the subgroup; *r* = pooled correlation coefficient; 95% CI = 95% confidence interval; bold font = *p* < 0.05. | | | |
